# Genomic regions responsible for amenability to *Agrobacterium*-mediated transformation in barley

**DOI:** 10.1038/srep37505

**Published:** 2016-11-22

**Authors:** Hiroshi Hisano, Kazuhiro Sato

**Affiliations:** 1 Institute of Plant Science and Resources, Okayama University, 2-20-1 Chuo, Kurashiki, Okayama 710-0046 Japan

## Abstract

Different plant cultivars of the same genus and species can exhibit vastly different genetic transformation efficiencies. However, the genetic factors underlying these differences in transformation rate remain largely unknown. In barley, ‘Golden Promise’ is one of a few cultivars reliable for *Agrobacterium*-mediated transformation. By contrast, cultivar ‘Haruna Nijo’ is recalcitrant to genetic transformation. We identified genomic regions of barley important for successful transformation with *Agrobacterium*, utilizing the ‘Haruna Nijo’ × ‘Golden Promise’ F_2_ generation and genotyping by 124 genome-wide SNP markers. We observed significant segregation distortions of these markers from the expected 1:2:1 ratio toward the ‘Golden Promise’-type in regions of chromosomes 2H and 3H, indicating that the alleles of ‘Golden Promise’ in these regions might contribute to transformation efficiency. The same regions, which we termed *Transformation Amenability (TFA*) regions, were also conserved in transgenic F_2_ plants generated from a ‘Morex’ × ‘Golden Promise’ cross. The genomic regions identified herein likely include necessary factors for *Agrobacterium*-mediated transformation in barley. The potential to introduce these loci into any haplotype of barley opens the door to increasing the efficiency of transformation for target alleles into any haplotype of barley by the *TFA*-based methods proposed in this report.

Cultivated barley (*Hordeum vulgare*) is diploid cereal crop with a well-characterized genome of 5.1 Gbp^1^. Barley ranks fourth among cereals in production worldwide^2^ and is used for food, brewing and animal feed. Due to its importance as a crop, numerous agronomical and industrial traits of barley have been genetically analyzed. Genetic transformation is an essential tool in genetics; incorporating DNA into an organism’s genome allows examination of endogenous genes as well as introduction of novel traits. Unfortunately, genetic analysis in barley remains limited by the technical challenges involved in transformation, in stark contrast to the ease of *Agrobacterium-*mediated transformation in other plant species such as *Arabidopsis thaliana* and rice (*Oryza sativa*). The most commonly employed method of *Agrobacterium*-mediated transformation in barley was developed by Tingay *et al*.^3^, using immature embryos of ‘Golden Promise’ as an explant. Other than this technique, pollen culture of ‘Igri’ is one of a few alternative efficient methods for barley transformation^4^. This situation poses a great challenge for complementation analysis in barley, because analysis of traits of interest is restricted to those alleles non-functional in ‘Golden Promise’ or ‘Igri’. Analysis of functional alleles generally requires development of a genetic substitution line of the target allele from the donor into ‘Golden Promise’ via multiple generations of back-crossing. Recently Yeo *et al*.^5^ developed a new barley line ‘Golden SusPtrit’ (SG062N) that combines the transformation ability of ‘Golden Promise’ with the high susceptibility to rust disease inherited from ‘SusPtrit’, to develop a line of barley amenable for functional studies of non-host and partial resistance to fungal rust. The enhanced rate of *Agrobacterium*-mediated transformation in this recombined line suggested the existence of genetic factors responsible for the transformation efficiency of ‘Golden Promise’. That study also compared the genomic differences between a transformation-susceptible line (SG062N) and an insusceptible line (SG133N) in the same double haploid (DH) mapping population, to predict the genomic regions responsible for *Agrobacterium*-mediated transformation in ‘Golden Promise’^5^.

Four quantitative trait loci (QTLs) responsible for regeneration of green shoots from calli in barely were previously identified in a ‘Steptoe’ × ‘Morex’ cross by Mano *et al*.^6^, then Bregitzer & Campbell^7^ identified eight QTLs using same population. In addition Mano & Komatsuda^8^ identified three QTLs for shoot regeneration and two QTLs for callus growth in other population, ‘Azumamugi’ × ‘Kanto Nakate Gold’, although no genetic factors for transformation ability itself were specifically investigated. In *Brassica oleracea*, Cogan *et al*.^9^ identified three QTLs involved in *Agrobacterium rhizogenes*-mediated root transformation using mature seed. The genes responsible have not yet been identified, although two of three loci are speculated to be paralogs of each other originated by genome duplication around these regions^10^. In *Solanum tuberosum,* El-Kharbotly *et al*.^11^ found a locus involved in transformation efficiency linked with the *R1* gene (resistance to *Phytophthora infestans*). In the case of rice, Nishimura *et al*.^12^ isolated a gene encoding ferredoxin-nitrate reductase (NiR) involved in a major QTL, promoter of shoot regeneration (PSR). In that case, the efficiency of regeneration in ‘Koshihikari’ was increased by introducing the intact genomic region or over-expressed cDNA of the *NiR* gene from ‘Kasalath’ in the callus derived from mature seed. Interestingly, Tyagi *et al*.^13^ reported that a QTL on chromosome 6H in barley, responsible for green shoot regeneration, was located close to the *HvNiR* locus (AK371794).

The efficiency of *Agrobacterium*-mediated transformation is heavily dependent on environmental and technical factors as well as the type and genetic makeup of the plant material under investigation. Technical factors include culture conditions, medium composition, type of *Agrobacterium* strain, and the specific binary vector and selection marker employed (reviewed by Cheng *et al*.^14^). The type of tissue used for transformation also plays an important role, as well as genetic factors that dictate interactions with *Agrobacterium* and subsequent development, including genes responsible for integration of T-DNA elements, cell division under selective conditions, and regeneration from callus^14^. Nam *et al*.^15^ reported that the efficiency of T-DNA integration into host genome depended on *Arabidopsis* ecotypes.

Recently, genome editing technologies, such as Trans Activator-Like Effector Nucleases (TALENs) and Clustered Regularly Interspaced Short Palindromic Repeats/CRISPR associated proteins 9 (CRISPR/Cas9) have been developed in plants. However, the utility of these technologies in barley is limited by the fact that transformation is necessary as the first step to introduce those nucleases in most plant species, as reported by Budhagatapalli *et al*.^16^ and Lawrenson *et al*.^17^

In this study, we mapped the genetic factors responsible for *Agrobacterium*-mediated transformation in ‘Golden Promise’ to develop a method that may be used for transformation, and thus, analysis of any barley gene. Instead of using genetic populations with fixed genotypes such as recombinant inbred lines (RILs) or double haploid (DH) lines, which requires phenotyping a large number of individuals under constant conditions, we used ‘Haruna Nijo’ (HN) × ‘Golden Promise’ (GP) derived F_2_ explants generated from individual immature embryos (Figure S1). We used the recombinant progeny of a cross GP × HN as a mapping population to identify the genetic factors that promote *Agrobacterium*-mediated transformation and demonstrated these genetic factors in an alternate cross between ‘Morex’ × GP. We propose that these genetic markers can be used to identify easily transformable progeny plants of the recalcitrant barley genotypes after crossing with GP, thus enabling gene/allele testing in the desirable background.

## Results

### Generation of transgenic HN × GP plants

We usually achieve approximately 10% efficiency of transformation using immature embryo of GP in our method. By the same experimental method, we didn’t obtain any transgenic plants from 261 immature embryo of HN. In addition, we tried to use immature embryos of BC_3_F_8_ recombinant chromosome substitute lines derived from a cross between GP and HN, with three rounds of backcrossing to HN as an explant for transformation, but did not obtain any transgenic plants from 4,661 immature embryos of these lines. Here, the F_2_ immature embryos derived from a cross between HN and GP (HN × GP) were inoculated with *Agrobacterium tumefaciens* strain AGL1 carrying a binary vector, pIG121-Hm, bearing genes for GUS (ß-glucuronidase) and HPT (Hygromycin PhosphoTransferase)^18^. From 3,013 immature embryos, 293 hygromycin-resistant calli were observed after selection on induction/selection medium. Green shoots were regenerated from 64 of those calli on regeneration medium. A total of 60 independent plants produced healthy roots on hygromycin-selective rooting medium, and were transferred to pots and grown in a controlled environment. DNA was isolated from leaves of the 60 candidate plants and used for subsequent genotyping and PCR analysis, which showed that all 60 candidate plants were PCR-positive for both *GUS* and *HPT* transgenes (Figure S2A,B). These data demonstrating incorporation of the transgenes suggested that all 60 candidates carried the genetic factors from GP that allowed *Agrobacterium*-mediated transformation.

### SNP marker analysis of transgenic HN × GP plants

**Figure 1:**
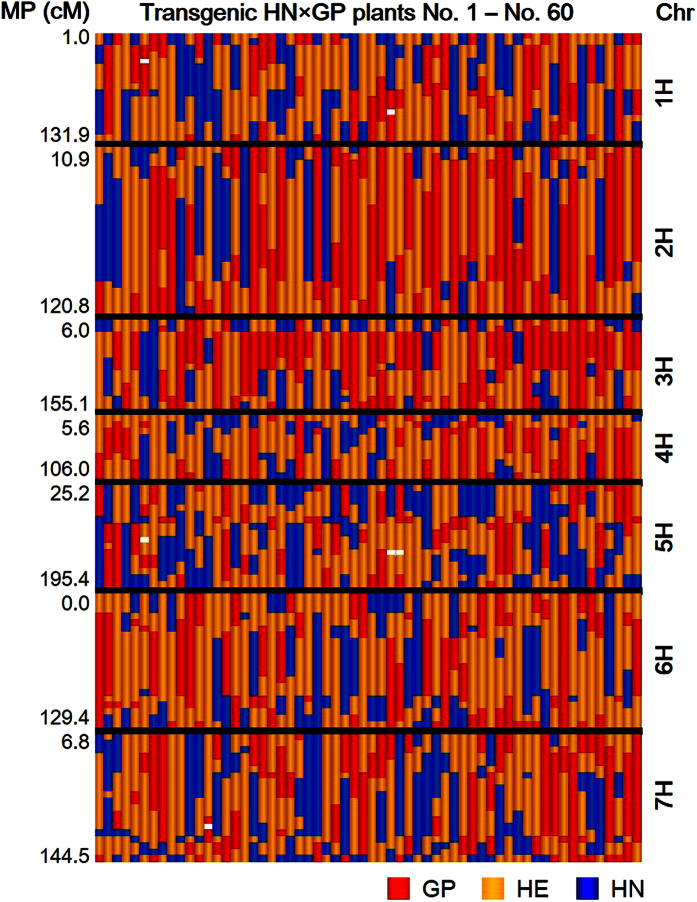
Graphical overview of genotypes in transgenic HN × GP plants. Genotyping of transgenic HN × GP plants from 1–60 (from left to right) was performed using the 384-SNP platform Illumina GoldenGate^®^ assay. One hundred and twenty-four SNP markers are shown according to marker position (MP) in each chromosome (Chr) based on the consensus genetic distance of barley 1,19. Alleles from Haruna Nijo (HN), heterozygous regions (HE), and alleles from Golden Promise (GP) are shown as blue, yellow and red, respectively. The number on left show the end MP (cM) in each Chr.

The genotypes of the 60 transgenic HN × GP plants were determined using a 124-marker subset of the 384-SNP Illumina GoldenGate^®^ platform from the barley oligonucleotide pooled assay 1 (BOPA1)^19^. The genetic positions of all markers in this assay are available in a consensus genetic map^19^. The genotyping data for the transgenic HN × GP plants is presented in Table S1 and the genotype based on marker order in each chromosome is presented in Fig. 1. Each transgenic HN × GP plant had a unique genotype, indicating that these 60 plants resulted from independent transformation events.

### Allele frequency and analysis of segregation distortion

**Figure 2:**
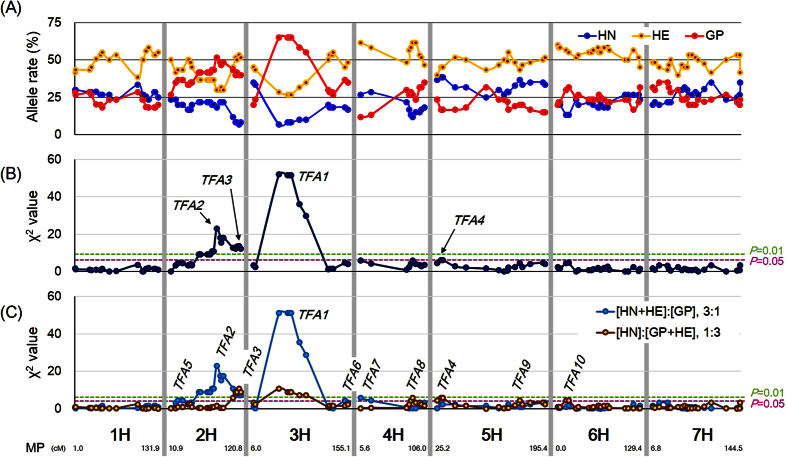
Allele and segregation analysis of SNP markers in transgenic HN × GP plants. Allele segregation (**A**) and Chi-square (χ^2^) values (**B,C**) were calculated by genotyping 124 SNP markers in transgenic HN × GP plants. SNP markers are shown from short arm (left) to long arm (right) in each chromosome according to the order and genetic distance of markers in genome information of barley^1^,^19^. The number (cM) on bottom shows the end marker position (MP) in each chromosome. (**A**) Blue, yellow, and red line plots show the percentage of ‘Haruna Nijo’ (HN), heterozygous (HE) and Golden Promise (GP) alleles for each SNP marker. (**B**) Dark blue line plots show χ^2^ values calculated using 1:2:1 as the expected segregation ratio. Lime green and magenta dotted lines indicate statistical significance at the 1% and 5% levels, respectively (df = 2). (**C**) Light blue and brown line plots show χ^2^ values calculated using 3:1 as the expected segregation ratios for [HN + HE]:[GP] and [GP + HE]:[HN], respectively. Lime green and magenta dotted lines indicate statistical significance at the 1% and 5% levels, respectively (df = 1). *TFA*s (*TFA1-10*) indicate the loci showing segregation distortion.

The allele frequency was calculated based on the genotyping data and mapped to the corresponding chromosomes (Fig. 2A). The Mendelian law of independent assortment predicts that the alleles homozygous for GP, heterozygous (HE), and homozygous for HN should segregate in a 1:2:1 ratio. The GP allele displayed a frequency of over 40% in the regions between markers *2580-1456* (position: 55.0 cM) and *3256-1196* (120.8 cM) in chromosome 2H and between *4105-1417* (46.3 cM) and *8020-87* (88.8 cM) in 3H (Fig. 2A and Table S1).

Chi-square (χ^2^) test was used to identity segregation distortions caused by selection for transformation and regeneration (Fig. 2B,C). These χ^2^ values for the expected Mendelian ratio (HN:HE:GP = 1:2:1) were plotted with the marker positions on the barley genome (Fig. 2B). The maximum χ^2^ value was 52.1, with marker *4105-1417* on chromosome 3H, while the surrounding region between *4105-1417* (46.3 cM) and *8020-87* (88.8 cM) showed statistically significant segregation distortion (df = 2, *p* < 0.01). We named this region as *TFA1 (Transformation Amenability 1*). Another region with significant segregation distortion was observed on chromosome 2H between *2580-1456* (55.0 cM) and *3256-1196* (120.8 cM) (df = 2, *p* < 0.01). This region included two peaks with markers *6117-1507* (82.8 cM, χ^2^ = 22.9) and *7576-818* (117.9 cM, χ^2^ = 13.6), and these loci were named *TFA2* and *TFA3*, respectively. An increased frequency of GP alleles at these regions suggested that a factor for transformation efficiency in GP was located there. Interestingly, an increased frequency of the HN allele was observed in a region of 5H between *8377-1022* (30.99 cM) and *4684-775* (34.25 cM), and was named as *TFA4*. The χ^2^ values of loci with significant segregation distortion are summarized in Table 1.

To examine the direction of dominance of the identified loci, the dominance segregation ratio (3:1) was used for χ^2^ tests (Fig. 2C). Using the assumption that the GP allele was functionally recessive ([HN + HE]:[GP] = 3:1), the regions or markers showing significant distortion were observed between markers *1865-396* (21.6 cM) and *7032-201* (29.2 cM) in 2H, at *7818-967* (150.4 cM) in 3H and between *ABC14522-1-8-350* (5.5 cM) and *2055-947* (21.6 cM) in 4H, in addition to *TFA1, TFA2* and *TFA3*. These additional loci were named as *TFA5, TFA6* and *TFA7*, respectively (light blue line in Fig. 2C and Table 1). Meanwhile, using the assumption that the GP allele was dominant ([GP + HE]:[HN] = 3:1), loci between *4986-1214* (84.3 cM) and *4564-604* (87.5 cM) in 4H, at *ConsensusGBS0451-1* (155.1 cM) in 5H and between *ConsensusGBS0346-1* (12.5 cM) and *1769-545* (17.0 cM) in 6H were identified, alongside *TFA1, TFA3* and *TFA4*. These new loci were named as *TFA8, TFA9* and *TFA10*, respectively (brown line in Fig. 2C and Table 1). Interestingly, the χ^2^ value for *TFA2* was not significant under the GP-dominant condition, although it showed significant segregation distortion under [HN + HE]:[GP] = 3:1.

### Cloning and genotyping of the *HvNiR* gene

**Figure 3:**
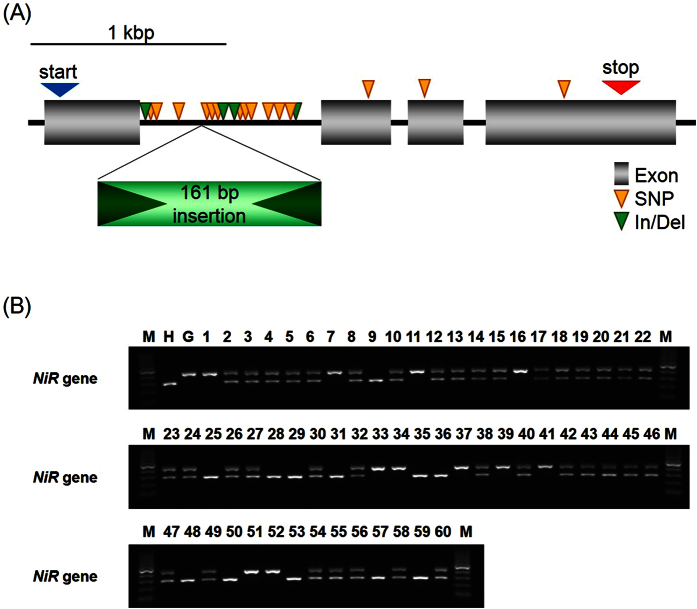
Genic structure and allele of the *HvNiR* gene in transgenic HN × GP plants. (**A**) The structure of *HvNiR* genic region including introns, start and stop codons. Yellow and green wedges show SNPs and Insertions/Deletions, respectively, between ‘Haruna Nijo’ and ‘Golden Promise’. A 161 bp insertion of retrotransposon-like sequence was present in the first intron of *HvNiR* in ‘Golden Promise’. (**B**) Genotyping of transgenic HN × GP plants was performed using *HvNiR*-specific primers. The expected sizes of PCR fragments were 280 bp and 417 bp in alleles of ‘Haruna Nijo’ (H) and ‘Golden Promise’ (G), respectively. M, 100 bp ladder marker; 1–60, individual transgenic HN × GP plants. Original gel image is available from Supplementary Information.

To examine whether the barley ortholog of rice *NiR* played a role in transformation efficiency, we isolated the *NiR* ortholog from HN and GP for comparison between these two cultivars. The putative *HvNiR* gene, including predicted start and stop codons, was 2,965 bp in HN and 3,097 bp in GP. Analysis of the *HvNiR* genes in HN and GP revealed 4 exons and 3 introns, and a comparison of the DNA sequence between HN and GP yielded 14 SNPs in introns and 3 synonymous SNPs in exons (Fig. 3A). In addition, the HN allele had a 7-bp and a 23-bp insertion in the first intron, while the GP allele had a 161-bp insertion homologous to a retrotransposon-like sequence in the first intron (Fig. 3A). Despite these differences, the predicted amino acid sequences for *HvNiR* in HN and GP were identical.

To prove whether the insertions in the first intron of *HvNiR* might be related to transformation efficiency, we designed a codominant sequence-tagged-site (STS) marker to amplify the region spanning the GP-specific 161-bp insertion for genotyping, and performed an STS analysis using DNA from the 60 transgenic HN × GP plants. Analysis of the alleles revealed a segregation ratio of 12:37:11 (HN:HE:GP), which fits a monofactorial Mendelian ratio (Fig. 3B, χ^2^ = 3.3 at 1:2:1 ratio) in the absence of segregation distortion.

### Genome-wide locus-locus interactions

**Figure 4:**
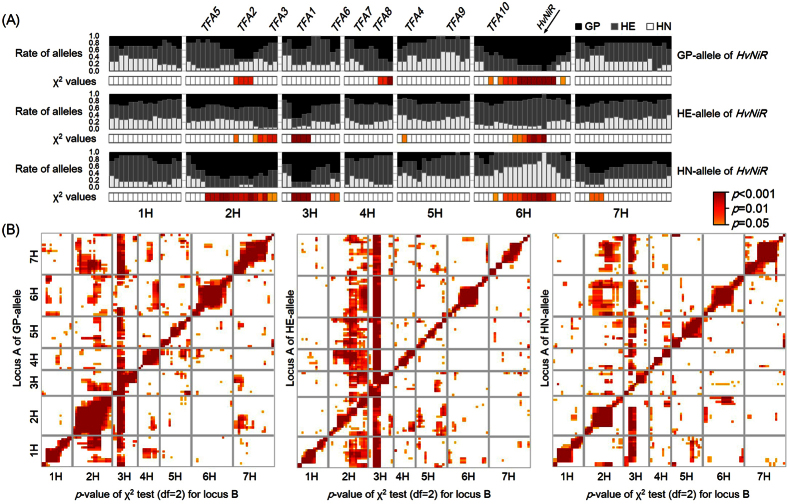
Analysis of locus-locus interactions in transgenic HN × GP plants. (**A**) Percentages of ‘Golden Promise’ (GP, black)-, heterozygous (HE, dark gray)- and ‘Haruna Nijo’ (HN, light gray) alleles were calculated among transgenic HN × GP plants having GP (top), HE (middle) and HN (bottom) alleles of *HvNiR* and shown as band charts. One hundred and ten SNP markers and the *HvNiR* gene marker are shown from short arm (left) to long arm (right) of the chromosomes. *TFA*s (*TFA1-10*) indicate the loci showing segregation distortion in Fig. 2. The heat maps based on Chi-square (χ^2^) tests of the markers (df = 2) are shown under each band chart. (**B**) Heat maps were made based on *p*-values of χ^2^ tests (df = 2) representing significance of segregation distortions in transgenic HN × GP plants. The markers (locus A) were grouped into GP (left panel), HE (middle panel) and HN (right panel) alleles and ordered on vertical axis from short arm (bottom) to long arm (top) in each chromosome. Then, χ^2^ tests (df = 2) were performed for all other markers (locus B) ordered on horizontal axis from short arm (left) to long arm (right) in each chromosome; the *p*-values representing degree of significance are indicated by color grade.

Normally, alleles corresponding to opposing phenotype data (e.g., resistant vs. sensitive to a given stress) are used for locus-locus interaction analysis. Since we used transgenic plants to map transformation efficiency, i.e. most loci have transformation-amenable alleles, there is selection bias in our mapping populations, and the standard software does not serve to detect locus-locus interactions. To analyze locus-locus interaction in the transgenic HN × GP plants, we first grouped genotypes according to the allele type of a single marker, we then calculated the segregation ratio and χ^2^ value of other markers relative to the first marker. For instance, using the *HvNiR* locus, the HN × GP plants were placed into groups of 11 plants for GP, 37 plants for HE and 12 plants for HN based on the alleles at *HvNiR* (top, middle and bottom panels in Fig. 4A, respectively), we then compared the ratio (band chart) and *p*-values of χ^2^ test (heat map, df = 2) of alleles in each of the other loci. Because *HvNiR* was located on the long arm of chromosome 6H, the frequency of alleles of this region matched the frequency of *HvNiR*. On chromosome 2H, distortion favoring the GP allele was observed around *TFA2* in GP- and HN-type *HvNiR* groups but not in the HE-type group (Fig. 4A). Also around *TFA3*, GP and HE alleles were dominant in HN- and HE-type *HvNiR* groups, but not in the GP-type group. Interestingly, there was no segregation distortion around *TFA1* among the GP-type *HvNiR* plants. Additional segregation distortion was also observed on long arm of chromosome 4H in GP-type *HvNiR* plants (Fig. 4A).

Using above method, χ^2^ tests for all of the non-redundant markers (111 in total) were performed in the transgenic HN × GP plants. First, we counted the number of the allele combinations between one locus (locus A) and another (locus B) (e.g. GP-HN for locus A-B), then calculated χ^2^ values (df = 2) for segregation of locus B among each type of allele on locus A. Fig. 4B shows the heat maps of *p*-values representing significant segregation distortion of locus B on the horizontal axis against locus A on the vertical axis, which are separated into the cases of GP (left panel), HE (middle panel) and HN alleles (right panel). A segregation distortion was observed around *TFA1* as locus B against almost all of the markers, except for the region in chromosome 6H including *HvNiR* for which the HN × GP plants had the GP allele (left panel in Fig. 4B). The plants having the GP allele in this region of 6H showed segregation distortion in the long arm of 4H as well as at *TFA2* (left panel in Fig. 4B). Apart from these loci, the major segregation distortions were observed at 1H-4H, 2H-7H, 3H-7H, 4H-5H, 7H-2H and 7H-4H with GP-allele markers as locus A (left panel in Fig. 4B). With HN-allele markers as locus A in HN × GP plants, segregation distortions were observed at 1H-2H, 1H-5H, 2H-6H, 3H-2H, 6H-2H and 7H-2H in addition to ones with *TFA1* (right panel in Fig. 4B). Particularly the plants with the HN alleles in 6H and 7H as locus A showed widespread segregation distortion in 2H as locus B.

### Recursive test for genetic factors of transformation amenability by an alternative cross

**Figure 5:**
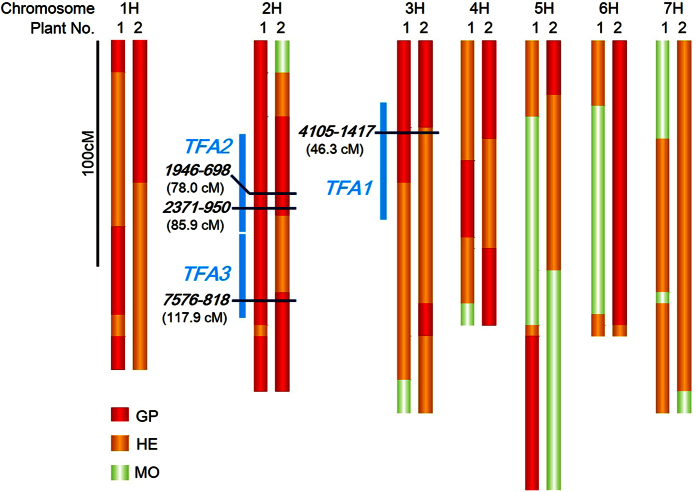
Graphical genotypes in transgenic MO × GP plants. Genotyping of two transgenic MO × GP plants, No. 1 and No. 2, was performed by 384-SNP platform Illumina GoldenGate^®^ assay. One hundred and seventy-eight SNPs markers were positioned from short arm (above) to long arm (bottom) of chromosomes according to the order of marker position in the consensus genetic distance of barley 1,19. Alleles of Morex (MO), hetero (HE) and Golden Promise (GP) were shown as green, yellow and red, respectively. Light blue bars and markers with position (cM) indicate the regions and nearest marker of *TFL1, TFL2* and *TFL*3, respectively.

To demonstrate the *TFAs*, particularly *TFA1, TFA2* and *TFA3*, as important genetic factors for transformation in barley, we performed *Agrobacterium*-mediated transformation on F_2_ immature embryos derived from a cross between ‘Morex’ (MO) and ‘Golden Promise’ (MO × GP). Two independent transgenic MO × GP plants from 1,722 immature embryos were obtained and genotyped using the GoldenGate^®^ platform. The genotypes of these transgenic MO × GP plants and the locations of *TFA1, TFA2* and *TFA3* are illustrated in Fig. 5. Most of chromosomes 1H, 2H, 3H and 4H corresponded to the homozygous GP or heterozygous genotype in both plants; however, large regions in chromosomes 5H, 6H and 7H corresponded to the MO genotype in one or both plants. At *TFA1*, one marker *4105-1417* detected both GP and heterozygous alleles in the MO × GP transgenic plants. Additionally, the markers *1946-698* (78.0 cM) and *2371-950* (85.9 cM), on *TFA2*, and marker *7576-818* (117.9 cM) in *TFA3*, all detected the GP allele. These results confirm that GP alleles of *TFA1, TFA2* and *TFA3* are effective for transformation in an alternative haplotype of barley.

## Discussion

The success of genetic transformation in plants depends on various factors. The immature embryo of ‘Golden Promise’ (GP) is the most reliable material reported for *Agrobacterium*-mediated transformation in barley^20^. Previously, we attempted to use BC_3_F_8_ recombinant chromosome substitution lines for transformation assays to identify possible genetic factors regulating *Agrobacterium*-mediated transformation in barley. However, we did not obtain a single transgenic plant from 4,661 immature embryos, likely due to insufficient factors in the genetic material favoring transformation. In this study, to increase the chance of generating a population of transformants for further study, we employed a different approach, utilizing the F_2_ generation of immature embryos derived from a cross between ‘Haruna Nijo’ (HN) and GP as the material for *Agrobacterium*-mediated transformation. With this new approach, we successfully obtained 60 transgenic barley plants (HN × GP) from 3,013 F_2_ individuals. Utilizing these 60 transgenic plants for genome-wide analysis, we identified regions of the genome that display significant segregation distortion of alleles from the expected 1:2:1 ratio. We found major regions of segregation distortion in chromosomes 2H and 3H, which we named *TFA1, TFA2* and *TFA3*, where the GP allele was highly over represented in the transformants (Fig. 2B). The relatively higher rate of transformation in heterozygote individuals also suggests that some the possible factors regulating transformation efficiency exhibit dominance (Fig. 2A). Importantly, the GP alleles of these *TFAs* were conserved in two transgenic plants derived from an alternative cross, MO × GP (Fig. 5). This report is the first identifying the loci underlying transformation amenability in barley by measuring segregation distortion of alleles in the F_2_ population. We also confirmed that organelle genomes likely do not play a role in transformation amenability, since all HN × GP and MO × GP transgenic plants originated from a cross using GP as the pollen parent. To confirm that the distortion was not caused by a reproductive barrier between HN and GP, we performed a SNP analysis using the F_10_ generation of RILs derived from the same cross (Table S2). We found that three genomic regions, on 2H, 3H and 5H, in the RILs were distorted; however, these regions were in different positions from *TFAs* and were caused by overaccumulation of the HN allele, rather than the GP allele (Table S2). The distortions observed in the RILs could be due local adaptation of the HN genotype to the field in Kurashiki, Japan during generation advancement of the population. Overall, however, these findings indicate that the distortions that led to the identification of the *TFAs* were not caused by abnormal segregation between GP and HN genotypes due to a reproductive barrier.

Several genetic factors that affected the efficiency of *Agrobacterium-*mediated transformation have been documented, for example, factors responsible for interaction with *Agrobacterium*, growth rate of calli, as well as regeneration ability^14^,^20^. Rikiishi *et al*.^21^ reported that barley cultivar ‘Lenins’ has higher regeneration ability compared to some other cultivars, but that trait alone is not enough for efficient transformation. Yeo *et al*.^5^ suggested that a region close to *TFA2* on chromosome 2H, of the line SG062N, may affect transformation efficiency. The locations of *TFA6* and *TFA8* also correspond with regions previously reported by Yeo *et al*.^5^ Mano *et al*.^6^, Mano & Komatsuda^8^ and Bregitzer & Campbell^7^, reported that QTLs promoting green shoot regeneration from callus in barley were observed on chromosomes 1H, 2H (*Qsr1*), 3H (*Qsr2*), 5H (*Qsr4*), 6H (*Qsr3*) and 7H. Because *TFA2* was mapped close to *Qsr1*, this locus could be related to regeneration as well as increased transformation efficiency. *TFA3* was mapped adjacent to *Qcg1*, and the function of *TFA3* might be involved in propagation ability of calli under hygromycin selection. We found that *TFA1* was mapped between *Qsr2* and *Qcg2*. Thus, we speculate that *TFA1* may also be involved in regeneration or growth efficiency of calli. In dicot plants, Nam *et al*.^15^ mentioned that the efficiency of integration of T-DNA into *Arabidopsis* genome was dependent on its genotype/ecotype and controlled by a single major locus. Also Sparrow *et al*.^22^,^23^ reported that the genetic analysis of *Agrobacterium* susceptibility using a doubled haploid mapping population of *Brassica oleracea*. They found the genetic segregation of efficiency of GUS expression implying the integration of T-DNA, and the related QTL for crown gall formation. While the exact function of *TFAs* in barley is still unknown, it is possible that some of them play similar roles in responding to *Agrobacterium* including integration of T-DNA into barley genome, cell propagation and tissue regeneration. Interestingly, *TFA2* and *TFA3* showed different patterns of segregation distortion based on 3:1 segregation ratios (Table 1, Fig. 3C). We speculate that GP allele function may be recessive at *TFA2* but dominant at *TFA3* for transformation amenability. Dominance of the HN allele was observed at *TFA4, TFA7* and *TFA9*. This result indicated that there could be negative factors for transformation in GP at these loci, or possibly positive factors in HN, in which regeneration levels are moderate^24^,^25^. *TFA4* was mapped near the locus *ari-e*.GP, which is a marker of the semi-dwarf phenotype due gibberellin insensitivity^26^,^27^. Therefore, this locus might control the growth and development of callus tissue as well as plant biomass. Number of transgenic plants in all combinations of alleles at *TFA4, TFA7* and *TFA9* and Chi-square values among genotypes were shown in Figure S3 and Table S3, respectively. Every single locus segregation in *TFA4, TFA7* and *TFA9* showed significant difference at least one of the co-dominant or dominant segregations (Fig. 2 and Table S3). The allelic combination of *TFA4* and *TFA9* also showed statistical significance, indicating that the combination of HN-alleles of *TFA4* and *TFA9* might have positive epistatic effect for transformation amenability. The result encourage us that the substitution of HN allele of *TFA4* and *TFA9*, from GP allele may develop more efficient transformation amenable lines. As well, the regeneration level of MO was severely low in our condition^25^. If we perform further analysis using MO × GP, we may find another factors which Morex has as negative for regeneration. This might be reason why we obtained only two transgenic plants from 1722 immature embryos of MO × GP. A gene encoding ferredoxin-nitrate reductase (NiR) contributes to increased regeneration and transformation efficiency in rice^12^. Tyagi *et al*.^13^ reported that an e-QTL in barley for green shoot regeneration was located on the marker HVSMEI0013e16r2, near the *NiR* gene on chromosome 6H. To study the effect of *NiR* on *Agrobacterium*-mediated transformation in barley, we isolated the barley orthologue of *NiR (HvNiR*) in GP, which contained 3 introns (Fig. 3A). The HN *HvNiR* allele differed from the GP allele by 3 synonymous SNPs in exons, and 12 SNPs and 5 insertion/deletions in the first intron. We found that the segregation of alleles on *HvNiR* followed the 1:2:1 Mendelian ratio in transgenic HN × GP plants, as would be expected if the alleles were independently assorted (Fig. 3B). We further analyzed locus-locus interaction between *HvNiR* and *TFAs* (Fig. 4A,B) and found that the plants with the GP allele at *HvNiR* showed segregation distortion in *TFA2* but not in *TFA1* and *TFA3*. In the case of the HN allele at *HvNiR*, plants showed segregation distortion on *TFA1* as well as the region including *TFA2* and *TFA3*. We speculate that *HvNiR* may complement the function of *TFA1, TFA2* and *TFA3*, although *HvNiR* itself did not show a significant χ^2^ value for distortion (Fig. 4A,B). These results suggest that *HvNiR* may contribute to transformation efficiency in barley, as well as in rice.

Currently, if researchers want to carry functional analyses of genes for which GP does not carry a suitable haplotype, they generally develop substitution barley lines backcrossed with GP for transformation. For instance, Deng *et al*.^28^ made BC_3_F_2_ barley lines carrying a *VRN1*-*HA (VRN1, vernalization 1*; *HA*, haemagglutinin epitope tag) transgene for analysis to discover the target genes of *VRN1*, which encodes a MADS box transcription factor. To do this, the authors first had to generate BC_2_F_1_ plants of winter-type GP introgressed with vernalization-requiring genome sets by backcrossing twice before crossing with the transgenic GP. Such studies require a substantial amount of time to develop the plants necessary to conduct further analysis.

**Figure 6:**
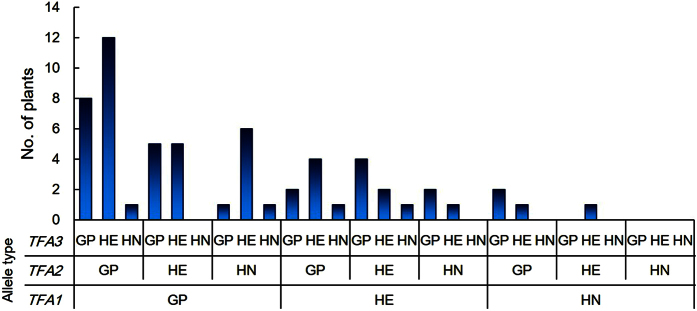
Frequency of transgenic HN × GP plants classified by genotype at *TFA1, TFA2* and *TFA3*. Transgenic HN × GP plants were grouped by the haplotype of *TFA1, TFA2* and *TFA3* and counted.

**Figure 7:**
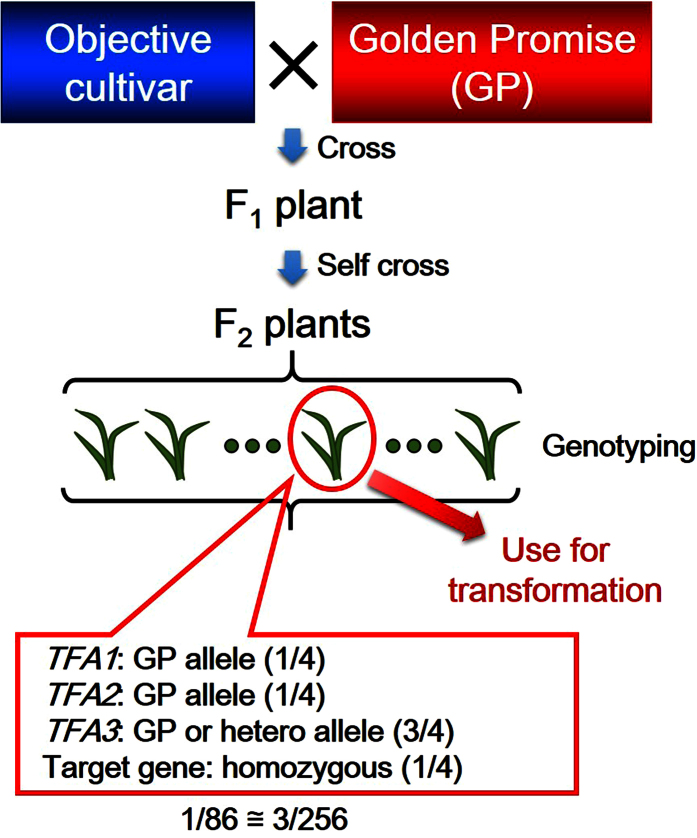
The schematic overview of proposed *TFA*-based universal complementation system in barley. For complementation tests of target genes, F_2_ plants would be generated from a cross between the objective cultivar with the target gene and cv. ‘Golden Promise’. Among the F2 plants, genotyping would be carried out to identify plants with the GP allele at *TFA1* and *TFA2*, and GP/heterozygous at *TFA3*, as well as homozygous for the target gene. One plant fitted to the purpose is predicted to be found among approximately each 86 F_2_ plants.

The identification and characterization of the *TFAs* should enable the scientific community to conduct complementation experiments relatively rapidly in any haplotype of barley, which has thus far been limited to the alleles of transformation-efficient cultivars such as ‘Golden Promise’ and ‘Igri’. We have demonstrated the ability to conduct transformation from any haplotype using the F_2_ generation derived from a cross between the haplotype of interest and GP (Fig. 5). Figure 6 shows the number of transgenic HN × GP plants with various allele combinations in *TFA1, TFA2* and *TFA3*. Among 60 transgenic HN × GP plants, 20 plants had only the GP alleles in *TFA1* and *TFA2,* and the GP allele or heterozygosity in *TFA3*. While our study did not identify the specific genes necessary for barley transformation, we have demonstrated that these specific allele combinations at the identified *TFA* regions increase the rate of successful transformation. We hereby propose our method as a possible universal complementation system for the barely research community. This *TFA*-based method involves genotyping F_2_ plants to collect the necessary alleles of *TFAs* segregating in the population as well as the target allele to be analyzed (Fig. 7). Importantly, the desired combination should be fairly common, with approximately one out of every 86 F_2_ plants harboring the correct sets of *TFA* alleles as well as the target allele. In our estimation, it takes in total approximately 13 months minimum to obtain transgenic T_0_ plants from the first cross in our system; 1 month to harvest matured F_1_ seed; 5 months to harvest F_2_ seeds on the F_1_ plant; 4 months to perform genotyping and to harvest immature embryo for transformation; 3 months to generate transgenic T_0_ plants.

In conclusion, in this study, we generated transgenic barley plants from the progeny of a cross between ‘Haruna Nijo’ and ‘Golden Promise’. Genotypic analysis of the HN × GP transgenic plants, revealed 3 significant and 7 suggestive QTLs represented by segregation distortion regions, which we named *TFA*s for *Transformation Amenability*. Because these transgenic plants were generated via successful *Agrobacterium*-mediated transformation events, we believe that these segregation distortions identified regions in the genome containing genetic factors that are necessary for or enhance successful transformation. To demonstrate the role of *TFA*s in producing transgenic plants, we also showed the presence of the *TFA*s in transgenic plants generated from a different cross (MO × GP). Inter-locus interactions were observed for several genomic regions, particularly between *TFAs* and chromosome 6H including the *HvNiR* gene. We anticipate utilization of these *TFAs* to enable more efficient analysis of barley genomes, genes, and alleles, not only in ‘Golden Promise’, but in all barley cultivars and hybrid progenies.

## Methods

### Plant materials

The hybrid F_1_ barley plants (HN × GP or MO × GP) were derived from ‘Haruna Nijo’ (HN) or ‘Morex’ (MO), respectively, as a seed parent and ‘Golden Promise’ (GP) as a pollen parent. Plants were grown under 16 hours of daylight per 24 hour cycle and 15 °C/13 °C (day/night). The caryopses were harvested for isolation of immature embryos approximately 14 days after pollination.

### *Agrobacterium-*mediated transformation

Transformation in barley was performed following the protocol of Hensel *et al*.^29^ The dissected immature embryos were soaked in 100 mg/l acetosyringone solution at 43 °C for 4 minutes before inoculation with *Agrobacterium* following Hiei *et al*.^30^ and Zheng *et al*.^31^ *Agrobacterium tumefaciens* strain AGL1 carrying the binary vector pIG121-Hm^18^ bearing the *ß-glucuronidase (GUS*) and *hygromycin phosphotransferase (HPT*) genes was used for inoculation.

### DNA analysis by PCR

Genomic DNA was extracted from the leaves of barley plants using the DNeasy Plant mini kit (QIAGEN, Germany). PCR amplification was performed with the following program: initial denaturation at 95 °C for 2 min, 28 cycles of denaturation at 95 °C for 30 sec, annealing at 55 °C (for the *GUS* gene) or 60 °C (for *HPT*) for 30 sec, and extension at 72 °C for 30 sec, with a final extension at 72 °C for 10 min. The 10-μl reaction mixture for PCR included 50 ng genomic DNAs as a template, 1 × GoTaq^®^ Green Master Mix (Promega, USA) and 0.5 μM each specific primer, as follows: gus4 (5′-AACAGTTCCTGATTAACCACAAACC-3′) and gus5 (5′-GCCAGAAGTTCTTTTTCCAGTACC-3′) for the *GUS* gene, and hph1 (5′-GCTGGGGCGTCGGTTTCCACTATCGG-3′) and hph2 (5′-CGCATAACAGCGGTCATTGACTGGAGC-3′) for the *HPT* gene.

### Cloning the *HvNiR* gene

To clone the *HvNiR* gene (Acc. No. LC097010 and LC097011 and for GP and HN, respectively), PCR amplification was performed with following program: initial denaturation at 98 °C for 2 min, 30 cycles of denaturation at 98 °C for 10 sec, primer annealing at 55 °C for 15 sec, and extension at 72 °C for 150 sec, with a final extension at 72 °C for 10 min. The 30-μl reaction mixture for PCR included 50 ng genomic DNA from ‘Haruna Nijo’ or ‘Golden Promise’ as a template, 1 × PrimeSTAR^®^ Max DNA Polymerase (TAKARA, Japan) and 0.5 μM specific primers HvNiR-F1 (5′-AACCACAAGCAGCATCCATG-3′) and HvNiR-R1 (5′-GAGATCATCAGGAGAAGGAG-3′). PCR products were cloned into pCR^TM^4-TOPO^®^ (Invitrogen, USA) and sequenced according to manufacturer’s protocol using the TOPO^®^ TA Cloning^®^ Kit for Sequencing (Invitrogen, USA).

### Genotyping

The 384-SNP platform of Illumina GoldenGate^®^ oligonucleotide pool assays^19^ was employed for genotyping all transgenic plants generated in this study using genomic DNA extracted with the DNeasy Plant mini kit, according to the manufacturer’s protocol (Illumina, USA).

For genotyping with the *HvNiR* gene marker, PCR amplification was performed with a modified touchdown PCR program^32^: initial denaturation at 95 °C for 2 min, 10 cycles of melting at 95 °C for 30 sec, annealing at 65 °C decreased 1 °C/cycle for 20 sec, and extension at 72 °C for 30 sec, followed by 20 cycles of denaturation at 95 °C for 10 sec, annealing at 55 °C for 20 sec and extension at 72 °C for 30 sec, with a final extension at 72 °C for 10 min. The 10-μl reaction mixture for PCR included 50 ng genomic DNA as template, 1 × GoTaq^®^ Green Master Mix and 0.5 μM specific primers HvNiR-GPspeF (5′-CCCGCATGCATATCCCATAG-3′) and HvNiR-GPspeR (5′-CCCGGACTAGTCCAAGATAC-3′). PCR products were analyzed by electrophoresis with 1.5% agarose gels (Wako, Japan) in TBE running buffer.

### Detection of distorted segregation

Chi-square (χ^2^) tests were performed to identify genome regions displaying significant segregation distortion using the expected Mendelian ratio of 1:2:1 (df = 2) for the null hypothesis as well as assumed dominant ratios of 3:1 or 1:3 (df = 1). Microsoft^®^ Excel^®^ was used for all calculations, charts, and heat maps.

## Additional Information

**How to cite this article**: Hisano, H. and Sato, K. Genomic regions responsible for amenability to *Agrobacterium*-mediated transformation in barley. *Sci. Rep.* **6**, 37505; doi: 10.1038/srep37505 (2016).

**Publisher’s note:** Springer Nature remains neutral with regard to jurisdictional claims in published maps and institutional affiliations.

## Supplementary Material

Supplementary Figures and Tables

## Figures and Tables

**Table 1:** Summary of detected *TFAs.*

Locus	Chr No.	Interval (cM)	Peak position (cM)	Peak Marker	Allele frequency HN:HE:GP	χ^2^ value		
						1:2:1^a^	3:1^b^ ([HN + HE]:GP)	3:1^b^ ([GP + HE]:HN)
*TFA1*	3H	46.31–88.82	46.3	*4105-1417*	4:17:39	52.10**	51.20**	10.76**
*TFA2*	2H	54.95–90.10	82.6	*6117-1507*	11:18:31	22.93**	22.76**	1.42
*TFA3*	2H	90.10–120.80	117.9	*7576-818*	4:32:24	13.60**	7.20**	10.76**
*TFA4*	5H	25.23–34.25	31.0	*8377-1022*	23:27:10	6.23*	2.22	5.69*
			34.3	*4684-775*				
*TFA5*	2H	21.61–29.15	21.6	*1865-396*	12:26:22	4.4	4.36*	0.8
			28.4	*7747-1056*				
			29.2	*7032-201*				
*TFA6*	3H	—	150.4	*7818-967*	11:27:22	4.63	4.36*	1.42
*TFA7*	4H	5.55–21.61	5.6	*ABC14522-1-8-350*	16:37:07	5.97	5.69*	0.09
*TFA8*	4H	84.30–87.49	87.5	*4564-604*	7:37:16	5.97	0.09	5.69*
*TFA9*	5H	—	155.1	*ConsensusGBS0451-1*	22:26:12	4.4	0.8	4.36*
*TFA10*	6H	12.54–16.97	17.0	*1769-545*	8:33:19	4.63	1.42	4.36*

^a,b^Asterisks indicate statistical significance by Chi-square test (^a^df = 2, ^b^df = 1, ^**^p < 0.05, ^**^p < 0.01).
