# Supplementary material for: Genomic regions responsible for amenability to Agrobacterium-mediated transformation in barley
Source: Sci Rep. 2016 Nov 22;6:37505. doi: 10.1038/srep37505 (PMC5118740; doi:10.1038/srep37505)
Supplement: Supplementary Figures and Tables [file srep37505-s1.pdf]

Supplementary data

Title: Genomic regions responsible for amenability to *Agrobacterium*-mediated transformation in barley

Authors: Hiroshi Hisano and Kazuhiro Sato

Supplementary figure legends

Figure S1. Schematic overview of plant materials and methods in this study.
The F_2 generation of immature embryo derived from a cross between barley cvs. 'Haruna Nijo' and 'Golden Promise' was used for *Agrobacterium*-mediated transformation. The resultant transgenic barley plants were genotyped using SNP markers.

Figure S2. PCR analysis of 60 independent transgenic HN×GP plants.
PCR was performed using genomic DNA of transgenic HN×GP plants with specific primers for (A) *beta-glucuronidase* (*GUS*) and (B) *hygromycin phosphotransferase* (*HPT*). The expected sizes of PCR fragments were (A) 636 bp and (B) 375 bp, respectively. G, non-transgenic GP; H, non-transgenic HN; M, 100 bp ladder marker; P, plasmid positive control (pIG121-Hm); 1-60, Individual transgenic HN×GP plants.

Figure S3. Frequency of transgenic HN×GP plants classified by allele type at *TFA4*, *TFA7* and *TFA9*.
Transgenic HN×GP plants were grouped by the haplotype of *TFA4*, *TFA7* and *TFA9* and counted.

Figure S1

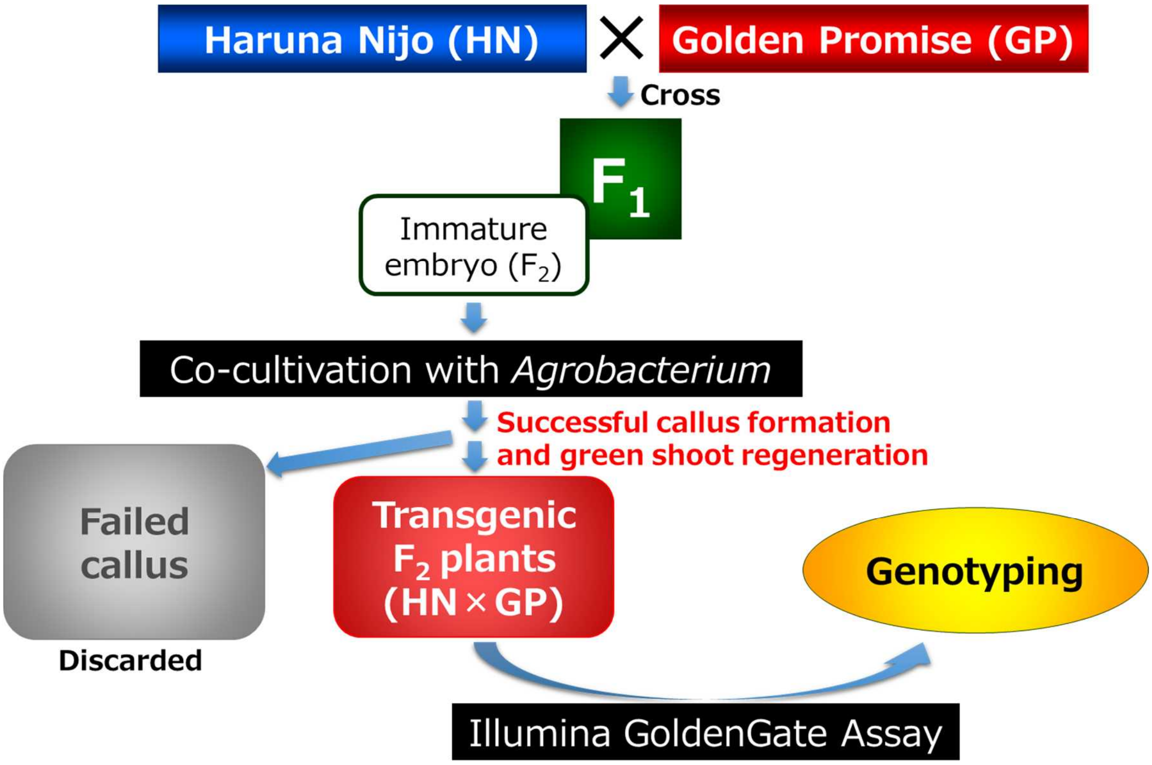

Haruna Nijo (HN)
Golden Promise (GP)
Cross
F1
Immature embryo (F2)
Co-cultivation with *Agrobacterium*
Successful callus formation
and green shoot regeneration
Failed callus
Discarded
Transgenic F2 plants (HN × GP)
Genotyping
Illumina GoldenGate Assay

Figure S2

(A)

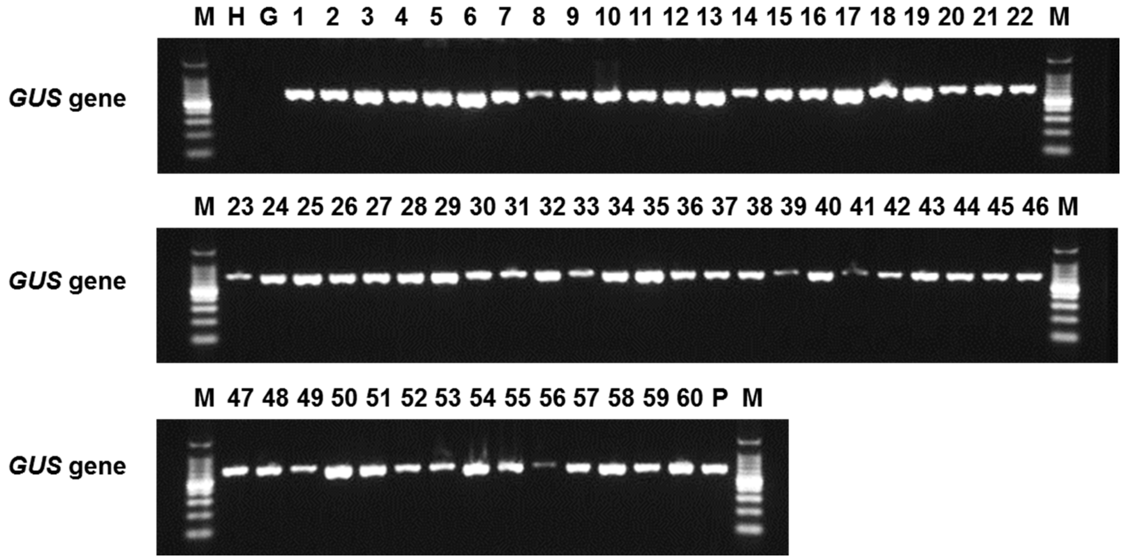
M H G 1 2 3 4 5 6 7 8 9 10 11 12 13 14 15 16 17 18 19 20 21 22 M
GUS gene
M 23 24 25 26 27 28 29 30 31 32 33 34 35 36 37 38 39 40 41 42 43 44 45 46 M
GUS gene
M 47 48 49 50 51 52 53 54 55 56 57 58 59 60 P M
GUS gene

(B)

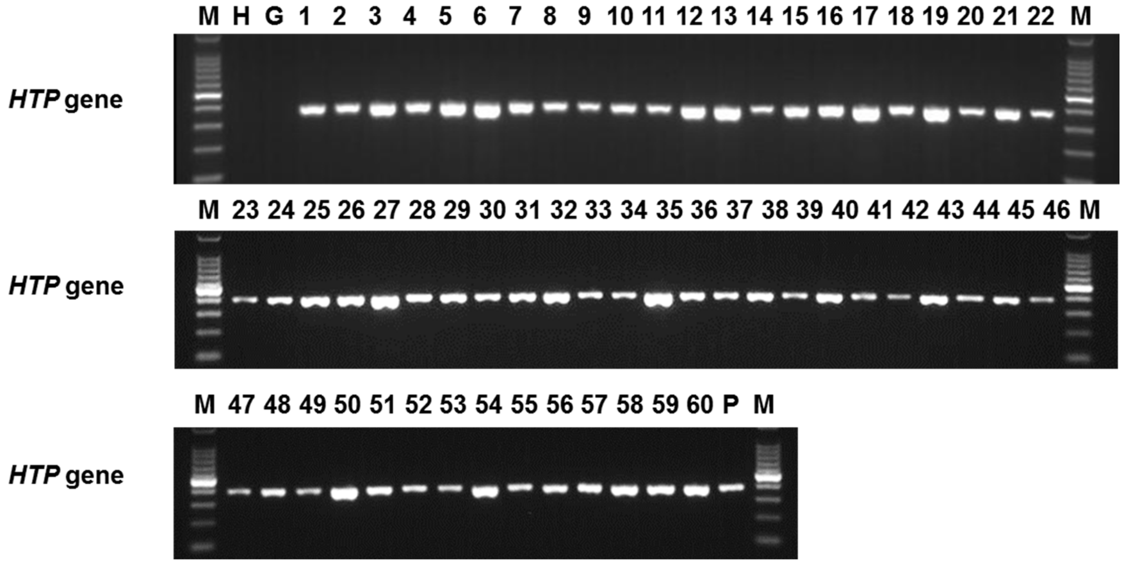
M H G 1 2 3 4 5 6 7 8 9 10 11 12 13 14 15 16 17 18 19 20 21 22 M
HTP gene
M 23 24 25 26 27 28 29 30 31 32 33 34 35 36 37 38 39 40 41 42 43 44 45 46 M
HTP gene
M 47 48 49 50 51 52 53 54 55 56 57 58 59 60 P M
HTP gene

Figure S3

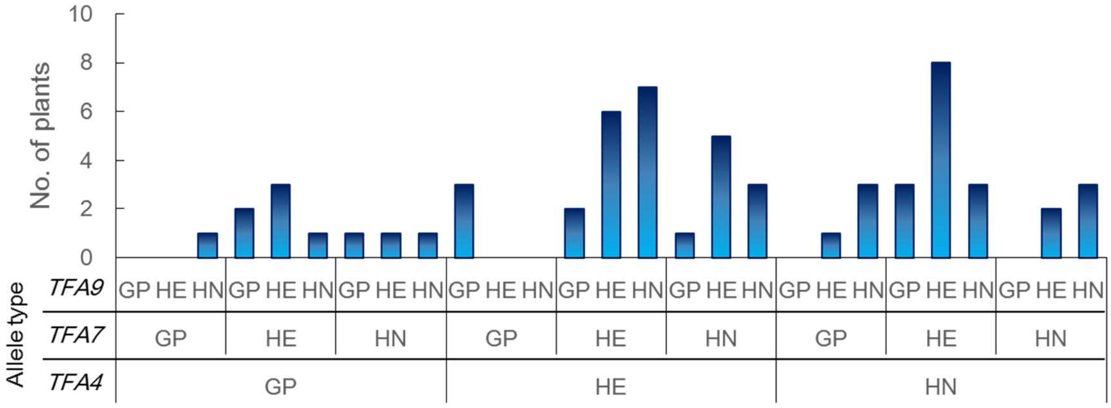

No. of plants
10
8
6
4
2
0
Allele type
TFA9
GP HE HN GP HE HN GP HE HN GP HE HN GP HE HN GP HE HN GP HE HN GP HE HN GP HE HN
TFA7
GP HE HN GP HE HN GP HE HN
TFA4
GP HE HN

Original image of gel for Figure 3B

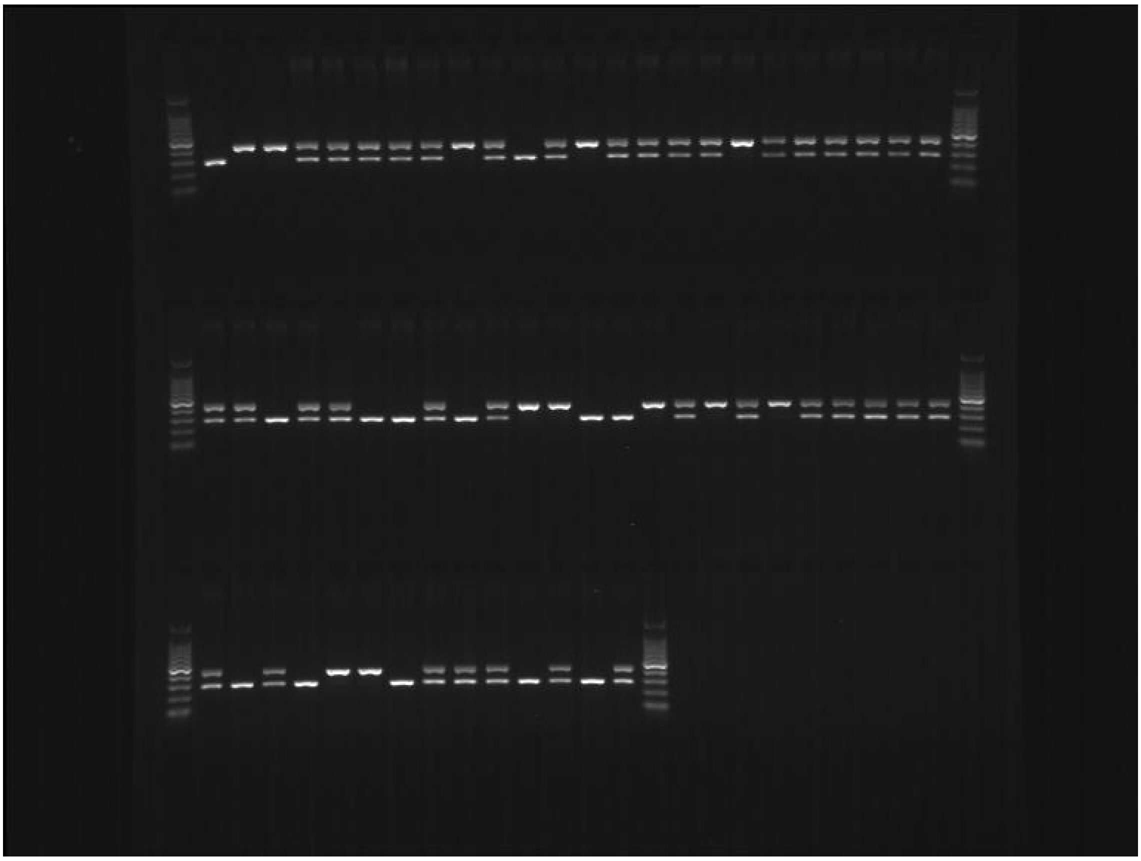

Table S1. Genotype of transgenic HN×GP plants, information of markers, and Chi-square values

Index	Chr	position (cM)	Name	Address	HN×GP-01	HN×GP-02	HN×GP-03	HN×GP-04	HN×GP-05	HN×GP-06	HN×GP-07	HN×GP-08	HN×GP-09	HN×GP-10	HN×GP-11	HN×GP-12	HN×GP-13	HN×GP-14	HN×GP-15	HN×GP-16
348	1H	0.95	337-641	5366	het	HN	GP	het	HN	het	GP	GP	HN	HN	het	GP	HN	GP	HN	het
164	1H	1.51	6195-2137	2205	het	HN	GP	het	HN	het	GP	GP	HN	HN	het	GP	HN	GP	HN	het
213	1H	23.86	1906-429	2915	HN	GP	het	het	GP	GP	GP	GP	het	HN	HN	GP	HN	het	het	HN
261	1H	26.58	6081-850	3901	HN	GP	het	het	GP	GP	het	GP	het	HN	HN	GP	HN	het	het	HN
183	1H	33.61	3751-1136	2398	HN	GP	het	het	GP	NC	het	het	het	HN	HN	het	HN	het	het	HN
292	1H	40.99	5381-1950	4075	het	GP	het	het	GP	GP	het	het	het	HN	HN	HN	HN	het	het	HN
316	1H	43.28	6720-641	5052	het	GP	het	het	GP	GP	het	het	het	HN	HN	HN	HN	het	het	HN
176	1H	54.73	7284-710	2322	het	GP	het	het	GP	GP	het	het	het	HN	HN	HN	HN	het	het	HN
152	1H	65.53	3675-2615	2059	het	GP	het	het	het	GP	het	het	het	HN	HN	HN	HN	het	het	HN
224	1H	99.19	3702-982	2999	HN	GP	het	HN	HN	GP	HN	het	het	HN	HN	HN	HN	HN	het	het
160	1H	106.60	3786-2204	2166	HN	GP	het	HN	het	het	het	het	het	HN	het	HN	HN	HN	het	het
105	1H	108.31	4962-1295	1601	HN	GP	het	HN	het	het	het	het	het	HN	het	HN	HN	HN	het	het
131	1H	112.54	5690-1045	1841	HN	GP	het	HN	het	het	het	het	het	HN	het	HN	HN	HN	het	het
265	1H	116.31	5048-1685	3919	HN	GP	het	HN	het	het	het	het	het	HN	het	HN	HN	HN	het	het
98	1H	125.27	4978-1030	1550	HN	GP	het	HN	het	het	het	het	het	het	HN	het	HN	HN	het	het
379	1H	127.10	4393-1078	6110	HN	GP	het	HN	het	het	het	het	het	het	HN	het	HN	HN	het	het
116	1H	131.89	5555-438	1711	het	het	het	HN	het	het	het	het	het	het	GP	HN	het	HN	het	GP
202	2H	10.94	3452-1355	2828	het	het	het	het	HN	HN	HN	het	HN	GP	het	het	het	het	GP	GP
201	2H	18.20	4277-1901	2808	het	HN	het	het	HN	HN	het	het	HN	GP	het	het	het	HN	GP	GP
110	2H	21.61	1865-396	1661	het	HN	het	het	HN	HN	het	het	HN	het	het	het	het	HN	GP	GP
296	2H	28.44	7747-1056	4107	het	HN	het	het	het	HN	GP	GP	het	het	het	HN	het	HN	het	GP
335	2H	29.15	7032-201	5221	het	HN	het	het	het	HN	GP	GP	het	het	het	HN	het	HN	het	GP
112	2H	37.32	1447-464	1682	het	HN	HN	het	het	het	GP	GP	GP	het	het	HN	het	HN	HN	het
358	2H	39.10	2964-382	5815	het	HN	HN	het	het	het	GP	GP	GP	het	het	HN	het	HN	HN	het
248	2H	41.66	4410-284	3606	het	HN	HN	het	het	het	GP	GP	GP	het	het	HN	het	HN	HN	het
102	2H	44.13	2651-1774	1588	het	HN	HN	het	het	het	GP	GP	GP	HN	het	HN	het	HN	HN	het
108	2H	54.95	2580-1456	1644	HN	HN	HN	het	het	het	GP	GP	GP	HN	GP	HN	het	HN	HN	het
66	2H	55.67	946-2500	1037	HN	HN	HN	het	het	het	GP	GP	GP	HN	GP	HN	het	HN	HN	het
199	2H	57.54	8889-842	2794	HN	HN	HN	het	het	het	GP	GP	GP	HN	GP	HN	het	HN	HN	het
194	2H	68.24	4434-804	2733	HN	HN	HN	het	het	het	GP	GP	GP	HN	GP	HN	het	HN	HN	het
3	2H	71.56	7187-382	33	HN	HN	HN	het	het	het	GP	GP	GP	HN	GP	HN	het	HN	HN	het
121	2H	73.04	334-1164	1772	HN	HN	HN	het	het	het	GP	GP	GP	HN	GP	HN	het	HN	HN	het
109	2H	75.18	4377-571	1647	HN	HN	HN	het	het	het	GP	GP	GP	HN	GP	HN	het	HN	HN	het
307	2H	78.03	1946-698	4950	HN	HN	HN	het	het	het	GP	het	GP	HN	GP	HN	het	HN	HN	het
132	2H	82.75	6117-1507	1850	HN	HN	HN	het	het	het	GP	het	GP	HN	GP	het	het	HN	HN	GP
354	2H	88.74	3469-1152	5784	HN	HN	HN	het	het	het	GP	het	GP	HN	GP	het	het	HN	het	GP
273	2H	90.10	8632-1809	3979	HN	HN	HN	het	het	het	GP	het	GP	HN	GP	het	het	HN	het	GP
195	2H	93.50	2020-539	2738	HN	HN	HN	het	het	het	GP	het	GP	HN	GP	het	het	HN	het	GP
217	2H	108.61	ABC04580-1-4-420	2954	GP	het	HN	het	het	het	GP	het	GP	HN	het	GP	het	het	GP	het
129	2H	112.91	111-499	1826	GP	het	het	het	het	het	GP	GP	GP	HN	het	GP	het	het	GP	het
368	2H	115.08	3180-1771	5930	GP	het	het	het	GP	het	GP	GP	GP	HN	het	GP	het	het	GP	het
134	2H	117.91	7576-818	1876	GP	het	het	het	GP	het	GP	GP	GP	HN	het	GP	GP	het	GP	het
339	2H	120.80	3256-1196	5285	GP	het	het	het	GP	het	GP	GP	GP	HN	HN	GP	GP	het	GP	het
137	3H	6.03	ConsensusGBS0194-	1888	HN	HN	het	het	het	het	HN	het	HN	het	GP	GP	het	het	GP	HN
94	3H	8.86	5945-748	1382	HN	HN	het	het	het	het	HN	GP	HN	het	GP	GP	het	het	GP	HN
260	3H	46.31	4105-1417	3896	het	het	GP	het	GP	HN	HN	het	het	GP	GP	GP	het	GP	het	het
173	3H	59.89	1746-1527	2308	het	het	GP	het	GP	HN	HN	het	het	GP	GP	GP	HN	GP	het	het
10	3H	62.99	5038-1035	113	het	het	GP	het	GP	HN	HN	het	het	GP	GP	GP	HN	GP	het	het
67	3H	65.52	4184-393	1042	het	het	GP	het	GP	HN	HN	het	het	GP	GP	GP	HN	GP	het	het
302	3H	78.53	1176-1547	4161	het	HN	GP	het	GP	HN	HN	het	het	GP	GP	GP	HN	GP	het	het
36	3H	88.82	8020-87	634	het	HN	GP	het	GP	HN	HN	het	het	GP	het	GP	het	GP	het	het
243	3H	123.68	76-1059	3484	het	HN	het	het	het	HN	HN	GP	het	GP	HN	het	het	GP	het	het
29	3H	126.27	5260-462	515	het	HN	het	het	het	HN	HN	GP	het	GP	HN	het	het	GP	het	het
294	3H	130.19	2335-1614	4092	het	HN	het	GP	het	HN	HN	GP	het	GP	HN	het	het	GP	het	het
123	3H	131.59	3718-1026	1777	het	HN	het	GP	het	HN	HN	GP	het	GP	HN	het	het	GP	het	het
289	3H	150.37	7818-967	4069	het	het	HN	GP	het	het	HN	GP	GP	GP	HN	het	het	GP	het	GP
93	3H	155.09	13750-348	1375	het	het	HN	GP	het	het	HN	het	GP	GP	het	het	het	GP	het	GP
49	4H	5.55	ABC14522-1-8-350	813	het	het	het	het	het	het	het	het	het	het	HN	HN	het	HN	HN	HN
170	4H	21.61	2055-947	2277	het	het	GP	het	het	GP	het	het	HN	het	HN	het	het	HN	HN	HN

233	4H	77.31	9149-1316	3072	het	GP	GP	GP	het	het	het	het	HN	het	HN	het	HN	het	HN	het
216	4H	81.69	1523-1136	2934	het	GP	GP	GP	het	HN	het	het	HN	het	het	het	HN	het	het	het
77	4H	84.30	4986-1214	1225	het	GP	GP	GP	het	HN	het	het	HN	het	het	het	HN	het	het	het
175	4H	87.49	4564-604	2320	het	GP	GP	GP	het	HN	het	het	HN	het	het	het	HN	het	het	het
240	4H	92.38	4361-1867	3468	GP	GP	het	GP	het	HN	het	het	het	het	het	het	HN	HN	het	het
319	4H	98.55	4535-1366	5073	GP	het	het	het	GP	HN	het	het	het	GP	het	het	HN	HN	GP	het
43	4H	100.74	41-695	732	GP	het	het	het	GP	HN	het	het	het	GP	het	het	HN	HN	GP	het
209	4H	106.03	5692-310	2898	GP	het	het	het	GP	HN	het	het	het	GP	GP	het	HN	HN	GP	het
186	5H	25.23	4753-1091	2420	GP	HN	het	het	HN	het	GP	het	HN	HN	het	GP	het	het	GP	het
20	5H	30.99	8377-1022	233	GP	HN	het	het	HN	het	GP	het	HN	HN	het	het	het	het	GP	het
214	5H	34.25	4684-775	2927	GP	HN	het	het	HN	het	GP	het	HN	HN	het	het	het	het	GP	het
151	5H	53.18	8320-955	2057	GP	HN	het	het	HN	GP	het	het	HN	het	het	het	het	het	GP	het
76	5H	70.48	264-571	1173	GP	HN	het	het	HN	GP	het	het	HN	HN	het	HN	het	het	GP	het
205	5H	102.06	5004-375	2861	HN	HN	het	het	HN	HN	het	GP	GP	HN	het	HN	het	GP	het	het
190	5H	122.38	139-1263	2501	HN	GP	GP	HN	GP	het	het	het	het	HN	het	HN	het	het	het	HN
167	5H	130.13	211-259	2232	HN	GP	GP	HN	GP	het	het	het	het	HN	het	HN	het	het	GP	HN
263	5H	135.72	5571-640	3908	HN	GP	GP	HN	GP	NC	het	HN	HN	HN	het	het	HN	het	GP	HN
222	5H	137.16	1394-1222	2994	HN	het	GP	HN	GP	het	het	HN	HN	HN	het	het	HN	het	GP	HN
54	5H	147.40	3883-616	913	HN	GP	GP	HN	GP	het	het	HN	HN	HN	HN	het	HN	het	GP	HN
309	5H	155.13	ConsensusGBS0451-	4969	HN	GP	GP	HN	het	GP	GP	HN	HN	HN	HN	het	HN	het	GP	HN
210	5H	159.09	603-72	2901	HN	GP	GP	HN	het	GP	GP	HN	HN	het	HN	het	HN	het	GP	HN
278	5H	171.66	ABC09278-1-4-69	4004	HN	GP	GP	HN	het	het	het	HN	HN	het	HN	HN	HN	het	GP	het
223	5H	191.97	2978-938	2995	HN	GP	GP	HN	HN	HN	het	HN	het	GP	HN	HN	HN	het	GP	het
57	5H	195.42	2726-852	950	HN	GP	GP	HN	HN	HN	het	het	het	GP	HN	HN	HN	het	het	het
200	6H	0.00	1692-742	2807	het	het	het	het	HN	het	HN	het	HN	het	GP	GP	het	het	het	GP
46	6H	1.34	5159-579	753	het	het	het	het	HN	het	HN	het	HN	het	GP	GP	het	het	het	GP
212	6H	3.11	ConsensusGBS0136-	2913	het	het	het	het	HN	het	HN	het	HN	het	GP	GP	het	het	het	GP
189	6H	12.54	ConsensusGBS0346-	2485	GP	GP	het	HN	het	het	het	het	HN	het	GP	GP	het	het	het	GP
187	6H	16.97	1769-545	2442	GP	GP	het	HN	GP	het	het	het	HN	GP	GP	GP	het	het	het	GP
120	6H	28.39	4611-178	1753	GP	GP	het	HN	het	GP	het	het	HN	GP	GP	het	het	het	het	het
37	6H	31.73	5771-91	651	GP	GP	het	het	het	het	GP	het	het	GP	GP	het	het	het	GP	het
118	6H	48.74	3378-619	1746	GP	GP	het	het	het	het	GP	het	het	GP	GP	het	het	HN	GP	het
117	6H	52.75	1009-1089	1740	GP	GP	het	het	het	het	GP	het	het	GP	GP	het	het	HN	GP	het
249	6H	55.36	5232-2041	3625	GP	GP	het	het	het	het	GP	het	het	GP	GP	het	het	HN	GP	het
305	6H	63.27	ConsensusGBS0369-	4908	GP	GP	het	het	het	het	GP	het	het	GP	GP	het	het	HN	GP	het
333	6H	64.36	3348-395	5184	GP	GP	het	het	het	het	GP	het	het	GP	GP	het	het	HN	GP	het
352	6H	67.04	4235-1617	5413	GP	GP	het	het	het	het	GP	het	het	GP	GP	het	het	HN	GP	het
330	6H	72.54	1096-1482	5157	GP	GP	het	het	het	het	GP	het	het	GP	GP	het	het	HN	GP	het
282	6H	77.89	7361-937	4022	GP	GP	het	het	het	het	GP	het	het	GP	GP	het	het	HN	GP	het
295	6H	81.17	ConsensusGBS0239-	4099	GP	GP	het	het	het	HN	GP	het	het	GP	GP	het	het	HN	GP	het
124	6H	105.60	428-1519	1784	GP	het	het	het	het	het	het	het	het	het	GP	het	het	HN	HN	GP
181	6H	110.32	2389-526	2361	GP	GP	GP	het	GP	het	het	HN	het	het	GP	het	het	HN	HN	GP
372	6H	119.02	1852-509	5985	GP	het	het	HN	het	het	het	HN	het	het	het	het	het	HN	HN	GP
90	6H	126.85	6523-1691	1333	GP	HN	het	HN	het	het	HN	HN	het	het	het	het	het	HN	HN	GP
18	6H	129.38	3363-1795	209	GP	HN	het	HN	het	het	HN	HN	GP	het	het	GP	het	het	HN	GP
206	7H	6.79	3359-1118	2862	HN	het	HN	het	het	het	GP	GP	het	het	HN	HN	het	GP	HN	het
172	7H	9.84	2148-498	2298	HN	HN	HN	het	het	het	GP	GP	het	het	HN	HN	het	GP	HN	het
140	7H	17.20	9672-758	1935	HN	HN	HN	het	het	het	GP	GP	het	het	HN	HN	het	GP	HN	het
55	7H	29.82	8365-454	915	het	HN	HN	het	het	GP	GP	GP	het	het	HN	HN	het	GP	HN	HN
381	7H	31.75	1404-64	6123	het	HN	HN	het	het	GP	GP	GP	het	het	HN	HN	het	het	HN	HN
15	7H	34.82	5777-354	162	het	HN	HN	het	het	GP	GP	GP	het	het	HN	HN	het	het	HN	HN
321	7H	46.19	ConsensusGBS0356-	5076	het	HN	het	het	het	het	GP	GP	het	het	HN	HN	het	HN	HN	het
1	7H	52.82	1792-372	10	het	HN	het	het	het	het	GP	GP	het	het	HN	HN	het	HN	het	het
114	7H	56.81	12239-662	1689	het	HN	het	het	het	het	GP	GP	het	het	HN	HN	het	HN	het	het
81	7H	60.69	943-3107	1269	het	het	het	het	GP	het	GP	GP	het	het	HN	HN	het	HN	het	het
56	7H	63.66	11912-654	920	het	HN	het	het	GP	het	GP	GP	het	het	HN	het	het	HN	het	het
276	7H	68.46	4475-478	3987	het	HN	het	het	het	het	GP	GP	het	het	HN	het	het	HN	het	het
349	7H	73.75	1735-1424	5381	het	HN	GP	het	het	het	het	GP	het	het	HN	het	het	HN	het	GP
148	7H	78.22	ABC09320-1-1-112	1990	het	HN	GP	het	het	het	het	GP	het	het	HN	het	GP	HN	het	GP
58	7H	87.97	2462-971	952	het	HN	GP	het	het	het	het	GP	het	het	HN	het	NC	HN	het	GP
182	7H	98.50	977-1377	2369	HN	HN	HN	HN	het	het	het	GP	het	het	HN	het	GP	HN	het	GP
31	7H	122.07	ABC10197-1-1-101	585	het	het	het	het	HN	het	het	GP	het	HN	het	HN	GP	het	GP	GP
5	7H	139.72	7023-448	54	het	het	het	het	het	HN	het	HN	het	het	GP	het	het	GP	GP	GP
13	7H	143.68	382-2624	153	het	het	het	het	het	HN	HN	HN	het	het	GP	het	het	GP	GP	het
113	7H	144.45	8785-443	1688	GP	het	HN	het	HN	het	GP	het	HN	HN	het	GP	het	het	GP	het

HN×GP-17	HN×GP-18	HN×GP-19	HN×GP-20	HN×GP-21	HN×GP-22	HN×GP-23	HN×GP-24	HN×GP-25	HN×GP-26	HN×GP-27	HN×GP-28	HN×GP-29	HN×GP-30	HN×GP-31	HN×GP-32	HN×GP-33	HN×GP-34	HN×GP-35	HN×GP-36	HN×GP-37	HN×GP-38	HN×GP-39	HN×GP-40	HN×GP-41	HN×GP-42	HN×GP-43	HN×GP-44
het	het	GP	GP	HN	het	GP	GP	HN	het	GP	HN	HN	het	het	HN	HN	het	het	het	HN	het	GP	het	HN	GP	HN	het
het	HN	GP	GP	HN	het	GP	GP	HN	het	GP	het	HN	het	het	HN	HN	het	het	het	HN	het	GP	het	HN	GP	HN	het
het	GP	GP	GP	HN	HN	het	het	het	het	het	het	HN	het	het	het	HN	HN	het	HN	GP	GP	GP	HN	het	het	HN	het
het	GP	GP	GP	HN	HN	het	het	het	het	het	het	HN	het	het	het	HN	HN	het	HN	GP	GP	GP	HN	het	het	HN	het
het	GP	GP	GP	HN	HN	het	het	het	het	het	het	HN	het	het	het	HN	het	het	HN	GP	het	GP	HN	het	HN	HN	het
het	GP	GP	het	het	HN	het	het	het	het	het	het	HN	het	het	het	HN	het	het	HN	GP	het	GP	HN	het	HN	het	het
het	GP	het	het	het	HN	het	het	het	het	het	het	HN	het	het	het	HN	het	het	HN	GP	het	GP	HN	het	HN	het	het
het	GP	het	het	het	HN	GP	het	het	het	het	het	HN	het	het	GP	HN	het	het	HN	GP	het	GP	HN	het	HN	GP	het
het	GP	het	het	het	HN	GP	het	het	het	het	het	HN	het	het	GP	HN	het	het	het	GP	GP	GP	HN	het	HN	GP	het
het	GP	het	HN	HN	HN	GP	het	het	HN	HN	het	GP	het	het	GP	GP	GP	het	het	GP	GP	het	HN	het	GP	GP	het
het	het	het	HN	HN	HN	het	het	het	HN	HN	het	GP	het	GP	GP	GP	GP	het	het	het	GP	het	HN	het	het	GP	GP
het	het	het	HN	HN	HN	het	het	het	HN	HN	het	GP	het	GP	GP	GP	GP	het	het	het	GP	het	HN	het	het	GP	GP
het	het	het	HN	HN	het	het	het	het	HN	het	het	GP	het	GP	GP	NC	het	het	het	het	GP	het	HN	het	het	het	GP
het	het	het	HN	HN	het	het	het	het	HN	het	het	GP	het	GP	GP	GP	het	het	het	het	GP	het	HN	HN	het	het	GP
het	HN	GP	het	HN	het	het	HN	het	HN	het	HN	GP	HN	GP	het	GP	het	het	het	het	GP	het	HN	HN	het	het	GP
het	HN	GP	het	HN	het	het	HN	het	HN	het	HN	GP	HN	GP	het	GP	het	het	het	het	GP	het	HN	HN	het	het	GP
het	HN	GP	het	het	het	het	HN	GP	HN	het	HN	GP	HN	GP	het	GP	het	het	het	het	GP	het	HN	HN	het	het	het
HN	GP	GP	het	GP	HN	HN	het	GP	HN	GP	het	HN	GP	het	GP	het	het	het	HN	het	het	HN	het	HN	het	het	het
HN	GP	GP	het	GP	HN	HN	het	GP	HN	GP	GP	het	GP	het	GP	het	het	GP	HN	het	GP	HN	het	HN	GP	het	het
HN	GP	GP	het	GP	HN	het	HN	GP	HN	GP	GP	GP	GP	het	GP	het	het	GP	HN	het	GP	het	GP	het	GP	het	GP
HN	GP	GP	het	GP	HN	het	HN	GP	HN	het	GP	GP	GP	het	GP	het	het	GP	HN	het	GP	het	GP	het	GP	het	GP
HN	GP	het	het	GP	HN	het	HN	GP	HN	het	GP	GP	GP	het	GP	het	het	GP	HN	het	GP	het	GP	het	GP	het	GP
HN	GP	het	het	GP	het	het	HN	GP	HN	het	GP	GP	het	het	GP	het	het	GP	het	het	GP	het	GP	het	GP	het	het
HN	GP	het	het	GP	het	het	HN	GP	HN	het	GP	GP	het	het	GP	het	het	GP	het	het	GP	het	GP	het	GP	het	het
HN	GP	het	het	GP	het	het	HN	GP	HN	het	GP	GP	het	het	GP	het	het	GP	het	GP	GP	het	GP	het	GP	het	het
HN	GP	het	het	GP	het	het	HN	GP	HN	het	GP	GP	het	het	GP	het	het	GP	het	GP	GP	het	GP	het	GP	het	het
HN	GP	GP	het	GP	het	het	HN	GP	HN	het	GP	GP	het	GP	GP	het	het	GP	het	GP	GP	het	GP	het	GP	het	het
HN	GP	GP	het	GP	het	het	HN	GP	HN	het	GP	GP	het	GP	GP	het	het	GP	het	GP	GP	het	GP	het	GP	het	het
HN	GP	GP	het	GP	het	het	HN	GP	HN	het	GP	GP	het	GP	GP	het	het	GP	het	GP	GP	het	GP	het	GP	het	het
HN	GP	GP	het	GP	het	het	HN	GP	HN	het	GP	GP	het	GP	GP	het	het	GP	het	GP	GP	het	GP	het	GP	het	het
HN	GP	GP	het	GP	het	het	HN	GP	HN	het	GP	GP	het	GP	GP	het	het	GP	het	GP	GP	het	GP	het	GP	het	het
HN	GP	GP	het	GP	het	het	HN	GP	HN	het	GP	GP	het	GP	GP	het	het	GP	het	GP	GP	het	GP	het	GP	het	het
HN	GP	GP	het	GP	het	het	HN	GP	HN	GP	GP	GP	het	GP	GP	het	het	GP	het	GP	GP	het	GP	het	GP	het	het
HN	GP	GP	GP	GP	het	het	HN	GP	HN	GP	GP	GP	het	GP	GP	het	het	het	het	GP	GP	het	GP	het	GP	het	het
HN	GP	GP	GP	GP	het	het	HN	GP	HN	GP	GP	GP	het	GP	GP	het	GP	het	het	GP	GP	het	GP	GP	GP	het	GP
HN	GP	GP	GP	GP	het	het	HN	GP	HN	GP	GP	GP	het	GP	GP	HN	GP	het	het	GP	GP	het	GP	GP	HN	het	GP
HN	GP	het	GP	GP	het	het	HN	GP	HN	GP	GP	GP	het	GP	GP	HN	GP	het	het	GP	GP	het	GP	GP	HN	het	GP
HN	GP	het	GP	GP	het	het	HN	GP	HN	GP	GP	GP	het	GP	GP	HN	GP	het	het	GP	GP	het	GP	GP	HN	het	GP
HN	GP	het	GP	het	het	het	het	GP	GP	GP	GP	GP	het	GP	het	HN	GP	het	het	GP	GP	HN	GP	HN	het	GP	het
het	GP	het	GP	het	het	het	het	GP	GP	het	GP	GP	het	GP	het	HN	GP	het	het	het	GP	HN	GP	HN	het	GP	het
het	GP	het	GP	het	het	het	het	GP	GP	het	GP	GP	het	GP	het	HN	het	het	GP	het	GP	HN	GP	HN	het	GP	het
het	GP	het	GP	het	het	het	het	het	GP	het	GP	het	GP	GP	het	HN	het	het	GP	het	GP	het	GP	HN	het	GP	het
het	GP	het	GP	het	het	het	het	het	GP	het	GP	het	GP	GP	het	HN	het	het	GP	het	GP	het	GP	HN	het	GP	het
HN	het	het	het	HN	HN	GP	het	HN	het	HN	het	HN	HN	het	het	HN	het	het	HN	het	HN	GP	het	HN	GP	HN	het
HN	het	het	het	HN	HN	GP	het	HN	het	HN	het	HN	HN	het	het	HN	het	het	HN	het	HN	het	het	HN	GP	HN	het
GP	GP	GP	GP	GP	GP	GP	GP	het	GP	het	GP	GP	het	GP	GP	het	GP	GP	GP	HN	GP	het	het	het	GP	het	GP
GP	GP	GP	GP	GP	GP	GP	GP	het	GP	het	GP	GP	het	GP	GP	het	GP	GP	GP	HN	GP	het	het	het	GP	het	GP
GP	GP	GP	GP	GP	GP	GP	GP	het	GP	het	GP	GP	het	GP	GP	het	GP	GP	GP	HN	GP	het	het	het	GP	het	GP
GP	GP	GP	GP	GP	GP	GP	GP	het	GP	het	GP	GP	het	GP	GP	het	GP	GP	GP	HN	GP	het	het	het	GP	het	GP
GP	GP	GP	GP	het	GP	GP	het	het	GP	het	GP	GP	het	GP	GP	het	GP	GP	GP	HN	GP	het	het	het	GP	het	het
GP	GP	GP	GP	het	GP	GP	het	het	GP	het	GP	GP	het	GP	het	HN	GP	GP	GP	HN	GP	het	het	het	GP	het	het
GP	GP	HN	het	HN	het	GP	HN	het	het	HN	GP	GP	het	GP	GP	het	het	het	GP	het	het	het	het	het	GP	GP	HN
GP	GP	HN	het	HN	het	GP	HN	het	het	HN	GP	GP	het	het	GP	het	het	het	GP	het	het	het	het	het	GP	GP	HN
GP	GP	HN	het	HN	het	het	HN	het	het	HN	GP	GP	het	het	GP	het	het	het	GP	het	het	het	het	GP	GP	GP	het
GP	GP	HN	het	HN	het	het	HN	het	het	HN	het	GP	het	het	GP	het	het	het	GP	het	het	het	het	GP	GP	GP	het
het	het	het	het	HN	het	het	HN	het	het	HN	HN	GP	het	GP	GP	GP	het	HN	GP	GP	het	GP	het	GP	GP	GP	GP
HN	het	het	GP	HN	het	het	HN	het	het	HN	het	GP	GP	GP	GP	GP	het	HN	GP	het	het	GP	het	GP	GP	het	GP
het	HN	het	GP	het	het	GP	het	het	het	het	het	het	HN	HN	GP	het	het	GP	het	GP	HN	HN	HN	het	GP	HN	HN
het	HN	het	het	het	HN	GP	HN	HN	het	het	HN	het	HN	HN	het	het	het	het	GP	HN	HN	HN	het	GP	GP	het	het

het	GP	het	het	het	HN	GP	HN	GP	HN	het	HN	HN	HN	het	HN	het	GP	het	het	HN	het	GP	GP	GP	het	GP	het
het	GP	het	het	het	het	GP	HN	het	HN	het	HN	HN	het	het	HN	het	GP	het	het	HN	het	GP	het	GP	het	GP	het
het	GP	het	het	het	het	GP	HN	het	HN	het	HN	HN	het	het	HN	het	GP	het	het	het	het	GP	het	GP	het	GP	het
het	GP	het	het	het	het	GP	HN	het	HN	het	HN	het	het	het	HN	het	het	het	het	het	het	GP	het	GP	het	GP	het
het	GP	HN	HN	het	het	GP	het	het	HN	het	het	het	het	HN	HN	het	het	het	het	het	het	GP	het	GP	het	GP	het
het	GP	het	HN	het	HN	GP	het	het	HN	GP	het	het	het	HN	het	GP	het	het	het	GP	het	GP	het	GP	het	GP	het
het	GP	het	HN	het	HN	GP	het	het	HN	GP	het	het	het	HN	het	GP	het	het	het	GP	het	GP	het	GP	het	GP	het
het	GP	het	HN	HN	HN	GP	het	het	HN	GP	het	het	het	HN	het	GP	het	het	het	GP	het	GP	het	GP	het	GP	GP

het	HN	HN	het	HN	HN	HN	HN	GP	het	HN	GP	HN	HN	het	het	HN	GP	HN	het	HN	het	het	het	HN	HN	HN	HN
het	HN	HN	het	HN	HN	HN	het	het	het	HN	GP	HN	HN	het	GP	HN	GP	HN	HN	HN	het	het	het	HN	HN	HN	HN
het	HN	HN	het	HN	HN	HN	het	het	het	HN	GP	HN	HN	het	GP	HN	GP	HN	HN	HN	het	het	het	HN	HN	HN	HN
GP	het	het	het	het	HN	het	het	het	het	HN	GP	het	HN	het	GP	HN	GP	HN	HN	HN	het	het	het	HN	HN	HN	HN
GP	het	het	het	het	het	het	het	het	het	HN	GP	het	HN	het	GP	HN	GP	het	HN	HN	het	het	het	HN	HN	HN	HN
GP	het	het	GP	het	het	het	GP	GP	HN	HN	HN	het	HN	het	GP	het	GP	GP	het	HN	het	het	GP	GP	het	HN	het
het	HN	HN	GP	het	HN	HN	het	GP	het	GP	HN	HN	het	het	het	het	het	het	het	HN	het	GP	GP	GP	het	het	GP
het	het	HN	het	het	HN	HN	het	GP	het	GP	HN	HN	GP	het	het	het	het	het	het	HN	GP	GP	het	het	het	het	het
het	het	HN	het	het	HN	HN	het	GP	het	GP	het	HN	GP	het	het	het	het	het	het	HN	GP	GP	het	het	het	het	het
het	het	HN	het	het	HN	HN	het	GP	het	GP	het	HN	het	het	het	het	het	het	het	HN	GP	het	het	het	het	het	het
het	het	het	het	GP	HN	HN	het	HN	het	GP	het	het	het	het	GP	NC	NC	het	het	het	het	het	HN	HN	het	het	het
GP	HN	het	het	GP	HN	HN	het	het	het	het	het	het	het	het	GP	het	het	het	het	het	HN	het	HN	HN	HN	HN	het
GP	HN	het	het	GP	HN	HN	het	het	het	het	het	het	het	het	GP	het	het	het	het	het	HN	het	het	HN	HN	HN	het
GP	HN	het	het	GP	HN	HN	het	het	GP	het	het	het	het	het	GP	het	het	het	het	het	HN	het	het	HN	HN	HN	het
het	het	het	het	het	HN	HN	het	het	GP	het	HN	het	het	GP	GP	het	GP	het	het	het	HN	het	het	het	HN	het	het
het	het	het	het	het	HN	HN	het	het	GP	het	HN	het	het	GP	GP	het	GP	het	het	HN	het	het	het	HN	HN	het	GP

het	het	het	HN	HN	GP	het	het	het	het	het	het	GP	GP	HN	HN	HN	HN	het	HN	het	het	het	HN	het	het	GP	het
het	het	het	HN	HN	GP	het	het	het	het	het	het	GP	GP	HN	HN	HN	HN	het	HN	het	het	het	HN	het	het	GP	het
het	het	het	HN	HN	GP	het	het	het	het	het	het	GP	GP	HN	HN	HN	HN	het	HN	het	het	het	HN	het	het	GP	het
HN	het	het	het	het	het	GP	het	HN	het	het	het	GP	GP	het	HN	het	het	GP	HN	het	het	het	HN	GP	het	GP	het
het	het	het	het	het	het	GP	het	HN	het	het	het	het	GP	het	HN	het	het	het	HN	GP	het	het	HN	GP	het	GP	het
het	het	GP	het	het	HN	GP	het	HN	het	HN	HN	het	GP	het	HN	het	het	het	HN	GP	het	het	HN	GP	HN	GP	het
het	HN	GP	het	GP	HN	GP	het	HN	het	HN	HN	het	GP	het	HN	het	het	het	HN	GP	het	het	HN	GP	het	GP	het
het	HN	GP	het	GP	HN	het	het	HN	het	het	HN	HN	het	het	het	GP	het	HN	HN	GP	het	GP	GP	het	het	het	het
het	HN	GP	het	GP	HN	het	het	HN	het	het	HN	HN	het	het	het	GP	het	HN	HN	GP	het	GP	GP	het	het	het	het
het	HN	GP	het	GP	HN	het	het	HN	het	het	HN	HN	het	het	het	GP	het	HN	HN	GP	het	GP	GP	het	het	het	het
het	HN	GP	het	GP	HN	het	het	HN	het	het	HN	HN	het	het	het	GP	het	HN	HN	GP	het	GP	GP	het	het	het	het
het	HN	GP	het	GP	HN	het	het	HN	het	het	HN	HN	het	het	het	GP	GP	HN	HN	GP	het	GP	GP	GP	het	het	het
het	HN	GP	het	GP	HN	het	het	HN	het	het	HN	HN	het	het	het	GP	GP	HN	HN	GP	het	GP	het	GP	het	het	het
het	HN	het	het	GP	het	het	het	HN	het	het	HN	HN	het	het	het	GP	GP	HN	HN	GP	het	GP	het	GP	het	het	het
het	HN	het	het	het	het	het	het	HN	het	het	HN	HN	het	het	het	GP	GP	HN	HN	GP	het	GP	het	GP	het	het	het
het	HN	het	het	het	het	het	het	HN	het	het	HN	HN	het	het	het	GP	GP	HN	HN	GP	het	GP	het	GP	het	het	het
het	GP	GP	het	HN	het	HN	GP	HN	HN	het	HN	het	het	HN	HN	GP	GP	HN	het	het	HN	GP	HN	GP	het	het	het
het	GP	GP	het	HN	het	HN	het	HN	HN	het	HN	het	het	HN	HN	GP	GP	HN	het	het	HN	GP	HN	het	het	het	het
HN	het	het	GP	HN	het	HN	het	HN	het	het	HN	het	het	HN	HN	GP	GP	het	het	HN	het	GP	HN	HN	het	GP	het
HN	het	het	GP	het	het	HN	HN	HN	het	het	het	GP	het	HN	HN	GP	GP	het	het	HN	het	GP	HN	HN	GP	GP	het
HN	het	het	GP	het	het	HN	HN	HN	het	het	het	GP	het	het	het	GP	het	het	HN	HN	GP	GP	HN	HN	GP	GP	het

het	GP	GP	het	het	het	GP	HN	HN	het	het	GP	GP	het	het	GP	GP	HN	GP	HN	het	HN	het	het	het	het	GP	HN
het	GP	GP	het	het	het	het	HN	HN	het	het	GP	GP	het	het	GP	GP	HN	GP	HN	het	HN	het	het	het	het	GP	HN
HN	GP	GP	het	GP	het	het	HN	HN	het	het	GP	GP	het	het	GP	GP	HN	GP	het	het	HN	het	het	het	het	GP	HN
het	GP	GP	het	GP	het	het	HN	HN	het	het	GP	GP	het	GP	GP	GP	HN	het	het	HN	HN	HN	het	het	het	het	HN
het	GP	GP	het	GP	het	het	HN	HN	het	het	GP	GP	het	GP	GP	het	HN	het	het	HN	HN	HN	het	het	het	het	HN
het	het	GP	het	GP	het	het	HN	HN	het	het	GP	GP	het	GP	GP	het	HN	het	het	HN	HN	HN	het	het	het	HN	het
het	HN	GP	GP	GP	GP	het	HN	HN	het	het	het	GP	GP	GP	GP	het	HN	het	HN	HN	HN	het	HN	het	GP	HN	GP
het	HN	het	het	GP	GP	HN	HN	HN	het	het	HN	GP	GP	het	het	het	HN	het	HN	HN	HN	het	HN	het	GP	het	GP
het	HN	het	het	GP	GP	HN	HN	HN	het	het	HN	GP	GP	het	het	het	HN	het	HN	HN	HN	het	HN	het	GP	het	GP
het	HN	het	het	GP	GP	HN	HN	HN	het	het	HN	GP	GP	het	HN	het	HN	het	HN	HN	HN	het	HN	het	GP	het	GP
het	HN	het	het	het	het	HN	HN	HN	het	het	HN	het	GP	het	HN	het	HN	het	HN	HN	het	het	HN	het	GP	het	GP
het	HN	het	het	het	het	HN	HN	HN	het	GP	HN	het	GP	het	HN	het	HN	het	HN	HN	het	het	HN	het	GP	het	GP
het	HN	het	het	het	het	HN	HN	HN	het	GP	HN	het	GP	het	HN	het	HN	het	HN	HN	het	het	HN	het	GP	het	het
het	HN	het	het	het	het	het	HN	HN	het	GP	HN	het	GP	het	HN	het	HN	het	HN	HN	GP	het	HN	het	GP	het	het
het	HN	HN	het	het	het	het	HN	HN	het	GP	HN	het	GP	het	HN	het	HN	het	HN	HN	GP	het	HN	het	GP	het	het
het	HN	HN	het	GP	het	HN	HN	HN	het	GP	HN	het	GP	het	HN	het	HN	het	HN	HN	GP	het	HN	het	GP	het	het
het	het	HN	het	het	HN	het	het	het	het	GP	HN	het	GP	het	HN	het	het	GP	HN	het	het	het	HN	GP	GP	het	GP
het	HN	het	het	het	HN	het	het	GP	HN	het	HN	het	GP	het	het	het	het	GP	HN	het	HN	HN	het	GP	GP	HN	GP
het	HN	het	het	het	HN	het	het	GP	HN	het	HN	het	GP	het	het	HN	het	GP	HN	het	HN	het	het	GP	GP	HN	GP
het	HN	HN	het	HN	HN	HN	HN	GP	het	HN	GP	HN	HN	het	het	HN	GP	HN	het	HN	het	het	het	het	HN	HN	HN

	HN×GP-45	HN×GP-46	HN×GP-47	HN×GP-48	HN×GP-49	HN×GP-50	HN×GP-51	HN×GP-52	HN×GP-53	HN×GP-54	HN×GP-55	HN×GP-56	HN×GP-57	HN×GP-58	HN×GP-59	HN×GP-60	No. of HN	No. of GP	No. of het	χ^2 value (1:2:1)	χ^2 value (3:1, [GP+het]:[HN])	χ^2 value (3:1, [HN+het]:[GP])
1	HN	het	HN	GP	het	GP	het	GP	het	het	het	het	het	HN	GP	GP	18	17	25	1.70	0.80	0.36
2	HN	het	HN	het	het	GP	het	GP	het	het	het	het	het	HN	GP	GP	18	16	26	1.20	0.80	0.09
3	HN	HN	het	GP	GP	het	het	GP	het	HN	GP	HN	het	GP	het	het	17	17	26	1.07	0.36	0.36
4	HN	HN	het	GP	GP	het	het	GP	het	HN	GP	HN	het	GP	het	het	17	16	27	0.63	0.36	0.09
5	HN	HN	het	GP	GP	het	het	GP	het	HN	GP	HN	het	GP	het	het	17	12	30	0.86	0.46	0.68
6	HN	HN	het	GP	GP	het	HN	GP	het	HN	GP	HN	het	GP	het	het	16	12	32	0.80	0.09	0.80
7	HN	HN	het	GP	GP	het	HN	GP	het	HN	GP	HN	het	GP	het	het	16	11	33	1.43	0.09	1.42
8	HN	HN	het	GP	GP	het	HN	GP	het	HN	GP	HN	het	GP	het	het	16	14	30	0.13	0.09	0.09
9	HN	HN	het	GP	GP	het	HN	GP	GP	HN	het	het	het	GP	het	het	14	14	32	0.27	0.09	0.09
10	het	HN	het	GP	het	het	HN	GP	GP	HN	het	het	HN	GP	HN	GP	20	17	23	3.57	2.22	0.36
11	het	het	het	GP	het	het	HN	GP	GP	HN	het	het	HN	GP	HN	GP	16	14	30	0.13	0.09	0.09
12	het	het	het	GP	het	het	HN	GP	GP	HN	het	het	HN	GP	HN	GP	16	14	30	0.13	0.09	0.09
13	het	het	het	GP	HN	het	HN	GP	GP	HN	het	het	HN	GP	HN	GP	15	11	33	1.37	0.01	1.27
14	het	het	het	GP	HN	het	het	GP	GP	HN	het	het	HN	GP	het	het	14	11	35	1.97	0.09	1.42
15	het	GP	het	HN	HN	het	GP	GP	het	HN	het	het	HN	GP	het	HN	17	11	32	1.47	0.36	1.42
16	het	GP	het	HN	HN	het	GP	GP	het	HN	het	het	HN	GP	het	HN	17	11	32	1.47	0.36	1.42
17	het	GP	het	HN	HN	het	GP	het	het	HN	GP	het	HN	GP	het	HN	15	12	33	0.90	0.00	0.80
18	GP	GP	HN	het	het	HN	GP	het	het	het	GP	het	het	GP	het	GP	14	16	30	0.13	0.09	0.09
19	GP	GP	HN	het	het	HN	GP	het	het	het	GP	het	GP	GP	het	GP	14	21	25	3.30	0.09	3.20
20	GP	GP	het	het	het	HN	het	HN	GP	het	het	het	GP	GP	het	GP	12	22	26	4.40	0.80	4.36
21	GP	GP	het	het	het	HN	het	HN	GP	HN	het	het	GP	GP	het	GP	12	22	26	4.40	0.80	4.36
22	GP	GP	het	GP	het	HN	het	HN	GP	HN	het	het	GP	GP	het	GP	12	22	26	4.40	0.80	4.36
23	GP	GP	het	GP	het	het	HN	het	GP	het	het	HN	GP	GP	het	GP	10	20	30	3.33	2.22	2.22
24	GP	GP	het	GP	het	het	HN	het	GP	het	het	HN	GP	GP	het	GP	10	20	30	3.33	2.22	2.22
25	GP	GP	het	GP	het	het	HN	het	GP	het	het	HN	GP	GP	het	GP	10	21	29	4.10	2.22	3.20
26	GP	GP	HN	GP	het	het	HN	het	GP	het	het	HN	GP	GP	het	GP	12	21	27	3.30	0.80	3.20
27	GP	GP	HN	GP	het	het	HN	GP	GP	het	het	HN	GP	GP	het	GP	13	25	22	9.07	0.36	8.89
28	GP	GP	HN	GP	het	het	HN	GP	GP	het	het	HN	GP	GP	het	GP	13	25	22	9.07	0.36	8.89
29	GP	GP	HN	GP	het	het	HN	GP	GP	het	het	HN	GP	GP	het	GP	13	25	22	9.07	0.36	8.89
30	GP	GP	HN	GP	het	het	HN	GP	GP	het	het	HN	GP	GP	het	GP	13	25	22	9.07	0.36	8.89
31	GP	GP	HN	GP	het	het	HN	GP	GP	het	het	HN	GP	GP	het	GP	13	25	22	9.07	0.36	8.89
32	GP	GP	HN	GP	het	het	HN	GP	GP	het	het	HN	GP	GP	het	GP	13	25	22	9.07	0.36	8.89
33	GP	GP	het	GP	het	het	HN	GP	GP	het	het	HN	GP	GP	het	GP	12	26	22	10.80	0.80	10.76
34	GP	GP	GP	GP	het	het	HN	GP	GP	het	het	HN	GP	GP	het	GP	12	26	22	10.80	0.80	10.76
35	GP	GP	GP	GP	het	het	HN	GP	GP	het	GP	HN	GP	GP	het	GP	11	31	18	22.93	1.42	22.76
36	GP	GP	GP	GP	het	het	HN	GP	GP	het	GP	HN	GP	GP	HN	het	13	29	18	18.13	0.36	17.42
37	GP	GP	GP	GP	het	het	HN	GP	GP	het	GP	HN	GP	GP	HN	het	13	28	19	15.57	0.36	15.02
38	GP	GP	GP	GP	het	GP	HN	GP	GP	het	GP	HN	GP	GP	HN	het	13	29	18	18.13	0.36	17.42
39	het	GP	GP	het	GP	GP	HN	GP	GP	het	GP	het	GP	het	het	het	7	26	27	12.63	5.69	10.76
40	het	GP	GP	het	GP	GP	HN	GP	GP	het	GP	het	het	het	het	het	5	24	31	12.10	8.89	7.20
41	het	GP	GP	het	GP	GP	het	GP	GP	het	GP	het	het	het	het	HN	5	25	30	13.33	8.89	8.89
42	het	GP	GP	het	GP	GP	het	het	GP	het	GP	het	het	het	het	HN	4	24	32	13.60	10.76	7.20
43	het	GP	GP	het	GP	GP	het	het	GP	het	GP	het	het	het	het	HN	5	24	31	12.10	8.89	7.20
44	het	HN	het	GP	het	GP	het	het	GP	GP	het	GP	HN	HN	GP	HN	21	12	27	3.30	3.20	0.80
45	GP	HN	het	GP	GP	GP	het	het	GP	GP	het	GP	het	HN	GP	HN	20	14	26	2.27	2.22	0.09
46	GP	GP	HN	GP	GP	GP	GP	GP	GP	GP	GP	GP	GP	het	GP	GP	4	39	17	52.10	10.76	51.20
47	GP	GP	HN	GP	GP	GP	GP	GP	GP	GP	GP	GP	GP	het	GP	GP	5	39	16	51.60	8.89	51.20
48	GP	GP	HN	GP	GP	GP	GP	GP	GP	GP	GP	GP	GP	het	GP	GP	5	39	16	51.60	8.89	51.20
49	GP	GP	HN	GP	GP	GP	GP	GP	GP	GP	GP	GP	GP	het	GP	GP	5	39	16	51.60	8.89	51.20
50	GP	GP	HN	GP	GP	GP	GP	GP	GP	het	GP	GP	GP	het	GP	GP	6	35	19	36.10	7.20	35.56
51	GP	GP	het	GP	GP	GP	GP	GP	GP	GP	HN	GP	GP	het	GP	GP	6	33	21	29.70	7.20	28.80
52	GP	het	het	GP	het	HN	het	het	GP	GP	HN	GP	het	HN	het	het	12	18	30	1.20	0.80	0.80
53	GP	het	het	GP	het	HN	het	het	GP	GP	het	GP	het	HN	het	het	11	17	32	1.47	1.42	0.36
54	het	het	het	GP	HN	HN	het	het	GP	GP	het	GP	het	HN	het	het	11	17	32	1.47	1.42	0.36
55	het	het	het	GP	HN	HN	het	het	GP	GP	het	GP	het	HN	het	het	11	16	33	1.43	1.42	0.09
56	het	het	het	GP	GP	HN	HN	het	GP	GP	het	het	het	HN	GP	het	11	22	27	4.63	1.42	4.36
57	het	het	het	GP	GP	HN	HN	het	GP	GP	het	het	het	HN	GP	het	10	21	29	4.10	2.22	3.20
58	HN	het	het	het	HN	het	het	het	GP	het	het	het	het	het	het	HN	16	7	37	5.97	0.09	5.69
59	HN	GP	het	het	het	het	het	het	GP	HN	het	het	het	het	het	het	17	8	35	4.37	0.36	4.36

het	GP	het	GP	GP	het	HN	GP	het	het	het	GP	het	GP	GP	het	13	18	29	0.90	0.36	0.80
het	GP	het	GP	GP	het	HN	GP	het	het	het	GP	het	GP	GP	het	10	16	34	2.27	2.22	0.09
het	GP	het	GP	GP	het	het	GP	het	GP	het	GP	het	GP	GP	het	8	17	35	4.37	4.36	0.36
het	GP	het	GP	GP	het	het	GP	het	GP	het	GP	het	GP	GP	het	7	16	37	5.97	5.69	0.09
het	het	het	het	GP	het	het	GP	het	GP	HN	GP	het	GP	GP	het	9	14	37	4.10	3.20	0.09
HN	het	het	het	GP	het	GP	GP	het	GP	HN	GP	het	GP	GP	het	9	19	32	3.60	3.20	1.42
HN	HN	het	het	GP	het	GP	GP	het	GP	HN	GP	het	GP	GP	het	10	19	31	2.77	2.22	1.42
HN	HN	GP	het	het	het	GP	GP	het	GP	HN	GP	het	GP	GP	het	11	21	28	3.60	1.42	3.20

het	het	het	het	HN	HN	GP	het	GP	GP	GP	het	GP	GP	GP	het	22	14	24	4.53	4.36	0.09
het	het	het	het	HN	HN	het	het	GP	GP	HN	het	het	GP	GP	het	23	10	27	6.23	5.69	2.22
het	het	het	het	HN	HN	het	het	GP	GP	HN	het	het	GP	GP	het	23	10	27	6.23	5.69	2.22
het	het	het	het	HN	HN	HN	HN	GP	het	HN	het	het	GP	GP	het	19	10	31	2.77	1.42	2.22
het	het	GP	het	HN	HN	HN	HN	GP	het	HN	het	het	GP	GP	het	19	11	30	2.13	1.42	1.42
GP	het	GP	GP	HN	HN	HN	het	GP	het	het	het	het	GP	GP	GP	15	19	26	1.60	0.00	1.42
GP	GP	het	HN	HN	het	HN	het	HN	het	het	GP	HN	HN	GP	het	18	14	28	0.80	0.80	0.09
GP	GP	het	HN	HN	GP	HN	het	HN	het	het	GP	het	HN	GP	het	16	14	30	0.13	0.09	0.09
GP	GP	het	HN	HN	GP	HN	het	HN	het	het	GP	het	HN	het	het	17	13	29	0.56	0.46	0.28
GP	GP	het	HN	HN	GP	HN	het	HN	het	het	GP	het	HN	het	het	17	10	33	2.23	0.36	2.22
GP	GP	het	het	HN	GP	HN	het	HN	HN	het	GP	HN	HN	het	het	19	11	28	2.28	1.86	1.13
GP	GP	het	het	HN	GP	HN	HN	HN	HN	het	GP	HN	het	het	het	22	12	26	4.40	4.36	0.80
GP	GP	het	het	HN	GP	HN	HN	HN	HN	het	GP	HN	het	het	het	20	12	28	2.40	2.22	0.80
GP	GP	het	het	HN	het	HN	HN	HN	HN	GP	HN	HN	het	het	het	21	10	29	4.10	3.20	2.22
het	het	HN	HN	het	het	HN	het	HN	HN	GP	HN	het	het	HN	HN	21	9	30	4.80	3.20	3.20
het	het	HN	HN	het	het	het	het	HN	HN	GP	HN	het	het	HN	HN	20	9	31	4.10	2.22	3.20

GP	het	het	het	GP	het	het	GP	het	GP	GP	het	HN	het	het	het	12	12	36	2.40	0.80	0.80
GP	het	het	het	GP	GP	het	GP	het	GP	GP	het	HN	het	het	het	12	13	35	1.70	0.80	0.36
GP	het	het	het	GP	GP	het	GP	het	GP	GP	het	HN	het	het	het	12	13	35	1.70	0.80	0.36
GP	GP	het	het	het	GP	het	GP	het	het	GP	GP	HN	het	GP	het	8	18	34	4.40	4.36	0.80
GP	GP	het	het	het	het	het	GP	het	HN	GP	GP	HN	het	GP	GP	8	19	33	4.63	4.36	1.42
het	het	het	HN	HN	het	het	GP	het	HN	GP	GP	HN	het	het	GP	14	15	31	0.10	0.09	0.00
het	het	het	HN	HN	het	het	GP	het	HN	het	GP	HN	het	het	GP	12	16	32	0.80	0.80	0.09
HN	het	het	HN	HN	het	het	GP	het	HN	het	het	HN	het	het	GP	13	14	33	0.63	0.36	0.09
HN	het	het	HN	HN	het	het	GP	het	HN	het	het	HN	het	het	GP	13	14	33	0.63	0.36	0.09
HN	het	het	HN	HN	het	het	GP	het	het	het	het	HN	het	het	GP	12	14	34	1.20	0.80	0.09
het	het	het	HN	HN	het	het	GP	het	het	het	het	HN	het	het	GP	11	14	35	1.97	1.42	0.09
het	het	het	HN	HN	het	het	GP	het	het	het	het	HN	het	het	GP	11	16	33	1.43	1.42	0.09
het	het	het	HN	HN	het	het	GP	het	het	het	het	HN	het	HN	GP	12	15	33	0.90	0.80	0.00
het	het	het	HN	HN	het	het	GP	het	het	het	het	HN	het	HN	GP	11	14	35	1.97	1.42	0.09
het	het	het	HN	HN	het	het	GP	het	het	het	het	HN	het	HN	GP	11	13	36	2.53	1.42	0.36
het	het	het	HN	HN	HN	het	GP	het	het	het	het	HN	het	HN	GP	13	13	34	1.07	0.36	0.36
het	GP	het	HN	het	HN	GP	GP	HN	het	het	het	het	GP	HN	het	16	14	30	0.13	0.09	0.09
het	GP	het	HN	het	HN	GP	GP	het	het	het	het	het	het	HN	het	16	14	30	0.13	0.09	0.09
GP	GP	het	HN	het	HN	het	GP	het	het	het	het	het	het	het	het	16	10	34	2.27	0.09	2.22
GP	GP	het	het	het	HN	het	GP	het	het	het	GP	het	het	het	het	16	13	31	0.37	0.09	0.36
GP	GP	GP	GP	GP	HN	het	GP	het	GP	het	GP	het	het	het	het	14	19	27	1.43	0.09	1.42

GP	GP	het	het	het	GP	HN	het	het	GP	GP	GP	het	het	het	GP	12	19	29	1.70	0.80	1.42
GP	GP	het	het	het	GP	HN	het	het	GP	GP	GP	het	het	het	GP	13	18	29	0.90	0.36	0.80
GP	GP	het	het	het	GP	het	GP	het	GP	GP	GP	GP	het	het	GP	12	21	27	3.30	0.80	3.20
GP	het	het	het	het	GP	GP	GP	GP	het	het	GP	GP	het	GP	GP	13	21	26	3.20	0.36	3.20
GP	het	het	het	het	GP	GP	GP	GP	het	het	GP	GP	het	GP	GP	13	19	28	1.47	0.36	1.42
GP	het	het	het	het	GP	GP	GP	GP	het	het	het	GP	het	GP	GP	13	17	30	0.53	0.36	0.36
het	het	het	HN	het	GP	GP	het	GP	HN	HN	het	GP	HN	GP	GP	18	18	24	2.40	0.80	0.80
het	het	het	het	het	GP	GP	het	GP	HN	HN	HN	GP	HN	GP	GP	18	14	28	0.80	0.80	0.09
HN	het	het	het	het	GP	GP	het	GP	HN	HN	HN	GP	HN	GP	GP	19	14	27	1.43	1.42	0.09
HN	het	het	het	het	GP	GP	het	GP	het	HN	HN	GP	HN	GP	GP	18	15	27	0.90	0.80	0.00
HN	het	het	het	het	GP	GP	het	GP	het	HN	HN	GP	het	GP	GP	16	12	32	0.80	0.09	0.80
HN	het	het	het	het	GP	GP	het	GP	het	HN	HN	GP	het	GP	GP	16	12	32	0.80	0.09	0.80
HN	het	HN	het	het	GP	GP	het	GP	het	HN	HN	GP	het	GP	GP	17	12	31	0.90	0.36	0.80
HN	het	HN	het	het	GP	GP	het	GP	het	HN	HN	GP	het	GP	GP	16	14	30	0.13	0.09	0.09
HN	HN	HN	het	het	GP	GP	het	GP	het	HN	HN	GP	het	GP	GP	18	13	28	1.00	0.95	0.28
HN	HN	HN	het	het	GP	GP	het	GP	het	het	HN	GP	het	GP	GP	21	14	25	3.30	3.20	0.09
HN	het	HN	HN	GP	het	het	GP	GP	GP	het	HN	GP	HN	het	GP	14	16	30	0.13	0.09	0.09
HN	het	HN	HN	GP	het	het	het	GP	het	HN	HN	het	het	GP	het	15	13	32	0.40	0.00	0.36
HN	het	HN	HN	GP	het	het	het	GP	het	HN	HN	het	het	GP	het	16	12	32	0.80	0.09	0.80
het	het	het	het	HN	HN	GP	het	GP	GP	GP	het	GP	GP	GP	het	21	14	25	3.30	3.20	0.09

Table S2. Genotypes of F10 recombinant inbred lines derived from a cross between Golden Promise and Haruna Nijo.

Index	Chr	position (cM)	Name	Address	F10HGP003	F10HGP004	F10HGP005	F10HGP006	F10HGP007	F10HGP008	F10HGP009	F10HGP010	F10HGP011	F10HGP012	F10HGP013	F10HGP014	F10HGP015	F10HGP016	F10HGP017	F10HGP018
348	1H	0.95	337-641	5366	GP	HN	HN	HN	HN	GP	GP	GP	GP	GP	HN	GP	GP	GP	HN	GP
164	1H	1.51	6195-2137	2205	GP	HN	HN	HN	HN	GP	GP	GP	GP	GP	HN	GP	GP	GP	HN	GP
213	1H	23.86	1906-429	2915	HN	HN	GP	HN	HN	HN	GP	HN	GP	GP	GP	GP	HN	HN	HN	HN
261	1H	26.58	6081-850	3901	HN	HN	GP	HN	HN	HN	GP	HN	GP	GP	GP	GP	HN	HN	HN	HN
183	1H	33.61	3751-1136	2398	HN	HN	GP	HN	HN	HN	HN	HN	GP	GP	GP	GP	HN	HN	HN	HN
292	1H	40.99	5381-1950	4075	HN	HN	GP	GP	HN	HN	HN	HN	GP	GP	GP	GP	HN	HN	HN	HN
316	1H	43.28	6720-641	5052	HN	HN	GP	GP	HN	HN	HN	HN	GP	GP	GP	GP	HN	HN	HN	HN
176	1H	54.73	7284-710	2322	HN	HN	GP	GP	HN	HN	HN	HN	GP	GP	GP	GP	HN	HN	GP	HN
152	1H	65.53	3675-2615	2059	HN	HN	GP	GP	HN	HN	HN	HN	GP	GP	GP	GP	HN	HN	GP	HN
224	1H	99.19	3702-982	2999	HN	GP	GP	HN	HN	HN	GP	AB	GP	GP	AB	GP	AB	HN	GP	AB
160	1H	106.60	3786-2204	2166	HN	GP	HN	GP	HN	HN	GP	HN	HN	HN	GP	HN	HN	HN	GP	HN
105	1H	108.31	4962-1295	1601	HN	GP	HN	GP	HN	HN	GP	HN	HN	HN	GP	HN	HN	HN	GP	GP
131	1H	112.54	5690-1045	1841	HN	GP	HN	HN	HN	GP	GP	HN	HN	HN	GP	HN	HN	HN	GP	GP
265	1H	116.31	5048-1685	3919	HN	GP	HN	HN	HN	GP	GP	HN	HN	HN	HN	HN	HN	HN	HN	GP
98	1H	125.27	4978-1030	1550	HN	GP	HN	HN	HN	HN	GP	HN	HN	HN	HN	HN	HN	HN	HN	GP
379	1H	127.10	4393-1078	6110	HN	GP	HN	GP	HN	HN	GP	HN	HN	HN	HN	HN	HN	HN	HN	GP
116	1H	131.89	5555-438	1711	HN	HN	HN	GP	HN	HN	GP	HN	HN	HN	HN	HN	HN	HN	HN	GP
202	2H	10.94	3452-1355	2828	GP	GP	HN	HN	HN	HN	GP	GP	GP	GP	GP	HN	HN	HN	GP	GP
201	2H	18.20	4277-1901	2808	GP	GP	HN	HN	HN	HN	GP	GP	GP	GP	GP	HN	HN	HN	GP	GP
110	2H	21.61	1865-396	1661	GP	GP	HN	HN	HN	HN	GP	GP	GP	GP	GP	HN	HN	HN	GP	GP
296	2H	28.44	7747-1056	4107	GP	GP	HN	GP	HN	GP	GP	GP	GP	HN	GP	HN	HN	HN	GP	HN
335	2H	29.15	7032-201	5221	GP	GP	HN	GP	HN	GP	GP	GP	GP	HN	GP	HN	HN	HN	GP	HN
112	2H	37.32	1447-464	1682	GP	GP	HN	GP	HN	GP	GP	HN	GP	HN	GP	HN	HN	HN	HN	HN
358	2H	39.10	2964-382	5815	GP	GP	HN	GP	HN	GP	GP	HN	GP	HN	GP	GP	HN	HN	HN	HN
248	2H	41.66	4410-284	3606	GP	GP	HN	GP	HN	GP	GP	HN	GP	HN	GP	GP	HN	HN	HN	HN
102	2H	44.13	2651-1774	1588	GP	GP	HN	GP	HN	GP	GP	HN	GP	HN	GP	GP	HN	HN	HN	HN
108	2H	54.95	2580-1456	1644	GP	GP	HN	HN	HN	GP	GP	AB	GP	HN	GP	GP	HN	HN	GP	HN
66	2H	55.67	946-2500	1037	GP	GP	HN	HN	HN	GP	GP	HN	GP	HN	GP	GP	HN	HN	GP	HN
199	2H	57.54	8889-842	2794	GP	GP	HN	HN	HN	GP	GP	HN	GP	HN	GP	GP	HN	HN	GP	HN
194	2H	68.24	4434-804	2733	GP	GP	HN	HN	HN	GP	GP	HN	GP	GP	GP	GP	HN	HN	GP	HN
3	2H	71.56	7187-382	33	GP	GP	HN	HN	HN	GP	GP	HN	GP	GP	GP	GP	HN	HN	GP	HN
121	2H	73.04	334-1164	1772	GP	GP	HN	HN	HN	GP	GP	HN	GP	GP	GP	GP	HN	HN	GP	HN
109	2H	75.18	4377-571	1647	GP	GP	HN	HN	HN	GP	AB	HN	GP	GP	GP	GP	HN	HN	GP	HN
307	2H	78.03	1946-698	4950	GP	GP	HN	HN	HN	GP	GP	HN	GP	GP	GP	GP	HN	HN	GP	HN
132	2H	82.75	6117-1507	1850	GP	GP	HN	HN	HN	GP	GP	HN	GP	GP	GP	GP	HN	HN	GP	HN
354	2H	88.74	3469-1152	5784	GP	GP	HN	HN	HN	GP	GP	HN	GP	GP	GP	GP	HN	GP	GP	HN
273	2H	90.10	8632-1809	3979	GP	GP	HN	HN	HN	GP	GP	GP	GP	GP	GP	GP	HN	GP	GP	HN
195	2H	93.50	2020-539	2738	GP	GP	HN	HN	HN	GP	GP	HN	GP	GP	GP	HN	HN	GP	HN	HN
217	2H	108.61	ABC04580-1-4-420	2954	GP	HN	GP	HN	HN	GP	GP	HN	GP	HN	GP	HN	HN	GP	HN	GP
129	2H	112.91	111-499	1826	GP	HN	HN	HN	HN	GP	GP	HN	GP	HN	GP	HN	HN	GP	HN	GP
368	2H	115.08	3180-1771	5930	GP	HN	HN	HN	HN	GP	GP	HN	GP	HN	GP	HN	HN	GP	GP	GP
134	2H	117.91	7576-818	1876	GP	HN	HN	HN	HN	GP	GP	GP	GP	HN	GP	HN	HN	GP	GP	GP
339	2H	120.80	3256-1196	5285	GP	HN	HN	HN	HN	GP	GP	GP	GP	HN	GP	HN	HN	GP	GP	GP
137	3H	6.03	ConsensusGBS0194-	1888	GP	HN	GP	GP	HN	GP	GP	HN	GP	HN	HN	HN	GP	HN	HN	HN
94	3H	8.86	5945-748	1382	GP	HN	GP	GP	HN	GP	GP	GP	GP	HN	HN	HN	GP	HN	HN	GP
260	3H	46.31	4105-1417	3896	GP	GP	HN	GP	HN	HN	HN	GP	HN	HN	GP	HN	HN	GP	HN	HN
173	3H	59.89	1746-1527	2308	GP	GP	HN	GP	HN	HN	HN	GP	HN	HN	GP	HN	HN	GP	HN	HN
10	3H	62.99	5038-1035	113	GP	GP	HN	GP	HN	HN	HN	GP	HN	HN	GP	HN	HN	GP	HN	HN
67	3H	65.52	4184-393	1042	GP	GP	HN	GP	HN	HN	HN	GP	HN	HN	GP	HN	HN	GP	HN	HN
302	3H	78.53	1176-1547	4161	GP	GP	HN	HN	GP	HN	GP	GP	HN	HN	GP	HN	HN	GP	HN	HN
36	3H	88.82	8020-87	634	HN	GP	HN	GP	GP	GP	AB	GP	HN	HN	GP	HN	HN	GP	HN	HN
243	3H	123.68	76-1059	3484	HN	GP	HN	HN	GP	HN	HN	GP	HN	HN	HN	HN	GP	GP	HN	HN
29	3H	126.27	5260-462	515	HN	GP	HN	HN	GP	GP	HN	GP	GP	HN	HN	HN	GP	GP	HN	HN
294	3H	130.19	2335-1614	4092	HN	GP	HN	HN	GP	GP	HN	GP	GP	HN	HN	HN	GP	GP	HN	HN
123	3H	131.59	3718-1026	1777	HN	GP	GP	HN	GP	GP	GP	GP	HN	HN	HN	HN	GP	GP	GP	HN
289	3H	150.37	7818-967	4069	GP	HN	GP	GP	GP	HN	GP	GP	HN	HN	HN	HN	GP	GP	GP	HN
93	3H	155.09	13750-348	1375	GP	HN	GP	GP	GP	HN	NC	GP	HN	HN	HN	HN	GP	HN	GP	HN
49	4H	5.55	ABC14522-1-8-350	813	GP	HN	GP	HN	HN	HN	HN	GP	HN	HN	HN	GP	HN	GP	GP	HN

170	4H	21.61	2055-947	2277	GP	HN	GP	HN	HN	HN	HN	GP	HN	GP	HN	HN	GP	GP	HN	HN
233	4H	77.31	9149-1316	3072	HN	HN	GP	GP	GP	HN	HN	GP	HN	GP	HN	GP	GP	GP	HN	HN
216	4H	81.69	1523-1136	2934	HN	HN	GP	GP	GP	HN	HN	GP	HN	GP	HN	GP	GP	GP	HN	HN
77	4H	84.30	4986-1214	1225	HN	HN	GP	GP	GP	HN	HN	GP	HN	GP	HN	GP	GP	GP	HN	HN
175	4H	87.49	4564-604	2320	HN	HN	GP	GP	GP	HN	HN	GP	HN	HN	HN	GP	GP	GP	HN	HN
240	4H	92.38	4361-1867	3468	HN	HN	GP	GP	GP	HN	HN	GP	HN	HN	HN	GP	GP	GP	HN	HN
319	4H	98.55	4535-1366	5073	HN	HN	GP	HN	GP	HN	HN	GP	GP	HN	HN	GP	GP	GP	HN	HN
43	4H	100.74	41-695	732	HN	HN	GP	HN	GP	HN	HN	GP	GP	HN	HN	GP	GP	GP	HN	HN
209	4H	106.03	5692-310	2898	HN	HN	GP	HN	GP	HN	HN	GP	GP	HN	HN	GP	HN	GP	GP	HN
186	5H	25.23	4753-1091	2420	GP	GP	GP	GP	HN	GP	HN	GP	HN	HN	GP	HN	HN	GP	HN	HN
20	5H	30.99	8377-1022	233	GP	GP	GP	GP	HN	GP	HN	GP	HN	HN	HN	HN	HN	GP	HN	HN
214	5H	34.25	4684-775	2927	GP	GP	GP	GP	HN	GP	HN	GP	HN	HN	HN	HN	HN	GP	HN	HN
151	5H	53.18	8320-955	2057	GP	GP	GP	HN	HN	GP	GP	GP	HN	HN	HN	HN	HN	GP	HN	GP
76	5H	70.48	264-571	1173	GP	GP	GP	HN	HN	GP	GP	GP	HN	HN	HN	HN	HN	GP	HN	GP
205	5H	102.06	5004-375	2861	GP	GP	GP	GP	HN	GP	GP	GP	HN	HN	HN	HN	AB	GP	HN	NC
190	5H	122.38	139-1263	2501	HN	GP	HN	GP	HN	GP	AB	GP	GP	HN	HN	GP	HN	GP	HN	HN
167	5H	130.13	211-259	2232	HN	GP	HN	HN	HN	GP	AB	GP	HN	HN	HN	GP	HN	GP	HN	HN
263	5H	135.72	5571-640	3908	HN	GP	HN	HN	HN	HN	AB	GP	HN	HN	HN	GP	HN	GP	HN	HN
222	5H	137.16	1394-1222	2994	HN	GP	HN	HN	HN	HN	AB	GP	HN	HN	HN	GP	HN	GP	HN	HN
54	5H	147.40	3883-616	913	HN	GP	HN	HN	HN	HN	AB	NC	HN	HN	HN	GP	HN	GP	HN	HN
309	5H	155.13	ConsensusGBS0451-	4969	HN	HN	HN	HN	HN	HN	GP	GP	HN	HN	HN	GP	GP	GP	GP	GP
210	5H	159.09	603-72	2901	HN	HN	HN	HN	HN	HN	GP	GP	HN	HN	HN	HN	GP	GP	GP	GP
278	5H	171.66	ABC09278-1-4-69	4004	HN	HN	HN	HN	HN	HN	GP	HN	HN	HN	HN	HN	GP	GP	GP	GP
223	5H	191.97	2978-938	2995	HN	HN	GP	GP	GP	HN	GP	GP	GP	HN	GP	HN	GP	HN	GP	HN
57	5H	195.42	2726-852	950	HN	HN	GP	GP	GP	GP	GP	GP	GP	HN	GP	HN	GP	HN	GP	HN
200	6H	0.00	1692-742	2807	HN	GP	HN	HN	HN	GP	GP	HN	HN	HN	HN	HN	GP	GP	GP	GP
46	6H	1.34	5159-579	753	HN	GP	HN	HN	HN	GP	GP	HN	HN	HN	HN	HN	GP	GP	GP	GP
212	6H	3.11	ConsensusGBS0136-	2913	HN	GP	HN	HN	HN	GP	GP	HN	HN	HN	HN	HN	GP	GP	GP	GP
189	6H	12.54	ConsensusGBS0346-	2485	HN	HN	HN	HN	HN	GP	GP	HN	HN	GP	HN	HN	GP	GP	GP	GP
187	6H	16.97	1769-545	2442	HN	HN	HN	HN	HN	HN	HN	HN	HN	GP	GP	HN	GP	GP	GP	GP
120	6H	28.39	4611-178	1753	HN	HN	HN	HN	HN	GP	HN	HN	HN	GP	HN	HN	GP	HN	GP	GP
37	6H	31.73	5771-91	651	HN	HN	HN	HN	GP	GP	HN	GP	HN	GP	HN	HN	GP	HN	GP	GP
118	6H	48.74	3378-619	1746	HN	HN	HN	HN	GP	GP	HN	GP	GP	GP	GP	HN	GP	HN	GP	GP
117	6H	52.75	1009-1089	1740	HN	HN	HN	HN	GP	GP	HN	GP	GP	GP	GP	HN	GP	HN	GP	GP
249	6H	55.36	5232-2041	3625	HN	HN	HN	HN	GP	GP	HN	GP	GP	GP	GP	HN	GP	HN	GP	GP
305	6H	63.27	ConsensusGBS0369-	4908	HN	HN	GP	HN	GP	GP	HN	GP	GP	GP	GP	HN	GP	HN	GP	GP
333	6H	64.36	3348-395	5184	HN	HN	GP	HN	GP	GP	HN	GP	GP	GP	GP	HN	GP	HN	GP	GP
352	6H	67.04	4235-1617	5413	HN	HN	GP	HN	GP	GP	HN	GP	GP	GP	GP	HN	GP	HN	GP	GP
330	6H	72.54	1096-1482	5157	HN	HN	GP	HN	GP	GP	HN	GP	GP	GP	GP	HN	GP	HN	GP	GP
282	6H	77.89	7361-937	4022	HN	HN	GP	HN	GP	GP	HN	GP	GP	GP	GP	HN	GP	HN	GP	GP
295	6H	81.17	ConsensusGBS0239-	4099	HN	HN	GP	HN	GP	GP	HN	GP	GP	GP	GP	HN	GP	HN	GP	GP
124	6H	105.60	428-1519	1784	HN	GP	HN	HN	GP	HN	HN	GP	GP	GP	GP	HN	GP	HN	GP	GP
181	6H	110.32	2389-526	2361	HN	GP	GP	HN	GP	HN	GP	GP	GP	GP	GP	HN	GP	HN	GP	GP
372	6H	119.02	1852-509	5985	HN	GP	HN	HN	GP	HN	HN	GP	GP	GP	GP	HN	GP	HN	GP	GP
90	6H	126.85	6523-1691	1333	HN	GP	HN	HN	GP	HN	HN	GP	GP	GP	HN	HN	GP	HN	GP	GP
18	6H	129.38	3363-1795	209	HN	GP	HN	HN	HN	HN	HN	GP	GP	GP	HN	HN	GP	HN	HN	HN
206	7H	6.79	3359-1118	2862	HN	HN	HN	HN	HN	GP	GP	GP	HN	GP	HN	HN	GP	GP	HN	HN
172	7H	9.84	2148-498	2298	HN	HN	HN	HN	HN	GP	GP	GP	HN	GP	HN	HN	GP	GP	HN	HN
140	7H	17.20	9672-758	1935	GP	HN	HN	HN	HN	HN	GP	AB	HN	HN	HN	HN	GP	GP	HN	HN
55	7H	29.82	8365-454	915	HN	HN	GP	HN	GP	HN	GP	GP	HN	HN	HN	HN	GP	HN	HN	HN
381	7H	31.75	1404-64	6123	HN	HN	GP	HN	GP	HN	GP	GP	HN	HN	HN	HN	HN	HN	HN	HN
15	7H	34.82	5777-354	162	HN	HN	HN	HN	GP	HN	GP	GP	HN	HN	HN	HN	HN	HN	HN	HN
321	7H	46.19	ConsensusGBS0356-	5076	HN	HN	HN	GP	GP	GP	HN	GP	HN	HN	HN	HN	HN	GP	HN	HN
1	7H	52.82	1792-372	10	HN	HN	GP	HN	GP	GP	GP	GP	HN	HN	HN	HN	HN	GP	HN	HN
114	7H	56.81	12239-662	1689	GP	HN	GP	HN	GP	GP	HN	GP	HN	HN	HN	HN	HN	GP	HN	HN
81	7H	60.69	943-3107	1269	HN	HN	GP	HN	GP	GP	HN	GP	HN	HN	HN	HN	HN	GP	HN	HN
56	7H	63.66	11912-654	920	HN	HN	GP	HN	GP	GP	HN	HN	HN	HN	HN	HN	HN	GP	HN	HN
276	7H	68.46	4475-478	3987	HN	HN	GP	HN	GP	GP	HN	HN	HN	HN	HN	HN	HN	GP	HN	HN
349	7H	73.75	1735-1424	5381	HN	HN	GP	HN	GP	HN	HN	HN	HN	HN	HN	HN	HN	GP	HN	HN
148	7H	78.22	ABC09320-1-1-112	1990	HN	HN	GP	HN	GP	HN	HN	HN	HN	HN	HN	HN	HN	GP	HN	HN
58	7H	87.97	2462-971	952	HN	HN	GP	HN	GP	HN	HN	HN	HN	HN	HN	HN	HN	GP	HN	HN
182	7H	98.50	977-1377	2369	HN	GP	GP	HN	GP	HN	HN	GP	HN	HN	HN	HN	HN	GP	HN	HN
31	7H	122.07	ABC10197-1-1-101	585	HN	HN	HN	HN	HN	HN	HN	GP	GP	HN	GP	HN	GP	GP	HN	HN
5	7H	139.72	7023-448	54	GP	HN	GP	HN	HN	HN	GP	GP	GP	HN	GP	GP	HN	GP	HN	HN
13	7H	143.68	382-2624	153	GP	HN	GP	HN	HN	HN	GP	GP	GP	HN	GP	GP	HN	GP	HN	GP
113	7H	144.45	8785-443	1688	GP	GP	GP	GP	HN	GP	HN	HN	HN	HN	GP	HN	HN	GP	HN	HN

F10HGP019	F10HGP020	F10HGP021	F10HGP022	F10HGP023	F10HGP025	F10HGP026	F10HGP027	F10HGP028	F10HGP029	F10HGP030	F10HGP031	F10HGP032	F10HGP033	F10HGP034	F10HGP035	F10HGP036	F10HGP037	F10HGP038	F10HGP039	F10HGP040	F10HGP041	F10HGP042	F10HGP043	F10HGP044	F10HGP045	F10HGP046	F10HGP047
HN	GP	HN	HN	HN	HN	GP	GP	GP	GP	GP	HN	HN	HN	HN	GP	HN	HN	GP	GP	HN	HN	GP	GP	HN	GP	GP	GP
GP	GP	HN	HN	HN	HN	GP	GP	GP	GP	GP	HN	HN	HN	HN	GP	HN	HN	GP	GP	HN	HN	GP	GP	HN	GP	GP	GP
HN	GP	HN	HN	GP	GP	GP	GP	GP	HN	GP	HN	HN	HN	GP	HN	HN	HN	HN	GP	GP	HN	GP	HN	GP	GP	GP	HN
HN	GP	HN	HN	GP	GP	HN	GP	GP	HN	GP	HN	HN	HN	GP	HN	HN	HN	HN	GP	GP	HN	GP	HN	GP	GP	GP	HN
HN	GP	HN	HN	GP	GP	HN	GP	GP	HN	GP	HN	HN	HN	GP	HN	HN	HN	HN	GP	GP	HN	GP	HN	GP	HN	HN	HN
HN	GP	HN	HN	GP	GP	HN	GP	GP	HN	GP	HN	HN	HN	HN	HN	HN	HN	HN	GP	HN	HN	HN	HN	GP	HN	HN	HN
HN	GP	HN	HN	GP	GP	HN	GP	GP	HN	GP	HN	HN	HN	HN	HN	HN	HN	HN	GP	HN	HN	HN	HN	GP	HN	HN	HN
HN	GP	HN	HN	GP	GP	HN	GP	GP	HN	GP	HN	HN	GP	HN	HN	HN	HN	GP	GP	HN	HN	HN	HN	GP	HN	HN	HN
HN	GP	HN	HN	GP	GP	HN	GP	GP	HN	GP	HN	HN	HN	HN	HN	HN	HN	GP	GP	GP	HN	HN	AB	GP	HN	HN	HN
HN	HN	HN	GP	HN	GP	GP	HN	GP	HN	GP	HN	GP	GP	GP	HN	HN	HN	HN	GP	GP	GP	HN	HN	GP	HN	HN	HN
HN	HN	GP	GP	GP	HN	GP	HN	GP	GP	GP	GP	GP	HN	GP	GP	GP	HN	HN	GP	GP	GP	HN	GP	GP	HN	HN	HN
HN	HN	GP	GP	GP	HN	GP	HN	HN	GP	GP	GP	GP	HN	GP	GP	GP	HN	HN	GP	GP	GP	HN	GP	HN	HN	HN	HN
HN	HN	GP	GP	GP	HN	GP	HN	HN	GP	HN	GP	GP	HN	HN	GP	GP	HN	HN	GP	GP	GP	HN	GP	HN	HN	HN	HN
HN	HN	GP	GP	GP	HN	GP	HN	HN	GP	HN	GP	GP	HN	HN	HN	GP	HN	HN	HN	GP	GP	HN	HN	HN	HN	HN	HN
GP	HN	GP	GP	GP	HN	GP	GP	HN	GP	HN	GP	GP	GP	HN	GP	HN	HN	HN	HN	HN	GP	GP	GP	GP	HN	HN	HN
GP	HN	GP	GP	GP	HN	GP	GP	HN	GP	HN	GP	GP	GP	HN	GP	HN	HN	HN	HN	HN	GP	GP	GP	GP	HN	HN	HN
GP	HN	GP	GP	GP	HN	GP	GP	GP	GP	GP	GP	HN	HN	HN	GP	HN	HN	GP	HN	HN	HN	HN	GP	GP	HN	HN	HN
HN	HN	HN	HN	GP	HN	GP	HN	HN	GP	GP	GP	HN	HN	GP	GP	GP	HN	HN	HN	GP	AB	HN	GP	HN	HN	HN	GP
GP	HN	HN	HN	GP	HN	GP	HN	HN	GP	GP	GP	GP	HN	HN	HN	GP	HN	HN	HN	GP	GP	HN	HN	HN	HN	GP	GP
GP	HN	HN	HN	GP	HN	GP	GP	GP	GP	GP	GP	GP	HN	HN	GP	GP	HN	HN	HN	GP	GP	HN	HN	HN	HN	HN	GP
GP	HN	HN	HN	GP	HN	GP	GP	GP	GP	GP	GP	HN	HN	HN	GP	HN	GP	HN	GP	GP	HN	HN	HN	HN	HN	GP	GP
GP	HN	HN	HN	GP	HN	GP	GP	GP	GP	GP	GP	HN	HN	HN	GP	HN	GP	HN	GP	GP	HN	HN	HN	HN	HN	GP	GP
GP	HN	HN	HN	GP	HN	GP	GP	HN	GP	GP	HN	GP	HN	GP	GP	HN	HN	HN	HN	GP	HN	HN	HN	HN	HN	GP	GP
GP	HN	HN	HN	GP	HN	GP	GP	HN	GP	GP	HN	GP	HN	GP	GP	HN	HN	HN	HN	GP	HN	HN	HN	HN	HN	GP	GP
GP	HN	HN	HN	GP	HN	GP	HN	HN	GP	GP	HN	GP	HN	GP	GP	HN	HN	GP	HN	GP	HN	HN	HN	HN	HN	GP	GP
GP	HN	HN	HN	GP	HN	GP	HN	HN	GP	GP	HN	GP	HN	GP	GP	HN	HN	GP	HN	GP	GP	HN	HN	HN	HN	GP	GP
GP	HN	HN	HN	GP	HN	GP	HN	HN	GP	GP	HN	GP	HN	GP	GP	HN	HN	GP	HN	GP	GP	HN	HN	HN	HN	GP	GP
GP	HN	HN	HN	GP	HN	GP	HN	HN	GP	GP	HN	GP	HN	GP	GP	HN	HN	GP	HN	GP	GP	HN	HN	HN	HN	GP	GP
GP	HN	HN	HN	GP	HN	GP	HN	HN	GP	GP	HN	GP	HN	GP	GP	HN	HN	GP	HN	GP	GP	HN	HN	HN	HN	GP	GP
GP	HN	HN	HN	GP	HN	GP	HN	HN	GP	GP	HN	GP	HN	GP	GP	HN	HN	GP	HN	GP	GP	HN	HN	HN	HN	GP	GP
GP	HN	HN	HN	GP	HN	GP	HN	HN	GP	GP	HN	GP	HN	GP	GP	HN	HN	GP	HN	GP	GP	HN	HN	HN	HN	GP	GP
GP	HN	HN	HN	GP	HN	GP	HN	HN	GP	GP	HN	GP	HN	GP	GP	HN	HN	GP	HN	GP	GP	HN	HN	HN	HN	GP	GP
GP	HN	HN	HN	GP	HN	GP	HN	HN	GP	GP	HN	GP	HN	GP	GP	HN	HN	GP	HN	GP	GP	HN	HN	HN	HN	GP	GP
GP	HN	HN	HN	GP	HN	GP	HN	HN	GP	GP	HN	GP	HN	GP	GP	HN	HN	GP	HN	GP	GP	HN	HN	HN	HN	GP	GP
GP	HN	HN	HN	GP	HN	GP	HN	HN	GP	GP	HN	GP	HN	GP	GP	HN	HN	GP	HN	GP	GP	HN	HN	HN	HN	GP	GP
GP	HN	HN	HN	GP	HN	GP	HN	HN	GP	HN	GP	GP	HN	GP	GP	HN	HN	GP	HN	HN	GP	HN	GP	HN	HN	GP	GP
GP	HN	HN	HN	GP	HN	GP	HN	HN	GP	HN	GP	GP	HN	GP	GP	HN	HN	GP	HN	HN	GP	HN	GP	HN	HN	GP	GP
GP	HN	HN	HN	GP	HN	GP	HN	HN	GP	HN	GP	GP	HN	GP	GP	HN	HN	GP	GP	HN	GP	HN	GP	HN	GP	HN	GP
GP	HN	HN	GP	GP	HN	GP	HN	HN	HN	HN	GP	HN	HN	HN	HN	HN	HN	GP	GP	HN	HN	HN	GP	GP	GP	HN	HN
GP	HN	GP	GP	GP	HN	GP	HN	HN	HN	HN	GP	HN	HN	HN	GP	HN	HN	GP	GP	HN	HN	HN	GP	GP	GP	HN	HN
GP	HN	GP	GP	GP	HN	HN	HN	HN	HN	HN	GP	HN	HN	HN	GP	HN	HN	GP	GP	HN	HN	HN	GP	GP	GP	HN	AB
GP	HN	GP	GP	GP	HN	HN	HN	HN	HN	GP	GP	HN	GP	HN	GP	HN	HN	GP	GP	AB	HN	HN	HN	GP	GP	HN	GP
GP	GP	GP	GP	GP	HN	HN	HN	HN	HN	GP	GP	HN	GP	HN	GP	HN	HN	GP	GP	AB	HN	HN	HN	GP	GP	HN	GP
HN	HN	GP	GP	GP	GP	HN	HN	GP	GP	HN	GP	HN	GP	HN	HN	GP	HN	GP	GP	HN	GP	GP	GP	HN	HN	GP	HN
HN	HN	GP	GP	GP	GP	HN	HN	GP	AB	HN	GP	HN	GP	HN	HN	GP	HN	GP	GP	HN	GP	GP	GP	HN	HN	HN	HN
GP	HN	HN	GP	HN	HN	HN	GP	GP	HN	HN	HN	GP	HN	HN	HN	GP	HN	HN	GP	HN	HN	HN	HN	GP	HN	HN	HN
GP	HN	HN	GP	HN	HN	HN	GP	GP	HN	HN	HN	GP	HN	HN	HN	GP	HN	HN	GP	HN	HN	HN	HN	GP	HN	HN	HN
GP	HN	HN	GP	HN	HN	HN	GP	GP	HN	HN	HN	GP	HN	HN	HN	GP	HN	HN	GP	HN	HN	HN	HN	GP	HN	HN	HN
GP	GP	HN	GP	HN	HN	HN	GP	GP	HN	HN	HN	GP	HN	HN	HN	GP	HN	HN	GP	HN	HN	HN	HN	GP	GP	HN	HN
GP	GP	HN	GP	HN	HN	HN	GP	GP	HN	HN	HN	GP	HN	HN	HN	GP	HN	HN	GP	HN	HN	HN	HN	GP	GP	HN	HN
GP	GP	HN	HN	HN	HN	HN	GP	HN	HN	HN	HN	GP	GP	HN	HN	GP	GP	HN	GP	HN	HN	HN	HN	GP	HN	HN	HN
HN	HN	HN	HN	GP	GP	GP	HN	HN	HN	GP	HN	HN	HN	HN	GP	GP	GP	HN	GP	HN	HN	GP	HN	HN	HN	HN	HN
HN	HN	HN	HN	GP	GP	GP	HN	HN	GP	GP	HN	HN	HN	HN	GP	GP	GP	HN	GP	HN	GP	GP	HN	HN	HN	HN	HN
HN	HN	HN	HN	GP	HN	GP	HN	HN	GP	GP	HN	HN	HN	GP	GP	GP	HN	HN	GP	HN	GP	GP	HN	HN	HN	GP	HN
HN	GP	HN	HN	GP	HN	GP	HN	HN	GP	GP	HN	HN	HN	GP	GP	GP	HN	HN	GP	HN	GP	GP	HN	HN	HN	GP	HN
HN	GP	GP	HN	GP	HN	GP	HN	HN	HN	GP	HN	HN	GP	GP	GP	AB	GP	HN	GP	HN	HN	GP	HN	HN	HN	HN	HN
HN	GP	GP	HN	GP	HN	GP	HN	HN	HN	GP	HN	HN	GP	GP	GP	GP	GP	HN	GP	HN	HN	GP	HN	HN	GP	HN	GP
GP	GP	HN	GP	GP	HN	GP	GP	GP	HN	GP	HN	GP	GP	GP	HN	GP	GP	HN	GP	GP	GP	HN	HN	HN	HN	HN	GP

HN	GP	GP	HN	GP	GP	GP	HN	GP	HN	HN	GP	GP	GP	HN	HN	HN	HN	HN	HN	GP	HN	HN	GP	GP	HN	HN	HN
GP	GP	HN	GP	GP	GP	GP	GP	HN	GP	HN	GP	HN	GP	GP	HN	HN	HN	HN	HN	GP	HN	HN	GP	GP	HN	GP	GP
GP	GP	HN	GP	GP	GP	GP	HN	HN	GP	HN	GP	HN	GP	GP	HN	HN	HN	HN	HN	GP	HN	HN	HN	GP	HN	GP	GP
GP	GP	HN	GP	GP	GP	GP	HN	HN	GP	GP	GP	HN	GP	GP	HN	HN	HN	HN	HN	GP	HN	HN	HN	GP	HN	GP	GP
GP	GP	HN	GP	GP	GP	GP	HN	HN	GP	GP	GP	HN	GP	GP	HN	HN	HN	HN	HN	GP	HN	HN	HN	GP	HN	GP	GP
HN	GP	HN	GP	GP	GP	GP	GP	HN	GP	HN	GP	HN	HN	GP	GP	HN	HN	HN	HN	HN	HN	HN	HN	GP	HN	GP	GP
HN	GP	HN	GP	GP	HN	HN	GP	HN	GP	HN	GP	HN	HN	GP	GP	HN	HN	HN	HN	HN	HN	HN	GP	GP	HN	GP	GP
HN	GP	HN	GP	GP	HN	HN	HN	HN	GP	HN	GP	HN	HN	GP	GP	GP	HN	HN	HN	HN	HN	HN	GP	GP	HN	GP	GP
HN	GP	HN	GP	GP	HN	HN	HN	GP	GP	HN	GP	GP	HN	GP	GP	GP	HN	GP	GP	HN	HN	HN	HN	GP	GP	GP	GP

HN	HN	GP	HN	HN	HN	GP	HN	HN	GP	GP	HN	GP	GP	GP	GP	HN	HN	GP	GP	GP	HN	HN	GP	HN	HN	GP	HN
HN	HN	HN	HN	HN	HN	GP	HN	HN	GP	GP	HN	GP	HN	HN	GP	HN	HN	GP	HN	GP	HN	HN	GP	HN	HN	GP	GP
HN	HN	HN	HN	HN	HN	GP	HN	HN	GP	GP	HN	GP	HN	HN	GP	HN	HN	GP	HN	GP	HN	HN	GP	HN	HN	GP	GP
HN	HN	HN	HN	HN	HN	GP	HN	HN	GP	GP	GP	GP	HN	HN	GP	GP	HN	GP	HN	GP	HN	HN	GP	GP	HN	GP	GP
HN	HN	HN	HN	HN	HN	GP	HN	HN	GP	GP	GP	GP	HN	HN	GP	GP	HN	GP	HN	GP	HN	HN	GP	GP	HN	HN	GP
GP	HN	HN	HN	HN	HN	GP	HN	GP	GP	GP	GP	GP	HN	HN	GP	GP	HN	GP	HN	GP	HN	HN	GP	GP	HN	HN	GP
HN	HN	GP	HN	GP	HN	HN	HN	HN	HN	HN	HN	GP	HN	HN	HN	HN	GP	HN	GP	HN	HN	GP	HN	GP	HN	HN	GP
HN	HN	GP	HN	GP	HN	HN	HN	HN	HN	GP	HN	GP	GP	HN	HN	HN	GP	HN	GP	HN	GP	GP	HN	GP	HN	HN	GP
HN	HN	GP	HN	HN	GP	HN	HN	HN	HN	GP	HN	GP	GP	HN	HN	GP	HN	HN	GP	HN	GP	GP	HN	GP	HN	HN	GP
HN	HN	GP	HN	HN	GP	HN	HN	HN	HN	GP	HN	GP	GP	HN	HN	GP	HN	HN	GP	HN	GP	GP	HN	GP	HN	HN	GP
HN	HN	GP	GP	HN	GP	HN	HN	GP	HN	GP	GP	GP	GP	GP	HN	GP	HN	HN	HN	HN	GP	GP	HN	GP	GP	GP	GP
GP	HN	GP	GP	HN	GP	HN	GP	GP	HN	GP	GP	GP	GP	GP	GP	GP	HN	HN	HN	HN	HN	GP	HN	HN	GP	GP	GP
GP	HN	GP	GP	HN	GP	HN	GP	GP	HN	GP	GP	GP	GP	GP	GP	GP	HN	HN	HN	HN	GP	GP	GP	HN	GP	GP	GP
GP	HN	GP	GP	HN	GP	HN	HN	GP	HN	GP	GP	HN	GP	GP	HN	GP	HN	HN	HN	HN	GP	GP	GP	HN	GP	GP	GP
HN	HN	HN	GP	HN	GP	HN	HN	GP	HN	GP	GP	HN	HN	GP	HN	HN	GP	HN	HN	HN	HN	GP	HN	HN	GP	HN	HN
HN	HN	HN	GP	HN	GP	HN	GP	GP	HN	GP	GP	HN	HN	GP	HN	HN	GP	HN	HN	HN	HN	GP	HN	HN	GP	HN	HN

GP	GP	HN	HN	GP	GP	HN	HN	GP	GP	GP	HN	GP	HN	HN	GP	HN	HN	GP	GP	GP	GP	GP	HN	GP	GP	GP	GP
GP	GP	HN	HN	GP	GP	HN	HN	GP	GP	GP	HN	GP	HN	HN	GP	HN	HN	GP	GP	HN	GP	GP	HN	GP	GP	GP	GP
GP	GP	HN	HN	GP	GP	HN	HN	GP	GP	GP	HN	GP	HN	HN	GP	HN	HN	GP	GP	HN	GP	GP	HN	GP	GP	GP	GP
GP	GP	GP	HN	GP	GP	HN	HN	GP	GP	GP	HN	HN	HN	HN	GP	HN	HN	GP	GP	HN	GP	GP	HN	GP	GP	GP	GP
GP	HN	GP	HN	GP	HN	HN	HN	GP	GP	GP	HN	GP	HN	HN	GP	GP	HN	GP	GP	HN	HN	HN	HN	GP	GP	GP	GP
GP	GP	GP	HN	GP	HN	HN	GP	HN	HN	GP	HN	GP	HN	HN	GP	GP	HN	GP	HN	HN	GP	HN	GP	GP	GP	GP	HN
GP	GP	GP	HN	GP	HN	HN	GP	HN	GP	GP	GP	HN	GP	HN	GP	GP	HN	GP	HN	NC	GP	HN	GP	HN	GP	GP	HN
GP	GP	GP	HN	GP	HN	HN	GP	HN	GP	HN	GP	HN	HN	HN	GP	GP	HN	GP	HN	GP	HN	HN	GP	HN	GP	GP	HN
GP	GP	GP	HN	GP	HN	HN	GP	HN	GP	HN	GP	HN	HN	GP	GP	GP	HN	GP	HN	GP	HN	HN	GP	HN	GP	GP	HN
GP	GP	GP	HN	GP	HN	HN	GP	HN	GP	HN	GP	HN	HN	GP	GP	GP	HN	GP	HN	GP	HN	HN	GP	HN	GP	GP	HN
GP	GP	GP	HN	GP	HN	HN	GP	HN	GP	HN	GP	HN	GP	GP	GP	GP	HN	GP	HN	GP	HN	HN	GP	HN	GP	GP	HN
GP	GP	GP	HN	GP	HN	HN	GP	HN	GP	HN	GP	HN	GP	GP	GP	GP	HN	GP	HN	GP	HN	HN	GP	HN	GP	GP	HN
GP	GP	GP	HN	GP	HN	HN	GP	HN	GP	HN	GP	HN	GP	GP	GP	GP	HN	GP	HN	GP	HN	HN	GP	HN	GP	GP	HN
GP	GP	GP	HN	GP	HN	HN	GP	HN	GP	HN	GP	HN	GP	GP	GP	HN	HN	GP	HN	GP	GP	HN	GP	HN	GP	GP	HN
GP	GP	GP	HN	GP	HN	HN	GP	HN	GP	HN	GP	HN	GP	GP	GP	GP	HN	GP	HN	GP	GP	HN	GP	HN	GP	GP	HN
GP	GP	GP	HN	GP	HN	HN	GP	HN	GP	HN	GP	HN	GP	GP	GP	GP	HN	GP	HN	GP	GP	HN	GP	HN	GP	GP	HN
GP	HN	GP	HN	HN	GP	HN	GP	HN	HN	HN	GP	HN	HN	GP	HN	GP	HN	GP	HN	GP	GP	HN	HN	HN	GP	GP	GP
GP	HN	GP	HN	HN	GP	HN	GP	HN	HN	HN	GP	HN	HN	GP	HN	GP	HN	HN	HN	HN	GP	HN	HN	HN	HN	GP	GP
GP	GP	GP	HN	HN	HN	GP	GP	HN	HN	HN	GP	HN	HN	GP	HN	GP	HN	HN	HN	HN	GP	HN	HN	HN	HN	GP	HN
GP	GP	GP	HN	HN	HN	GP	GP	HN	HN	HN	HN	HN	GP	GP	HN	HN	HN	HN	HN	HN	GP	HN	HN	HN	GP	HN	HN
GP	GP	GP	HN	HN	HN	GP	GP	GP	HN	HN	HN	HN	GP	GP	GP	GP	HN	HN	HN	HN	HN	HN	HN	HN	GP	HN	HN

HN	GP	HN	HN	GP	HN	GP	GP	HN	GP	GP	HN	GP	HN	GP	HN	HN	GP	GP	GP	HN	GP	GP	GP	HN	GP	HN	HN
HN	GP	HN	HN	GP	HN	GP	GP	HN	GP	GP	HN	GP	HN	GP	HN	GP	GP	GP	GP	HN	GP	GP	GP	HN	GP	HN	HN
HN	GP	HN	HN	HN	GP	GP	GP	HN	GP	GP	HN	HN	HN	GP	HN	GP	GP	GP	HN	HN	GP	GP	GP	HN	GP	HN	HN
HN	GP	HN	GP	GP	GP	GP	HN	HN	GP	GP	HN	HN	GP	GP	HN	GP	HN	GP	HN	HN	GP	GP	GP	GP	GP	GP	GP
HN	GP	HN	GP	GP	GP	GP	HN	HN	GP	GP	HN	HN	GP	GP	HN	GP	HN	GP	HN	HN	GP	GP	GP	GP	GP	GP	GP
HN	GP	HN	GP	GP	GP	GP	HN	HN	GP	GP	HN	HN	GP	GP	HN	GP	HN	GP	HN	HN	GP	GP	GP	GP	GP	GP	GP
HN	GP	GP	GP	GP	GP	GP	HN	GP	GP	GP	HN	HN	GP	GP	GP	GP	HN	HN	GP	HN	HN	HN	GP	HN	HN	GP	GP
GP	GP	GP	GP	GP	GP	HN	HN	GP	GP	GP	HN	HN	GP	GP	GP	GP	HN	HN	GP	HN	HN	GP	HN	HN	HN	GP	GP
GP	GP	GP	GP	GP	GP	HN	HN	GP	GP	GP	HN	HN	GP	GP	GP	GP	HN	HN	GP	HN	HN	GP	HN	HN	HN	GP	GP
GP	GP	GP	GP	GP	GP	HN	HN	GP	GP	GP	HN	HN	GP	GP	GP	GP	HN	GP	GP	HN	HN	GP	HN	HN	HN	GP	GP
GP	GP	GP	GP	GP	GP	HN	HN	GP	GP	GP	HN	HN	GP	GP	GP	GP	HN	GP	GP	HN	HN	GP	HN	HN	HN	GP	GP
GP	GP	GP	GP	GP	GP	HN	HN	GP	GP	GP	HN	HN	GP	GP	GP	GP	GP	GP	GP	HN	HN	GP	HN	HN	HN	GP	GP
GP	GP	GP	GP	GP	GP	HN	HN	GP	GP	GP	HN	HN	GP	GP	GP	GP	GP	GP	GP	HN	HN	GP	HN	HN	GP	GP	GP
GP	GP	GP	GP	GP	GP	HN	HN	GP	GP	GP	HN	HN	GP	GP	GP	GP	GP	GP	GP	HN	HN	GP	HN	HN	GP	GP	GP
GP	GP	GP	GP	GP	GP	HN	HN	GP	GP	GP	HN	HN	GP	GP	GP	GP	GP	GP	GP	HN	HN	HN	HN	HN	GP	GP	GP
GP	GP	HN	GP	GP	GP	HN	HN	GP	GP	GP	HN	HN	GP	GP	GP	GP	GP	GP	HN	HN	HN	HN	HN	HN	GP	HN	GP
HN	HN	HN	GP	GP	GP	GP	HN	GP	GP	GP	HN	GP	HN	GP	GP	GP	GP	GP	HN	HN	HN	HN	HN	HN	GP	GP	HN
HN	HN	GP	GP	GP	GP	GP	HN	GP	HN	HN	HN	GP	GP	GP	GP	HN	GP	GP	HN	HN	HN	GP	HN	HN	HN	GP	HN
HN	HN	GP	HN	GP	GP	GP	GP	GP	HN	HN	HN	GP	GP	HN	GP	HN	GP	GP	HN	HN	HN	GP	HN	HN	HN	GP	HN
HN	HN	GP	HN	HN	HN	HN	HN	HN	GP	GP	HN	GP	GP	GP	GP	HN	HN	GP	GP	GP	HN	GP	GP	HN	HN	GP	HN

F10HGP048	F10HGP049	F10HGP050	F10HGP051	F10HGP052	F10HGP053	F10HGP054	F10HGP055	F10HGP056	F10HGP057	F10HGP058	F10HGP059	F10HGP060	F10HGP061	F10HGP062	F10HGP063	F10HGP064	F10HGP065	F10HGP066	F10HGP068	F10HGP069	F10HGP070	F10HGP071	F10HGP072	F10HGP073	F10HGP074	F10HGP075	F10HGP077
GP	GP	GP	HN	HN	HN	HN	GP	HN	GP	GP	HN	GP	HN	GP	GP	HN	GP	GP	HN	GP	HN	GP	GP	HN	HN	HN	HN
GP	GP	GP	HN	HN	HN	HN	GP	HN	GP	GP	HN	GP	HN	GP	GP	HN	GP	GP	HN	GP	HN	GP	GP	HN	HN	HN	HN
GP	HN	GP	HN	GP	GP	GP	GP	HN	GP	GP	HN	GP	HN	HN	HN	GP	GP	GP	HN	HN	HN	HN	HN	GP	GP	HN	HN
GP	HN	GP	HN	GP	GP	GP	GP	GP	GP	GP	HN	GP	HN	HN	HN	GP	GP	GP	HN	HN	HN	HN	HN	GP	GP	HN	HN
GP	HN	HN	HN	GP	GP	GP	HN	GP	HN	GP	HN	GP	GP	HN	HN	GP	HN	GP	GP	HN	HN	GP	HN	GP	GP	HN	HN
HN	HN	HN	HN	GP	GP	GP	HN	GP	HN	GP	GP	HN	GP	GP	HN	GP	HN	GP	GP	HN	HN	GP	HN	GP	GP	HN	GP
HN	HN	HN	HN	GP	GP	GP	HN	GP	HN	GP	GP	HN	GP	GP	HN	GP	HN	GP	GP	HN	HN	GP	HN	GP	GP	HN	GP
HN	HN	HN	HN	GP	GP	GP	HN	GP	HN	GP	GP	HN	GP	GP	HN	GP	HN	GP	GP	HN	HN	GP	HN	GP	GP	HN	GP
HN	GP	HN	HN	GP	GP	GP	HN	GP	HN	GP	HN	GP	GP	GP	HN	GP	HN	GP	GP	HN	HN	GP	HN	GP	GP	HN	GP
GP	GP	HN	GP	GP	GP	GP	GP	HN	HN	GP	HN	HN	GP	HN	GP	GP	HN	HN	HN	HN	HN	HN	GP	GP	GP	GP	HN
GP	HN	HN	GP	GP	GP	HN	GP	HN	HN	HN	HN	HN	GP	HN	GP	GP	HN	GP	HN	GP	HN	HN	GP	GP	GP	GP	HN
GP	HN	HN	GP	GP	GP	HN	GP	HN	HN	HN	GP	HN	GP	HN	HN	GP	HN	GP	HN	GP	HN	HN	GP	GP	GP	GP	HN
GP	GP	GP	GP	GP	GP	HN	GP	HN	HN	HN	GP	HN	GP	HN	HN	GP	HN	GP	HN	GP	HN	HN	GP	GP	GP	GP	HN
GP	GP	GP	GP	GP	GP	HN	GP	HN	HN	HN	GP	HN	GP	HN	HN	GP	HN	GP	HN	GP	HN	HN	GP	GP	GP	GP	HN
GP	GP	GP	GP	HN	HN	HN	GP	HN	HN	HN	GP	HN	GP	GP	HN	GP	HN	HN	HN	GP	HN	HN	GP	HN	HN	GP	HN
GP	GP	GP	GP	HN	HN	HN	GP	HN	HN	HN	GP	HN	GP	GP	HN	GP	HN	HN	GP	GP	HN	HN	GP	HN	HN	GP	HN
GP	GP	GP	GP	HN	HN	HN	HN	HN	HN	HN	GP	GP	GP	GP	HN	GP	HN	HN	GP	GP	GP	HN	HN	HN	GP	GP	HN
GP	GP	GP	HN	HN	HN	GP	HN	HN	HN	HN	GP	GP	GP	GP	GP	GP	HN	GP	HN	GP	GP	GP	HN	GP	GP	HN	GP
GP	GP	GP	HN	HN	HN	GP	HN	HN	HN	HN	GP	HN	GP	GP	GP	GP	HN	GP	HN	GP	GP	GP	HN	GP	GP	HN	GP
HN	GP	GP	HN	HN	HN	GP	HN	HN	HN	HN	GP	HN	GP	GP	GP	GP	HN	GP	HN	GP	GP	GP	HN	GP	GP	HN	GP
HN	HN	GP	HN	HN	HN	HN	HN	HN	HN	HN	GP	HN	GP	GP	GP	GP	HN	GP	HN	GP	GP	GP	HN	GP	HN	HN	GP
HN	HN	GP	HN	HN	HN	HN	HN	HN	HN	HN	GP	HN	GP	GP	GP	GP	HN	GP	HN	GP	GP	GP	HN	GP	HN	HN	GP
HN	HN	GP	HN	HN	HN	HN	HN	HN	HN	HN	GP	HN	HN	GP	GP	GP	HN	GP	HN	GP	HN	GP	HN	GP	HN	HN	GP
HN	HN	GP	HN	HN	HN	HN	HN	HN	HN	HN	GP	HN	HN	GP	GP	GP	HN	GP	HN	GP	HN	GP	HN	GP	HN	HN	GP
HN	HN	GP	HN	HN	HN	HN	HN	HN	HN	HN	GP	HN	HN	GP	GP	GP	HN	GP	HN	GP	HN	GP	HN	GP	HN	HN	GP
HN	HN	GP	HN	HN	HN	HN	HN	HN	HN	HN	GP	HN	HN	GP	GP	GP	HN	GP	HN	GP	HN	GP	HN	GP	HN	HN	GP
HN	HN	HN	HN	HN	HN	HN	HN	HN	GP	HN	GP	HN	HN	GP	GP	GP	HN	GP	HN	HN	HN	HN	HN	GP	HN	HN	GP
HN	HN	HN	HN	HN	HN	HN	HN	HN	GP	HN	GP	HN	HN	GP	GP	GP	HN	GP	HN	HN	HN	HN	HN	GP	HN	HN	GP
HN	HN	HN	HN	HN	HN	HN	HN	HN	GP	HN	GP	HN	HN	GP	GP	GP	HN	GP	HN	HN	HN	HN	HN	GP	HN	HN	GP
HN	HN	HN	HN	HN	HN	HN	HN	HN	GP	HN	GP	HN	HN	GP	GP	GP	HN	GP	HN	HN	HN	HN	HN	GP	HN	HN	GP
HN	HN	HN	HN	HN	HN	HN	HN	HN	GP	GP	GP	HN	HN	GP	GP	GP	HN	GP	HN	HN	HN	HN	GP	GP	HN	HN	GP
HN	HN	HN	HN	HN	HN	HN	HN	HN	GP	GP	GP	HN	HN	GP	GP	GP	HN	GP	HN	HN	HN	HN	GP	GP	HN	HN	GP
GP	HN	HN	HN	HN	HN	HN	HN	HN	GP	GP	GP	GP	HN	GP	GP	GP	HN	GP	HN	HN	HN	HN	GP	GP	HN	HN	HN
HN	HN	HN	HN	HN	HN	HN	HN	HN	GP	GP	GP	GP	HN	GP	GP	GP	HN	GP	HN	HN	HN	HN	GP	GP	HN	HN	HN
HN	HN	HN	HN	HN	HN	HN	GP	HN	GP	GP	GP	GP	HN	GP	GP	GP	HN	GP	HN	HN	HN	HN	GP	GP	GP	HN	HN
HN	HN	HN	HN	HN	HN	HN	GP	HN	GP	GP	GP	GP	HN	GP	GP	GP	HN	GP	GP	HN	HN	HN	GP	HN	GP	HN	GP
HN	HN	HN	HN	HN	HN	HN	GP	HN	GP	GP	GP	GP	HN	GP	GP	GP	HN	GP	GP	HN	HN	HN	GP	HN	GP	HN	GP
HN	HN	GP	HN	HN	HN	HN	HN	HN	HN	GP	GP	GP	HN	GP	GP	GP	HN	GP	GP	HN	HN	HN	GP	HN	GP	HN	GP
HN	HN	GP	HN	HN	HN	HN	HN	GP	HN	GP	GP	GP	HN	GP	GP	GP	HN	GP	HN	HN	GP	HN	GP	HN	GP	HN	HN
HN	HN	GP	HN	HN	HN	HN	HN	GP	HN	GP	GP	GP	HN	GP	GP	GP	HN	GP	HN	HN	GP	HN	GP	HN	HN	HN	HN
GP	HN	GP	HN	GP	GP	HN	GP	GP	GP	GP	HN	GP	HN	GP	GP	GP	HN	GP	GP	HN	GP	HN	GP	HN	HN	HN	HN
GP	HN	GP	HN	GP	GP	HN	GP	GP	GP	GP	HN	GP	HN	GP	GP	GP	HN	GP	HN	GP	GP	HN	GP	HN	HN	HN	HN
GP	HN	GP	HN	GP	GP	HN	GP	GP	GP	GP	HN	GP	GP	GP	GP	GP	HN	GP	HN	GP	GP	HN	GP	HN	GP	HN	HN
HN	GP	GP	GP	GP	GP	HN	GP	HN	HN	GP	HN	HN	GP	GP	GP	GP	HN	HN	GP	GP	GP	GP	HN	GP	HN	GP	HN
HN	GP	GP	HN	GP	GP	HN	GP	HN	HN	GP	HN	HN	GP	GP	GP	GP	HN	HN	GP	GP	GP	GP	HN	GP	HN	GP	HN
HN	HN	GP	HN	GP	GP	GP	HN	GP	GP	GP	GP	GP	HN	HN	HN	GP	GP	GP	HN	GP	HN	GP	HN	HN	HN	GP	HN
HN	HN	GP	HN	GP	GP	GP	HN	GP	GP	HN	GP	GP	HN	HN	HN	GP	GP	GP	HN	GP	HN	GP	HN	HN	HN	GP	HN
HN	HN	GP	HN	GP	GP	GP	HN	GP	GP	HN	GP	GP	HN	HN	HN	GP	GP	GP	HN	GP	HN	GP	HN	HN	HN	GP	HN
HN	HN	GP	HN	HN	HN	GP	HN	GP	GP	HN	HN	GP	HN	HN	HN	GP	GP	GP	HN	GP	HN	GP	HN	HN	GP	GP	HN
HN	HN	GP	HN	HN	HN	GP	HN	GP	GP	HN	HN	GP	HN	HN	HN	GP	GP	GP	HN	GP	HN	GP	HN	HN	GP	GP	HN
HN	HN	GP	HN	HN	HN	HN	HN	GP	HN	HN	HN	GP	HN	HN	HN	GP	GP	GP	HN	GP	HN	GP	HN	HN	GP	GP	HN
HN	HN	GP	HN	HN	HN	HN	HN	HN	GP	HN	HN	GP	HN	GP	HN	HN	HN	HN	HN	GP	HN	GP	GP	HN	GP	GP	GP
HN	HN	GP	HN	HN	HN	HN	HN	HN	GP	HN	HN	GP	HN	GP	HN	HN	HN	HN	HN	HN	HN	GP	GP	HN	GP	HN	HN
GP	HN	GP	HN	HN	HN	HN	HN	HN	GP	HN	HN	GP	GP	GP	HN	HN	HN	HN	HN	HN	HN	GP	GP	HN	GP	HN	HN
GP	HN	GP	HN	HN	HN	HN	HN	HN	GP	HN	HN	GP	GP	GP	GP	HN	HN	HN	HN	HN	HN	GP	GP	GP	GP	HN	HN
GP	HN	GP	HN	GP	GP	HN	HN	GP	GP	HN	HN	GP	HN	GP	GP	GP	GP	HN	HN	HN	HN	HN	GP	GP	GP	GP	HN
GP	HN	GP	HN	GP	GP	HN	HN	GP	GP	HN	HN	GP	GP	GP	GP	GP	GP	HN	HN	HN	HN	HN	HN	GP	GP	HN	HN
GP	HN	HN	HN	GP	GP	GP	HN	GP	GP	HN	HN	GP	GP	HN	HN	HN	HN	HN	GP	GP	HN	HN	GP	GP	GP	GP	HN

HN	GP	GP	GP	GP	GP	HN	HN	GP	HN	HN	HN	GP	GP	GP	HN	HN	HN	HN	GP	GP	HN	HN	GP	HN	GP	GP	HN
GP	GP	HN	HN	GP	GP	HN	HN	GP	HN	GP	GP	HN	GP	GP	GP	GP	HN	GP	GP	GP	HN	GP	GP	GP	HN	GP	GP
HN	GP	HN	HN	GP	GP	HN	HN	GP	HN	GP	GP	HN	GP	GP	GP	GP	AB	GP	GP	GP	HN	GP	GP	GP	HN	GP	GP
HN	GP	HN	HN	HN	HN	HN	HN	GP	HN	GP	GP	HN	GP	GP	GP	GP	HN	GP	GP	GP	HN	GP	GP	GP	GP	GP	GP
HN	GP	GP	HN	HN	HN	HN	HN	GP	HN	HN	GP	HN	GP	GP	GP	GP	HN	GP	GP	GP	HN	GP	GP	GP	GP	GP	GP
GP	HN	GP	GP	HN	HN	HN	HN	GP	HN	HN	HN	HN	GP	GP	GP	GP	HN	GP	HN	GP	HN	GP	HN	HN	GP	GP	GP
GP	HN	GP	GP	HN	HN	HN	HN	GP	HN	HN	HN	HN	GP	GP	GP	GP	HN	GP	HN	GP	HN	GP	HN	GP	HN	GP	GP
GP	HN	GP	GP	HN	HN	HN	HN	HN	HN	HN	HN	HN	GP	GP	GP	GP	HN	GP	HN	GP	HN	GP	HN	GP	HN	HN	GP
GP	HN	GP	GP	GP	GP	HN	GP	HN	GP	HN	HN	HN	GP	HN	GP	GP	HN	GP	HN	HN	HN	HN	HN	GP	HN	HN	GP

HN	GP	HN	HN	GP	GP	GP	GP	HN	HN	GP	HN	HN	HN	GP	GP	HN	HN	GP	GP	HN	GP	GP	HN	HN	HN	GP	GP
HN	GP	HN	GP	HN	HN	GP	GP	HN	HN	HN	HN	HN	HN	GP	GP	GP	HN	GP	GP	HN	GP	GP	HN	HN	HN	HN	GP
HN	GP	HN	GP	HN	HN	GP	GP	HN	HN	HN	HN	HN	HN	GP	GP	GP	HN	GP	GP	HN	GP	GP	HN	HN	HN	GP	GP
HN	HN	HN	GP	HN	HN	HN	HN	HN	HN	HN	HN	HN	HN	GP	GP	GP	HN	GP	GP	HN	HN	GP	HN	HN	GP	GP	HN
HN	HN	HN	GP	HN	HN	HN	HN	HN	HN	HN	HN	HN	HN	GP	GP	GP	HN	HN	GP	HN	HN	GP	HN	HN	GP	GP	HN
GP	HN	GP	GP	HN	HN	HN	GP	GP	HN	HN	HN	HN	HN	GP	GP	HN	HN	HN	GP	GP	HN	GP	GP	HN	HN	GP	GP
GP	HN	HN	HN	GP	GP	HN	GP	GP	GP	HN	HN	HN	HN	HN	HN	HN	GP	HN	GP	HN	HN	GP	GP	HN	HN	HN	GP
GP	HN	HN	HN	GP	GP	HN	GP	GP	GP	GP	HN	HN	HN	HN	HN	HN	GP	HN	GP	HN	HN	HN	GP	HN	HN	HN	GP
GP	HN	HN	HN	GP	GP	HN	HN	HN	GP	GP	HN	HN	HN	HN	HN	HN	GP	HN	GP	HN	HN	HN	HN	HN	HN	HN	GP
GP	HN	HN	HN	GP	GP	HN	HN	HN	GP	GP	HN	HN	HN	HN	HN	HN	GP	HN	GP	HN	HN	HN	HN	HN	HN	HN	GP
GP	HN	HN	HN	GP	GP	HN	HN	HN	GP	GP	HN	HN	GP	GP	HN	HN	GP	HN	GP	HN	GP	GP	HN	HN	HN	HN	GP
GP	HN	HN	HN	GP	GP	HN	HN	HN	GP	GP	HN	GP	GP	GP	HN	HN	HN	HN	GP	HN	GP	GP	HN	HN	HN	HN	GP
GP	HN	HN	HN	GP	GP	HN	HN	HN	GP	GP	HN	GP	HN	GP	HN	HN	HN	HN	GP	HN	GP	GP	HN	HN	HN	HN	GP
HN	HN	HN	HN	GP	GP	HN	GP	HN	HN	GP	HN	GP	HN	GP	HN	HN	HN	HN	HN	HN	GP	GP	HN	HN	HN	HN	GP
HN	HN	GP	HN	GP	GP	HN	GP	HN	HN	GP	HN	GP	GP	GP	HN	HN	HN	GP	HN	HN	GP	GP	HN	GP	HN	HN	GP
HN	HN	GP	HN	GP	GP	HN	GP	HN	HN	GP	HN	GP	GP	GP	HN	HN	HN	GP	HN	HN	GP	HN	HN	GP	HN	HN	GP

HN	GP	GP	HN	GP	GP	HN	HN	HN	GP	HN	GP	HN	GP	HN	GP	GP	GP	HN	GP	GP	HN	HN	GP	GP	GP	GP	HN
HN	GP	GP	HN	GP	GP	HN	HN	HN	GP	HN	GP	HN	GP	HN	GP	GP	GP	HN	GP	GP	HN	HN	GP	GP	GP	GP	HN
HN	GP	GP	HN	GP	GP	HN	HN	HN	GP	HN	GP	HN	GP	HN	GP	GP	GP	HN	GP	GP	HN	HN	GP	GP	GP	GP	HN
GP	GP	GP	HN	GP	GP	HN	HN	HN	GP	HN	GP	HN	GP	GP	GP	GP	GP	HN	GP	GP	HN	HN	GP	GP	GP	GP	HN
GP	GP	GP	HN	GP	GP	HN	HN	HN	GP	HN	GP	HN	GP	HN	GP	GP	GP	HN	GP	GP	HN	HN	GP	GP	GP	GP	HN
GP	GP	GP	HN	GP	GP	HN	HN	HN	GP	HN	GP	HN	GP	HN	GP	GP	GP	HN	HN	HN	HN	HN	GP	GP	GP	HN	HN
GP	GP	GP	HN	GP	GP	HN	HN	HN	GP	HN	GP	GP	GP	HN	GP	GP	GP	HN	HN	HN	HN	HN	GP	GP	GP	HN	GP
GP	HN	GP	GP	GP	GP	HN	HN	GP	GP	HN	GP	GP	GP	HN	GP	HN	GP	HN	HN	HN	HN	GP	GP	HN	GP	HN	GP
GP	HN	GP	GP	GP	GP	HN	HN	GP	GP	HN	GP	GP	GP	HN	GP	HN	GP	HN	HN	HN	HN	GP	GP	HN	GP	HN	GP
GP	HN	GP	GP	GP	GP	HN	HN	GP	GP	HN	GP	GP	GP	HN	GP	HN	GP	HN	HN	HN	HN	GP	GP	HN	GP	HN	GP
GP	HN	GP	GP	GP	GP	HN	HN	GP	GP	HN	GP	GP	HN	HN	GP	HN	GP	HN	HN	HN	HN	GP	GP	HN	GP	HN	GP
GP	HN	GP	GP	GP	GP	HN	HN	GP	GP	HN	GP	GP	HN	HN	GP	HN	GP	HN	HN	HN	HN	GP	GP	HN	GP	HN	GP
GP	HN	GP	GP	GP	GP	HN	HN	GP	GP	HN	GP	GP	HN	HN	GP	HN	GP	HN	HN	HN	HN	GP	GP	HN	GP	HN	GP
GP	HN	GP	GP	HN	HN	HN	HN	GP	GP	HN	GP	GP	HN	HN	GP	HN	GP	HN	HN	HN	HN	GP	GP	HN	GP	HN	GP
GP	HN	GP	GP	HN	HN	HN	HN	GP	GP	HN	GP	GP	HN	HN	GP	HN	GP	HN	HN	HN	HN	GP	GP	HN	GP	HN	GP
GP	HN	GP	GP	HN	HN	HN	HN	GP	GP	HN	GP	GP	HN	HN	GP	HN	GP	HN	HN	HN	HN	GP	GP	HN	GP	HN	GP
GP	HN	GP	GP	HN	HN	GP	HN	HN	GP	HN	GP	HN	HN	HN	HN	GP	GP	HN	HN	HN	HN	GP	GP	HN	GP	HN	GP
GP	HN	GP	GP	HN	HN	GP	HN	HN	GP	HN	GP	HN	HN	HN	HN	GP	GP	HN	HN	HN	HN	GP	GP	HN	GP	HN	GP
GP	GP	GP	GP	HN	HN	GP	HN	GP	GP	HN	HN	HN	GP	GP	HN	GP	GP	GP	GP	HN	HN	GP	HN	GP	GP	HN	HN
GP	GP	GP	GP	HN	HN	GP	HN	HN	GP	GP	HN	HN	GP	HN	HN	GP	GP	GP	GP	HN	HN	HN	HN	GP	GP	HN	HN
GP	GP	GP	GP	GP	GP	GP	HN	HN	GP	GP	GP	HN	GP	GP	HN	GP	GP	GP	GP	HN	HN	GP	HN	GP	GP	HN	HN

GP	HN	GP	HN	HN	HN	GP	HN	GP	GP	HN	GP	GP	GP	HN	GP	GP	HN	GP	HN	HN	HN	HN	HN	GP	HN	GP	GP
HN	HN	GP	HN	HN	HN	GP	HN	GP	GP	HN	GP	GP	GP	HN	GP	NC	HN	NC	HN	HN	HN	HN	HN	NC	HN	NC	NC
HN	HN	GP	HN	HN	HN	GP	HN	GP	HN	HN	GP	GP	GP	HN	GP	GP	HN	GP	HN	HN	HN	GP	HN	GP	HN	GP	GP
GP	GP	GP	HN	HN	HN	GP	HN	GP	HN	HN	HN	GP	GP	HN	HN	GP	HN	HN	HN	HN	HN	GP	HN	HN	GP	GP	GP
GP	GP	GP	HN	HN	HN	GP	HN	GP	HN	HN	HN	GP	GP	HN	HN	GP	HN	HN	HN	HN	HN	GP	HN	HN	GP	HN	GP
GP	GP	GP	HN	HN	HN	GP	HN	GP	HN	HN	HN	GP	GP	HN	HN	GP	HN	HN	HN	HN	HN	GP	HN	HN	GP	HN	GP
GP	GP	GP	HN	HN	HN	GP	HN	GP	GP	GP	GP	GP	HN	HN	HN	GP	HN	HN	HN	HN	HN	GP	GP	HN	GP	HN	GP
GP	GP	GP	HN	HN	HN	GP	GP	GP	GP	GP	GP	GP	HN	HN	HN	GP	HN	HN	HN	HN	HN	GP	GP	HN	GP	HN	GP
GP	GP	GP	HN	HN	HN	GP	GP	GP	GP	GP	GP	GP	HN	HN	HN	GP	HN	HN	HN	HN	HN	GP	GP	HN	GP	HN	GP
GP	GP	GP	HN	HN	HN	GP	GP	GP	GP	GP	HN	GP	HN	HN	HN	GP	HN	HN	GP	HN	HN	GP	GP	HN	GP	GP	GP
GP	GP	GP	HN	HN	HN	GP	GP	GP	GP	GP	HN	GP	HN	HN	HN	GP	HN	HN	GP	HN	HN	GP	GP	HN	GP	GP	GP
GP	GP	GP	HN	HN	HN	GP	GP	GP	GP	GP	HN	GP	HN	HN	HN	GP	HN	HN	GP	HN	HN	GP	GP	HN	GP	GP	GP
GP	GP	GP	HN	HN	HN	GP	GP	GP	GP	GP	HN	GP	HN	HN	HN	GP	HN	HN	GP	HN	HN	GP	GP	HN	GP	GP	GP
GP	GP	GP	HN	HN	HN	GP	GP	GP	HN	GP	HN	GP	HN	HN	HN	GP	HN	HN	GP	HN	HN	GP	GP	HN	GP	GP	GP
GP	GP	GP	HN	HN	HN	GP	GP	GP	HN	GP	HN	GP	HN	HN	HN	GP	HN	HN	GP	HN	HN	GP	GP	HN	GP	GP	GP
GP	GP	GP	HN	HN	HN	GP	GP	GP	HN	GP	HN	GP	HN	HN	HN	GP	HN	HN	HN	HN	HN	GP	GP	HN	GP	GP	GP
HN	HN	GP	HN	GP	GP	HN	GP	GP	GP	HN	HN	HN	HN	GP	HN	HN	HN	HN	HN	HN	HN	GP	GP	GP	HN	GP	GP
HN	GP	GP	GP	GP	GP	HN	GP	GP	GP	GP	HN	HN	GP	HN	GP	GP	HN	HN	HN	HN	GP	GP	GP	GP	GP	GP	GP
HN	GP	GP	GP	HN	HN	HN	GP	GP	GP	GP	GP	HN	GP	HN	GP	GP	HN	HN	HN	HN	GP	GP	GP	GP	GP	GP	GP
HN	GP	HN	HN	GP	GP	GP	GP	HN	GP	GP	HN	HN	GP	GP	GP	HN	HN	GP	GP	HN	GP	GP	HN	HN	HN	GP	GP

F10HGP078	F10HGP079	F10HGP080	F10HGP081	F10HGP082	F10HGP083	F10HGP084	F10HGP085	F10HGP086	F10HGP087	F10HGP088	F10HGP089	F10HGP090	F10HGP091	F10HGP092	F10HGP093	F10HGP094	F10HGP095	F10HGP096	F10HGP097	F10HGP098	F10HGP099	No. of HN	No. of GP	χ^2 value (1:1)
HN	GP	HN	GP	HN	HN	GP	HN	HN	HN	HN	GP	GP	GP	GP	GP	HN	GP	GP	GP	HN	GP	44	50	0.383
HN	GP	HN	GP	HN	HN	GP	HN	HN	HN	HN	GP	GP	GP	GP	GP	HN	GP	GP	GP	HN	GP	43	51	0.6809
HN	GP	HN	GP	HN	GP	HN	HN	HN	HN	GP	GP	HN	GP	GP	AB	HN	HN	GP	HN	HN	GP	50	43	0.5269
HN	GP	HN	GP	HN	GP	GP	GP	HN	HN	GP	GP	HN	NC	GP	AB	HN	HN	GP	HN	HN	GP	48	44	0.1739
GP	GP	HN	HN	HN	GP	GP	GP	HN	HN	GP	GP	HN	AB	GP	HN	HN	HN	GP	AB	HN	GP	52	40	1.5652
GP	GP	HN	HN	HN	GP	GP	GP	HN	HN	GP	GP	HN	GP	GP	HN	HN	HN	GP	HN	HN	HN	55	39	2.7234
GP	GP	HN	HN	HN	GP	GP	GP	HN	HN	GP	GP	HN	GP	GP	HN	HN	HN	GP	HN	HN	HN	55	39	2.7234
GP	GP	HN	HN	HN	GP	GP	GP	HN	HN	GP	GP	HN	GP	GP	HN	GP	HN	GP	HN	HN	HN	51	43	0.6809
GP	GP	HN	HN	HN	GP	GP	GP	HN	GP	GP	GP	HN	GP	GP	HN	GP	HN	GP	HN	HN	HN	48	45	0.0968
GP	HN	HN	GP	HN	GP	GP	HN	HN	HN	HN	GP	HN	GP	HN	HN	GP	GP	GP	HN	GP	HN	46	44	0.0444
GP	HN	HN	GP	HN	GP	HN	HN	GP	HN	HN	GP	HN	GP	HN	HN	HN	HN	GP	HN	HN	HN	51	43	0.6809
GP	HN	HN	GP	HN	GP	HN	HN	GP	HN	HN	GP	HN	GP	HN	HN	HN	GP	GP	HN	HN	HN	51	43	0.6809
GP	HN	HN	GP	HN	GP	HN	HN	GP	HN	HN	GP	HN	HN	HN	HN	HN	GP	GP	HN	HN	GP	51	43	0.6809
GP	HN	HN	GP	HN	GP	HN	HN	GP	HN	HN	GP	HN	GP	HN	HN	HN	GP	GP	HN	HN	GP	55	39	2.7234
GP	HN	HN	GP	HN	GP	AB	HN	GP	HN	HN	HN	HN	GP	HN	HN	HN	GP	GP	NC	HN	GP	54	38	2.7826
GP	HN	HN	GP	HN	GP	HN	HN	GP	HN	HN	HN	HN	GP	HN	HN	HN	GP	GP	HN	HN	GP	54	40	2.0851
GP	HN	HN	HN	HN	GP	HN	HN	GP	HN	HN	HN	HN	HN	HN	GP	HN	GP	GP	HN	HN	GP	56	38	3.4468
HN	HN	HN	HN	GP	GP	AB	HN	HN	GP	GP	GP	GP	GP	GP	HN	GP	GP	HN	GP	HN	GP	43	49	0.3913
HN	HN	HN	HN	HN	GP	HN	GP	HN	GP	GP	HN	HN	GP	GP	HN	GP	GP	HN	GP	GP	HN	47	47	0
HN	HN	HN	HN	HN	GP	HN	GP	HN	GP	HN	HN	HN	AB	GP	HN	GP	AB	HN	AB	GP	HN	47	44	0.0989
HN	HN	HN	HN	HN	GP	HN	GP	HN	GP	HN	HN	HN	GP	GP	HN	GP	HN	HN	HN	GP	HN	52	42	1.0638
HN	HN	HN	HN	HN	GP	HN	GP	HN	GP	HN	HN	HN	GP	GP	HN	GP	HN	HN	HN	GP	HN	52	42	1.0638
HN	HN	HN	HN	HN	GP	HN	GP	HN	GP	HN	GP	HN	NC	GP	HN	HN	HN	HN	HN	GP	HN	58	35	5.6882
HN	HN	HN	HN	HN	GP	HN	GP	HN	GP	HN	GP	HN	GP	GP	HN	HN	HN	HN	HN	GP	HN	57	37	4.2553
HN	HN	HN	HN	HN	GP	HN	GP	HN	GP	HN	GP	HN	GP	GP	HN	HN	HN	HN	HN	GP	HN	57	37	4.2553
HN	HN	HN	HN	HN	GP	HN	GP	HN	GP	HN	GP	HN	AB	GP	HN	HN	HN	HN	HN	GP	HN	56	37	3.8817
HN	HN	HN	HN	HN	GP	HN	GP	HN	GP	HN	GP	HN	AB	GP	HN	GP	HN	HN	HN	GP	HN	56	36	4.3478
HN	HN	HN	HN	HN	GP	HN	GP	HN	GP	HN	GP	HN	GP	GP	HN	GP	HN	HN	HN	GP	HN	57	37	4.2553
HN	HN	HN	HN	HN	GP	HN	GP	HN	GP	HN	GP	HN	GP	GP	HN	GP	HN	HN	HN	GP	HN	57	37	4.2553
HN	HN	HN	HN	HN	GP	HN	GP	HN	GP	HN	GP	HN	GP	GP	HN	GP	HN	HN	HN	GP	HN	56	38	3.4468
HN	HN	HN	HN	HN	GP	AB	GP	HN	GP	HN	GP	HN	GP	GP	HN	GP	HN	HN	HN	GP	HN	53	40	1.8172
HN	HN	HN	HN	HN	GP	HN	GP	HN	GP	HN	GP	HN	AB	GP	HN	GP	HN	HN	HN	GP	HN	54	39	2.4194
HN	HN	HN	HN	HN	GP	HN	GP	HN	GP	HN	GP	HN	AB	GP	HN	GP	HN	HN	HN	GP	HN	53	39	2.1304
HN	HN	HN	HN	HN	GP	HN	GP	HN	GP	HN	GP	HN	GP	GP	HN	GP	HN	HN	HN	GP	HN	54	40	2.0851
HN	GP	HN	HN	HN	GP	HN	GP	HN	GP	GP	GP	HN	HN	GP	HN	GP	HN	HN	HN	GP	GP	50	44	0.383
HN	GP	HN	HN	HN	GP	HN	GP	HN	GP	HN	GP	HN	GP	GP	HN	GP	HN	HN	HN	HN	GP	49	45	0.1702
HN	GP	HN	HN	HN	GP	HN	HN	HN	GP	HN	GP	HN	GP	GP	HN	GP	HN	HN	HN	HN	GP	49	45	0.1702
HN	GP	HN	HN	GP	HN	HN	HN	HN	GP	GP	GP	HN	GP	GP	HN	HN	HN	HN	HN	HN	GP	52	42	1.0638
HN	GP	HN	HN	GP	GP	HN	GP	HN	GP	GP	HN	GP	HN	GP	HN	HN	HN	HN	HN	HN	HN	56	38	3.4468
HN	GP	HN	HN	GP	GP	HN	GP	HN	GP	GP	HN	GP	HN	GP	HN	HN	HN	HN	HN	HN	HN	56	38	3.4468
HN	GP	HN	HN	GP	GP	HN	GP	HN	GP	HN	HN	GP	HN	GP	HN	HN	HN	HN	HN	HN	HN	51	42	0.871
HN	GP	HN	HN	HN	GP	HN	GP	HN	GP	HN	HN	GP	HN	GP	HN	HN	HN	HN	HN	HN	HN	49	44	0.2688
HN	GP	HN	HN	HN	GP	HN	GP	HN	GP	HN	HN	GP	HN	GP	HN	HN	HN	HN	HN	HN	HN	46	47	0.0108
GP	GP	GP	GP	GP	HN	GP	HN	GP	HN	GP	GP	GP	HN	GP	GP	HN	GP	GP	GP	GP	GP	38	56	3.4468
GP	GP	GP	GP	GP	HN	GP	HN	GP	HN	GP	GP	GP	HN	GP	GP	HN	GP	GP	GP	GP	GP	38	55	3.1075
GP	HN	HN	GP	GP	GP	HN	HN	GP	HN	GP	GP	GP	HN	GP	GP	GP	HN	HN	GP	HN	HN	53	41	1.5319
GP	HN	HN	GP	GP	GP	HN	HN	GP	HN	GP	GP	GP	HN	GP	GP	HN	HN	HN	GP	HN	HN	55	39	2.7234
GP	HN	HN	GP	GP	GP	HN	HN	GP	HN	GP	GP	GP	HN	GP	GP	HN	HN	HN	GP	HN	HN	55	39	2.7234
GP	HN	HN	GP	GP	GP	AB	HN	GP	HN	GP	GP	GP	HN	GP	GP	HN	HN	HN	GP	HN	HN	54	39	2.4194
GP	GP	HN	GP	GP	GP	HN	HN	GP	HN	GP	GP	HN	HN	GP	GP	HN	HN	HN	GP	HN	HN	54	40	2.0851
GP	HN	HN	GP	GP	GP	HN	GP	HN	HN	GP	GP	HN	HN	GP	GP	HN	HN	GP	GP	GP	HN	55	38	3.1075
GP	HN	HN	HN	GP	HN	HN	GP	GP	HN	GP	HN	HN	HN	GP	HN	GP	HN	GP	GP	GP	HN	60	34	7.1915
GP	HN	HN	HN	GP	HN	HN	GP	GP	HN	GP	HN	HN	HN	GP	HN	GP	HN	GP	GP	GP	HN	59	35	6.1277
GP	HN	HN	HN	GP	HN	HN	GP	GP	HN	GP	HN	HN	HN	GP	HN	GP	HN	GP	GP	GP	HN	57	37	4.2553
GP	HN	HN	HN	GP	HN	HN	GP	GP	HN	GP	HN	HN	HN	GP	HN	GP	HN	GP	GP	GP	HN	52	42	1.0638
GP	HN	HN	GP	HN	HN	HN	HN	HN	HN	HN	HN	HN	GP	GP	HN	GP	HN	GP	HN	GP	GP	50	43	0.5269
GP	HN	HN	GP	HN	HN	HN	GP	HN	GP	HN	HN	HN	GP	GP	HN	GP	HN	GP	GP	GP	GP	47	46	0.0108
HN	GP	HN	GP	HN	HN	HN	HN	GP	GP	GP	GP	GP	HN	GP	GP	HN	GP	GP	HN	GP	GP	44	50	0.383

HN	HN	HN	GP	GP	HN	HN	HN	GP	GP	HN	GP	GP	GP	GP	GP	HN	GP	GP	HN	GP	GP	49	45	0.1702
HN	HN	HN	HN	HN	GP	HN	HN	HN	GP	HN	GP	GP	GP	GP	GP	GP	GP	GP	GP	HN	HN	40	54	2.0851
AB	HN	HN	AB	GP	GP	GP	HN	AB	GP	HN	GP	GP	GP	GP	GP	GP	GP	GP	GP	HN	HN	37	53	2.8444
HN	HN	GP	HN	GP	GP	GP	HN	HN	GP	HN	GP	GP	GP	GP	GP	GP	HN	GP	GP	HN	HN	41	53	1.5319
HN	HN	GP	HN	GP	GP	GP	GP	HN	GP	HN	GP	GP	GP	GP	GP	GP	HN	GP	GP	HN	HN	41	53	1.5319
HN	HN	GP	HN	GP	GP	GP	GP	HN	GP	HN	HN	GP	GP	GP	GP	GP	HN	GP	GP	HN	HN	47	47	0
HN	HN	GP	HN	GP	GP	GP	GP	HN	GP	HN	HN	GP	GP	GP	HN	GP	HN	GP	GP	HN	HN	49	45	0.1702
HN	HN	GP	HN	GP	GP	GP	HN	HN	GP	HN	HN	GP	GP	GP	HN	GP	HN	GP	GP	HN	HN	52	42	1.0638
HN	HN	GP	HN	GP	GP	HN	GP	GP	HN	HN	HN	GP	AB	GP	HN	GP	AB	GP	AB	GP	HN	45	46	0.011
GP	HN	HN	GP	HN	HN	HN	HN	HN	GP	HN	GP	GP	GP	HN	HN	HN	HN	GP	GP	HN	GP	50	44	0.383
GP	HN	GP	GP	HN	GP	HN	HN	HN	HN	HN	GP	HN	HN	GP	HN	HN	GP	GP	GP	HN	GP	55	39	2.7234
GP	HN	GP	GP	HN	GP	HN	HN	HN	HN	HN	GP	HN	HN	GP	HN	HN	GP	GP	GP	HN	GP	54	40	2.0851
GP	HN	GP	GP	HN	GP	HN	HN	HN	HN	HN	GP	HN	HN	GP	HN	HN	HN	GP	GP	HN	GP	55	39	2.7234
GP	HN	GP	GP	HN	GP	HN	HN	HN	HN	HN	HN	HN	HN	GP	HN	HN	HN	GP	HN	HN	GP	59	35	6.1277
GP	HN	HN	GP	HN	GP	HN	HN	HN	GP	HN	GP	GP	NC	HN	HN	HN	GP	GP	GP	GP	GP	45	46	0.011
GP	HN	GP	GP	GP	HN	HN	HN	GP	GP	HN	HN	GP	HN	HN	HN	HN	GP	GP	HN	GP	GP	56	37	3.8817
GP	HN	HN	GP	GP	HN	HN	HN	GP	HN	GP	HN	GP	HN	HN	GP	HN	HN	GP	HN	HN	GP	57	36	4.7419
GP	HN	HN	GP	GP	HN	HN	HN	GP	HN	HN	HN	GP	AB	HN	GP	HN	HN	GP	HN	HN	GP	61	31	9.7826
GP	HN	HN	GP	GP	GP	HN	HN	GP	HN	HN	HN	GP	NC	HN	GP	HN	NC	GP	AB	HN	GP	58	32	7.5111
GP	HN	HN	GP	GP	GP	HN	HN	HN	HN	HN	GP	HN	HN	HN	HN	HN	HN	GP	HN	HN	GP	54	38	2.7826
HN	HN	HN	GP	GP	GP	HN	HN	HN	HN	HN	GP	HN	HN	HN	HN	HN	HN	GP	HN	GP	GP	51	43	0.6809
HN	HN	HN	GP	GP	GP	GP	HN	HN	HN	HN	GP	HN	HN	HN	HN	HN	HN	GP	HN	GP	GP	50	44	0.383
GP	GP	HN	GP	GP	GP	GP	HN	HN	HN	HN	GP	HN	HN	HN	HN	HN	NC	GP	NC	GP	GP	52	40	1.5652
GP	GP	GP	HN	GP	HN	GP	HN	HN	HN	GP	GP	GP	GP	GP	GP	GP	GP	GP	HN	GP	GP	47	47	0
GP	GP	GP	HN	GP	HN	HN	HN	HN	HN	GP	GP	GP	GP	GP	GP	GP	GP	HN	HN	GP	GP	48	46	0.0426
HN	GP	HN	HN	HN	GP	HN	GP	GP	HN	GP	GP	HN	HN	GP	HN	HN	GP	HN	GP	HN	HN	44	50	0.383
HN	GP	HN	HN	HN	GP	HN	GP	GP	HN	GP	GP	HN	HN	GP	HN	HN	GP	HN	GP	HN	HN	45	49	0.1702
HN	GP	HN	HN	HN	GP	HN	GP	GP	GP	GP	GP	HN	HN	GP	HN	HN	GP	HN	GP	HN	HN	44	50	0.383
HN	GP	HN	HN	HN	GP	HN	GP	GP	HN	GP	GP	HN	HN	GP	HN	HN	GP	GP	HN	HN	GP	42	52	1.0638
HN	GP	HN	HN	HN	GP	HN	GP	GP	HN	GP	HN	HN	NC	GP	HN	HN	HN	GP	HN	HN	GP	47	46	0.0108
GP	HN	HN	GP	HN	GP	HN	GP	GP	HN	GP	GP	HN	HN	GP	HN	HN	HN	HN	HN	HN	HN	52	42	1.0638
GP	HN	HN	GP	HN	GP	HN	GP	GP	HN	GP	GP	HN	NC	HN	HN	HN	AB	HN	HN	HN	HN	45	46	0.011
GP	GP	HN	GP	HN	HN	GP	GP	HN	HN	GP	GP	HN	HN	GP	HN	HN	GP	HN	HN	GP	HN	45	49	0.1702
GP	GP	HN	GP	HN	HN	GP	GP	HN	HN	GP	GP	HN	HN	GP	HN	HN	GP	HN	HN	GP	HN	44	50	0.383
GP	GP	HN	GP	AB	HN	GP	GP	HN	HN	GP	GP	HN	HN	GP	HN	HN	GP	HN	HN	GP	HN	43	50	0.5269
GP	GP	HN	GP	HN	HN	GP	GP	HN	HN	GP	GP	HN	HN	GP	HN	HN	HN	HN	HN	GP	HN	44	50	0.383
GP	GP	HN	GP	HN	HN	GP	GP	HN	HN	GP	GP	HN	HN	GP	HN	HN	HN	HN	HN	GP	HN	44	50	0.383
GP	GP	HN	GP	HN	HN	GP	GP	HN	HN	GP	GP	HN	HN	GP	HN	HN	HN	HN	HN	GP	HN	44	50	0.383
GP	GP	HN	GP	HN	HN	GP	GP	HN	HN	GP	GP	HN	HN	GP	HN	HN	HN	HN	HN	GP	HN	46	48	0.0426
GP	GP	HN	GP	HN	HN	GP	GP	HN	HN	GP	GP	HN	HN	GP	HN	HN	HN	HN	HN	GP	HN	45	49	0.1702
GP	GP	HN	GP	HN	HN	GP	GP	HN	HN	GP	GP	HN	HN	GP	GP	HN	HN	HN	HN	GP	HN	44	50	0.383
HN	GP	GP	GP	HN	HN	GP	HN	HN	HN	GP	GP	GP	HN	HN	GP	HN	HN	HN	GP	GP	HN	50	44	0.383
HN	GP	GP	GP	HN	HN	GP	HN	GP	HN	GP	GP	GP	HN	GP	GP	HN	HN	HN	GP	GP	HN	49	45	0.1702
GP	GP	HN	HN	GP	HN	GP	HN	GP	GP	GP	HN	GP	HN	GP	GP	GP	HN	HN	GP	HN	HN	47	47	0
GP	GP	GP	HN	GP	HN	HN	HN	GP	GP	GP	HN	GP	NC	GP	GP	GP	HN	HN	HN	HN	HN	51	42	0.871
GP	GP	GP	GP	GP	HN	HN	HN	GP	HN	GP	GP	GP	HN	GP	GP	GP	HN	HN	HN	HN	HN	47	47	0
HN	HN	HN	GP	HN	GP	HN	GP	HN	GP	HN	GP	GP	GP	HN	GP	HN	GP	GP	HN	GP	HN	48	46	0.0426
HN	HN	HN	NC	HN	NC	HN	NC	HN	GP	HN	GP	HN	GP	GP	GP	HN	GP	GP	HN	GP	HN	48	38	1.1628
HN	HN	HN	GP	HN	GP	HN	GP	HN	GP	GP	HN	HN	GP	GP	GP	HN	GP	GP	HN	GP	HN	51	42	0.871
HN	HN	HN	GP	HN	GP	GP	HN	HN	HN	GP	HN	HN	GP	GP	HN	GP	GP	GP	AB	GP	HN	48	45	0.0968
HN	HN	HN	GP	HN	HN	GP	HN	HN	HN	GP	HN	HN	AB	HN	HN	HN	NC	GP	HN	GP	HN	54	38	2.7826
HN	GP	HN	GP	HN	HN	GP	HN	HN	HN	GP	HN	HN	GP	HN	HN	HN	HN	GP	HN	GP	HN	55	39	2.7234
HN	GP	HN	GP	GP	HN	GP	HN	GP	HN	GP	HN	HN	GP	GP	HN	HN	HN	GP	GP	GP	HN	47	47	0
AB	GP	AB	GP	GP	HN	GP	HN	GP	HN	GP	HN	HN	GP	GP	HN	HN	HN	HN	GP	HN	HN	45	47	0.0435
AB	GP	AB	HN	GP	HN	GP	HN	GP	HN	GP	HN	HN	GP	GP	HN	HN	HN	HN	GP	HN	HN	46	46	0
HN	GP	HN	HN	GP	HN	GP	HN	GP	HN	GP	HN	HN	GP	GP	HN	HN	HN	HN	GP	HN	HN	47	47	0
HN	GP	GP	HN	GP	HN	GP	HN	GP	HN	GP	HN	HN	GP	GP	HN	HN	HN	HN	GP	HN	HN	47	47	0
HN	GP	GP	HN	GP	HN	GP	HN	GP	HN	GP	HN	HN	AB	GP	HN	HN	HN	HN	GP	HN	HN	46	47	0.0108
HN	GP	GP	HN	GP	HN	GP	HN	GP	HN	GP	HN	HN	GP	GP	HN	HN	HN	HN	GP	HN	HN	46	48	0.0426
HN	GP	GP	HN	GP	HN	GP	HN	GP	HN	GP	HN	HN	GP	GP	HN	HN	HN	HN	GP	HN	HN	47	47	0
HN	GP	GP	HN	GP	HN	GP	HN	GP	HN	GP	HN	HN	GP	GP	HN	HN	HN	HN	GP	HN	HN	48	46	0.0426
GP	HN	GP	HN	GP	HN	GP	HN	GP	HN	GP	GP	HN	GP	GP	HN	HN	AB	GP	NC	HN	HN	47	45	0.0435
GP	HN	GP	HN	GP	HN	HN	HN	HN	HN	GP	HN	GP	GP	GP	HN	HN	HN	GP	GP	HN	HN	53	41	1.5319
HN	HN	GP	GP	HN	HN	GP	HN	HN	GP	GP	HN	GP	GP	GP	GP	HN	GP	GP	GP	HN	HN	41	53	1.5319
HN	HN	GP	GP	HN	HN	GP	GP	HN	GP	HN	HN	GP	GP	GP	GP	GP	GP	GP	GP	HN	HN	41	53	1.5319
GP	HN	HN	GP	HN	HN	HN	HN	HN	GP	AB	GP	GP	GP	HN	HN	HN	HN	GP	GP	HN	GP	48	45	0.0968

Table S3. Analysis of the allelic interactions in *TFA4*, *TFA7* and *TFA9* in transgenic HN×GP plants.

Loci	Genotype			χ^2	df	(ratio)	χ^2	df	(ratio)	χ^2	df	(ratio)
	GP	HE	HN									
TFA4	10	27	23	6.23 *	2	(1:2:1)	5.69 *	1	(3:1=[GP+HE]:[HN])	2.22	1	(1:3=[GP]:[HE+HN])
TFA7	8	35	17	4.37	2	(1:2:1)	0.36	1	(3:1=[GP+HE]:[HN])	4.36 *	1	(1:3=[GP]:[HE+HN])
TFA9	12	26	22	4.40	2	(1:2:1)	4.36 *	1	(3:1=[GP+HE]:[HN])	0.80	1	(1:3=[GP]:[HE+HN])
	GP/GP	HE/HE	HN/HN									
TFA4&7	1	15	5	2.43	2	(1:4:1)						
TFA4&9	3	11	9	8.57 *	2	(1:4:1)						
TFA7&9	3	14	6	1.57	2	(1:4:1)						
	GP/GP/GP	HE/HE/HE	HN/HN/HN									
TFA4,7&9	0	6	3	5.78	2	(1:8:1)						

*:Statistically significant by Chi-square test ($p<0.05$)
